# Genetic variation determines VEGF-A plasma levels in cancer patients

**DOI:** 10.1038/s41598-018-34506-4

**Published:** 2018-11-05

**Authors:** Federico Innocenti, Chen Jiang, Alexander B. Sibley, Amy S. Etheridge, Ace J. Hatch, Stefanie Denning, Donna Niedzwiecki, Ivo D. Shterev, Jiaxing Lin, Yoichi Furukawa, Michiaki Kubo, Hedy L. Kindler, J. Todd Auman, Alan P. Venook, Herbert I. Hurwitz, Howard L. McLeod, Mark J. Ratain, Raluca Gordan, Andrew B. Nixon, Kouros Owzar

**Affiliations:** 1 UNC Eshelman School of Pharmacy, University of North Carolina at Chapel Hill, Lineberger Comprehensive Cancer Center, Chapel Hill, North Carolina USA; 2 Duke Cancer Institute, Duke University Medical Center, Durham, North Carolina USA; 3 Duke Human Vaccine Institute, Durham, North Carolina USA; 4 Department of Biostatistics and Bioinformatics, Duke University Medical Center, Durham, North Carolina USA; 5 Center for Genomic Medicine, RIKEN, Yokohama, Kanagawa Prefecture Japan; 6 RIKEN Center for Integrative Medical Sciences, Yokohama, Kanagawa Japan; 7 University of Chicago Comprehensive Cancer Center, Chicago, Illinois USA; 8 Department of Pathology and Laboratory Medicine, UNC School of Medicine, University of North Carolina at Chapel Hill, Chapel Hill, North Carolina USA; 9 University of California at San Francisco, San Francisco, California USA; 10 Moffitt Cancer Center, Tampa, Florida USA

## Abstract

Angiogenesis is essential in tumor biology and is regulated by vascular endothelial growth factor (VEGF) ligands and receptors. Here we aimed to discover genetic variants associated with levels of circulating angiogenic proteins in cancer patients. Plasma was collected at baseline in 216 pancreatic and 114 colorectal cancer patients. Thirty-one angiogenic proteins were measured by ELISA. 484,523 Single Nucleotide Polymorphisms (SNP) were tested for association with plasma levels for each protein in pancreatic cancer patients. Three top-ranked hits were then genotyped in colorectal cancer patients, where associations with the same proteins were measured. The results demonstrated rs2284284 and MCP1 (P-value = 6.7e–08), rs7504372 and VEGF-C (P-value = 9.8e–09), and rs7767396 and VEGF-A (P-value = 5.8e–09) were SNP-protein pairs identified in pancreatic cancer patients. In colorectal cancer patients, only rs7767396 (A > G) and VEGF-A was validated (P-value = 5.18e–05). The AA genotype of rs7767396 exhibited 2.04–2.3 and 2.7–3.4-fold higher VEGF-A levels than those with AG and GG genotypes. The G allele of rs7767396 reduces binding of the NF-AT1 transcription factor. In conclusion, a common genetic variant predicts the plasma levels of VEGF-A in cancer patients through altered binding of NF-AT1.

## Introduction

Angiogenesis is an essential event in tumor growth, progression, and metastasis, and is regulated by the VEGF pathway. Several angiogenic factors have been identified, among which VEGF appears to be the key regulator in neovascularization and enhanced vascular permeability^1,2^. The VEGF family consists of VEGF-A, -B, -C, -D, -E, and the placental growth factor (PlGF). The VEGF receptors comprise three tyrosine kinases: VEGFR-1 (FLT1), VEGFR-2 (KDR), and VEGFR-3 (FLT).

VEGF-A and VEGFR-2 are considered the most critical of the endothelial cell ligands and receptors, respectively, in solid tumor angiogenesis^3,4^. Anti-angiogenic therapies are established treatment modalities for many types of cancer^5,6^. Despite the survival advantage experienced by patients, this treatment strategy still faces many barriers. Among them, the paucity of clinically useful biomarkers that can consistently predict clinical efficacy for this class of agents is a significant impediment.

For certain cancers, the degree of angiogenesis may reflect the malignant potential of individual tumors^7,8^. The degree of angiogenesis can be quantified using various approaches, such as assessing tumor specimens for microvessel density or measuring tumor levels of angiogenic growth factors and cytokines. Approaches using patient blood are also available, including measurement of circulating angiogenic proteins and genetic analysis of germline DNA.

Circulatory proteins involved with angiogenesis and inflammation are measured to gain insight into the pathophysiology of different conditions. The intrinsic value of this approach is that it provides a safe and efficient way to identify circulatory proteins that reflect the biology of the tumor and its microenvironment. In the setting of oncogenesis, due to overproduction of VEGF-A by tumor cells relative to normal cells, circulating VEGF-A levels in cancer patients might reflect VEGF-A release by both normal and cancer cells. VEGF-A levels, hence, can inform about the biology of spreading tumors. For example, circulating VEGF-A levels decrease after primary tumor resection in colorectal and gastric cancers^9,10^, and there is a positive relationship between the concentrations of circulating VEGF-A and the extension of epithelial carcinomas^11^. Circulating proteins could provide information regarding the angiogenic potential of a tumor, or act as tools to evaluate residual disease after surgery, or early indications of response to anti-angiogenic therapy^12,13^.

The heritability of circulating angiogenesis proteins has primarily been evaluated for VEGF-A in family studies of cardiovascular disease and is high^14–16^. Genetic markers that predict the circulating levels of angiogenesis proteins in cancer patients have not been well-studied. To perform such study is of importance, as the level of heritability in cancer patients might be lower (or even very low) compared to non-cancer individuals. In fact, cancer patients tend to have higher VEGF-A levels as compared to non-cancer individuals, due to the influence of tumor-related VEGF-A production from multiple sources^17,18^.

Hence, we employed a genome-wide approach to discover genetic variants associated with levels of circulating angiogenesis proteins in cancer patients prior to receipt of treatment. As replication of these findings was crucial, we performed two studies (one discovery and one validation) from two clinical trials of cancer patients with two different diseases. An intergenic variant flanking the *VEGFA* gene was found to predict plasma levels of VEGF-A in both studies. Because the mechanistic basis of genetic associations needs to be established, we have also discovered that the the NF-AT1 transcription factor binds where the intergenic variant is located and its binding is affected by the variant.

## Results

### Associations between gene variants and plasma protein levels

**Figure 1:**
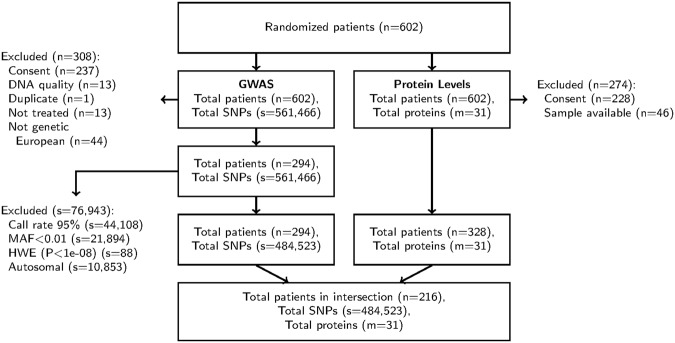
CONSORT chart for CALGB 80303. Abbreviations: MAF, minor allele frequency; HWE, Hardy-Weinberg Equilibrium.

**Figure 2:**
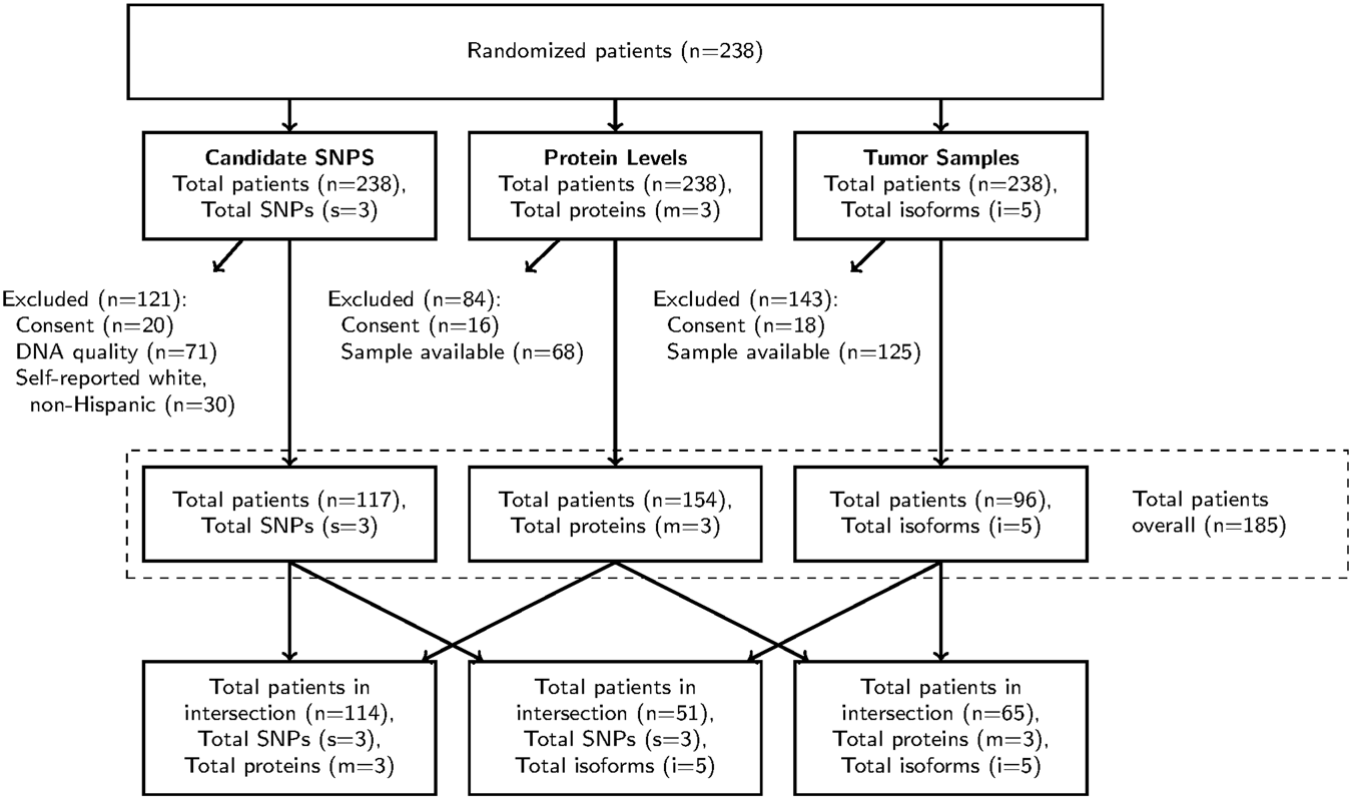
CONSORT chart for CALGB 80203. Abbreviations: MAF, minor allele frequency; HWE, Hardy-Weinberg Equilibrium.

The Consolidated Standards of Reporting Trials (CONSORT)^19^ charts presented in Figs 1 and 2 provide detailed information about the number of patients, proteins, and SNPs measured in the discovery (CALGB 80303) and validation (CALGB 80203) cohorts, respectively. CALGB is now a part of the Alliance for Clinical Trials in Oncology. For each of the 31 proteins in the discovery cohort, the top 50 SNPs (ranked according to P-value) are listed in Supplementary File 1.

Three SNP-protein pairs (rs7767396, VEGF-A), (rs2284284, MCP1) and (rs7504372, VEGF-C) with a P-value less than 1e–07 were selected for validation in CALGB 80203. This unadjusted P-value cut-off was used only for the purpose of selecting variants for validation. The results for these three pairs are summarized in Table 1. The quantile-quantile (QQ) and Manhattan plots, across all SNPs for each of these three proteins, are provided in Supplementary Figs 1 and 2 respectively.

**Table 1:** SNPs associated with plasma levels of MCP1, VEGF-C, and VEGF-A in CALGB 80303.

Protein	SNP	Gene	5′ flanking	3′ flanking	Base	MAF	Median protein levels (pg/ml) for 0, 1, or 2 minor alleles	P-value
MCP1	rs2284284	*NRCAM*	*NRCAM*	*PNPLA8*	A > G	0.292	592.9, 476.0, 395.0	6.7e–08
						0.286	863.8, 936.3, 917.5	0.481
VEGF-C	rs7504372	—	*TMEM200C*	*L3MBTL4*	T > C	0.086	494.1, 1350.0, 291.4	9.8e–09
						0.096	1286.7, 1577.0, NA	0.314
VEGF-A	rs7767396	—	*LOC100132354*	*C6orf223*	A > G	0.470	195.3, 95.7, 58.1	5.8e–09
						0.522	529.5, 230.4, 199.4	5.2e–05

“MAF” stands for minor allele frequency. For MAF, median levels, and P-values, the top values are from CALGB 80303 (discovery cohort) and the bottom values are from CALGB 80203 (validation cohort). P-values for Hardy-Weinberg Equilibrium in the discovery cohort are 0.25, 0.50, and 1.00 for rs2284284, rs7767396, and rs7504372, respectively. In the validation cohort, the P-values were 0.04, 0.19, and 0.60 respectively. Due to limitations in DNA quantity or quality, genotypes for rs7767396 and rs7504372 could not be determined for two and three patients, respectively, in CALGB 80203.

**Figure 3:**
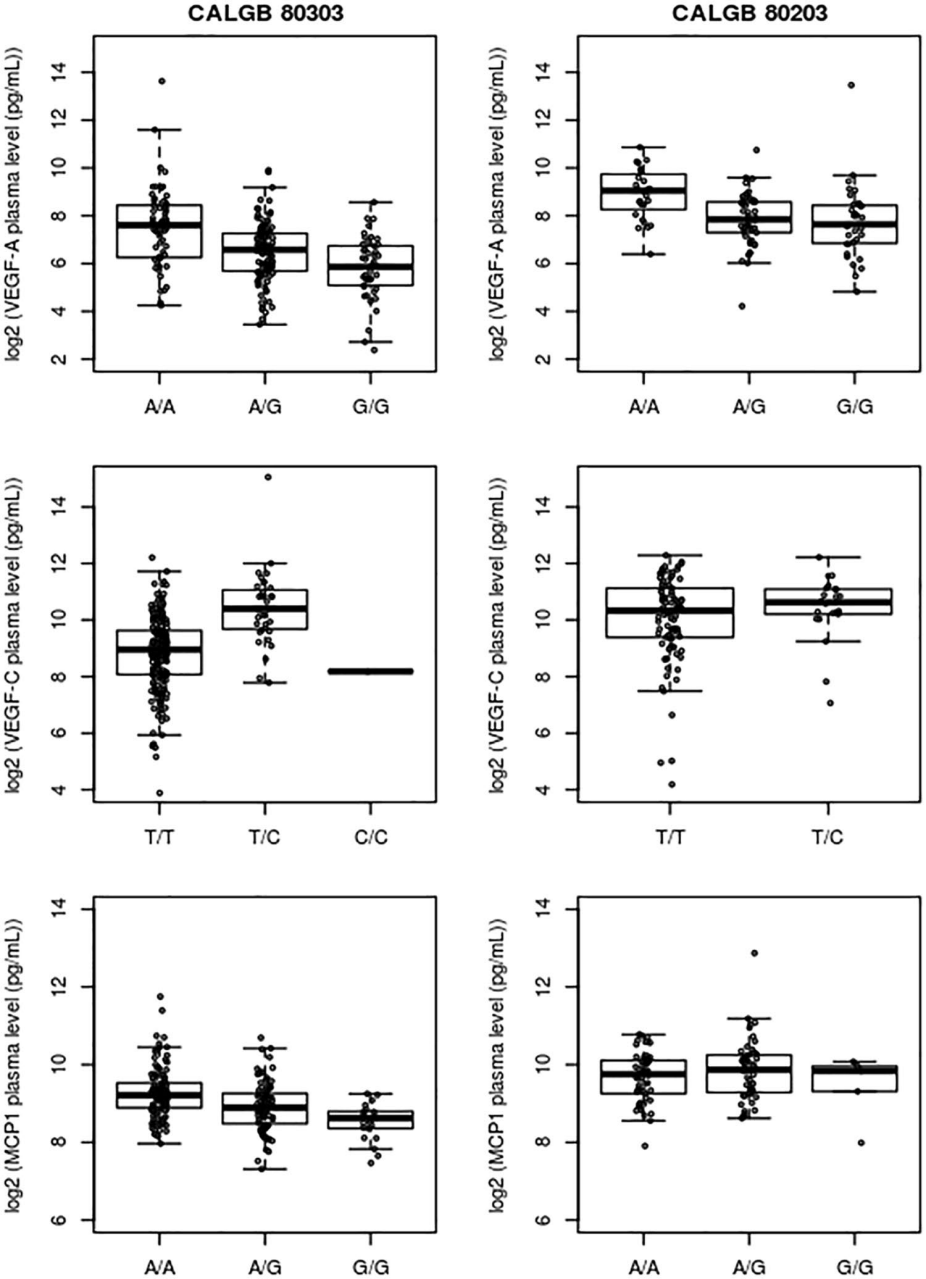
Associations between rs2284284 and MCP1 levels, rs7504372 and VEGF-C levels, and rs7767396 and VEFG-A levels in both CALGB 80303 and 80203. Boxes represent 25^th^ (Q1) and 75^th^ (Q3) percentiles; Horizontal lines indicate the medians; Upper whiskers indicate min(max(x), Q3 + 1.5 * IQR); Lower whiskers indicate max(min(x), Q1–1.5 * IQR). Points indicate observations.

The genotype effect of rs7767396 (A > G) on plasma levels of VEGF-A was validated in the CALGB 80203 cohort (P-value = 5.18e–05) based on our validation criterion. In both the discovery and validation cohorts, the G allele of rs7767396 reduces the plasma levels of the VEGF-A protein, and an apparent gene-dosage effect is observed, i.e., in CALGB 80303, the AA genotype exhibited 2.04 and 3.4-fold higher VEGF-A levels than those with AG and GG genotypes, respectively. In CALGB 80203, the corresponding fold increases were 2.3 and 2.7, respectively. The associations of rs2284284 with MCP1 (P-value = 0.314) and rs7504372 with VEGF-C (P-value = 0.481) were not statistically significant in the validation cohort. The distributions of the levels of the three plasma proteins, conditional on the genotypes, are shown in the boxplots in Fig. 3. Within the framework of a multivariable regression model, with age and gender as baseline covariates, the association between rs7767396 and VEGF-A level remained strong for both CALGB 80303 (P-value = 1.3e–08) and CALGB 80203 (P-value = 4.5e–04). The percentage of variance in VEGF-A levels explained by rs7767396 was 14.5% for CALGB 80303 and 13.9% for CALGB 80203. Compared to cancer patients with the AA genotype, patients with the G allele (AG and GG genotypes) of rs7767396 have 56% and 57% reductions in median VEGF-A levels, in CALGB 80303 and CALGB 80203, respectively.

### Associations between either rs7767396 or plasma VEGF-A levels and *VEGFA* mRNA levels in the tumor

The data from the validation study (CALGB 80203) show no evidence to support the hypothesis that the variability in the *VEGFA* mRNA levels in the primary tumors, as quantified by any of the four isoforms (121, 145, 165, and 189) individually or the total *VEGFA* mRNA levels, is associated with either the circulating VEGF-A levels in plasma or the rs7767396 germline variant (Supplementary Fig. 3).

### Transcription factor binding and relationship with rs7767396

**Figure 4:**
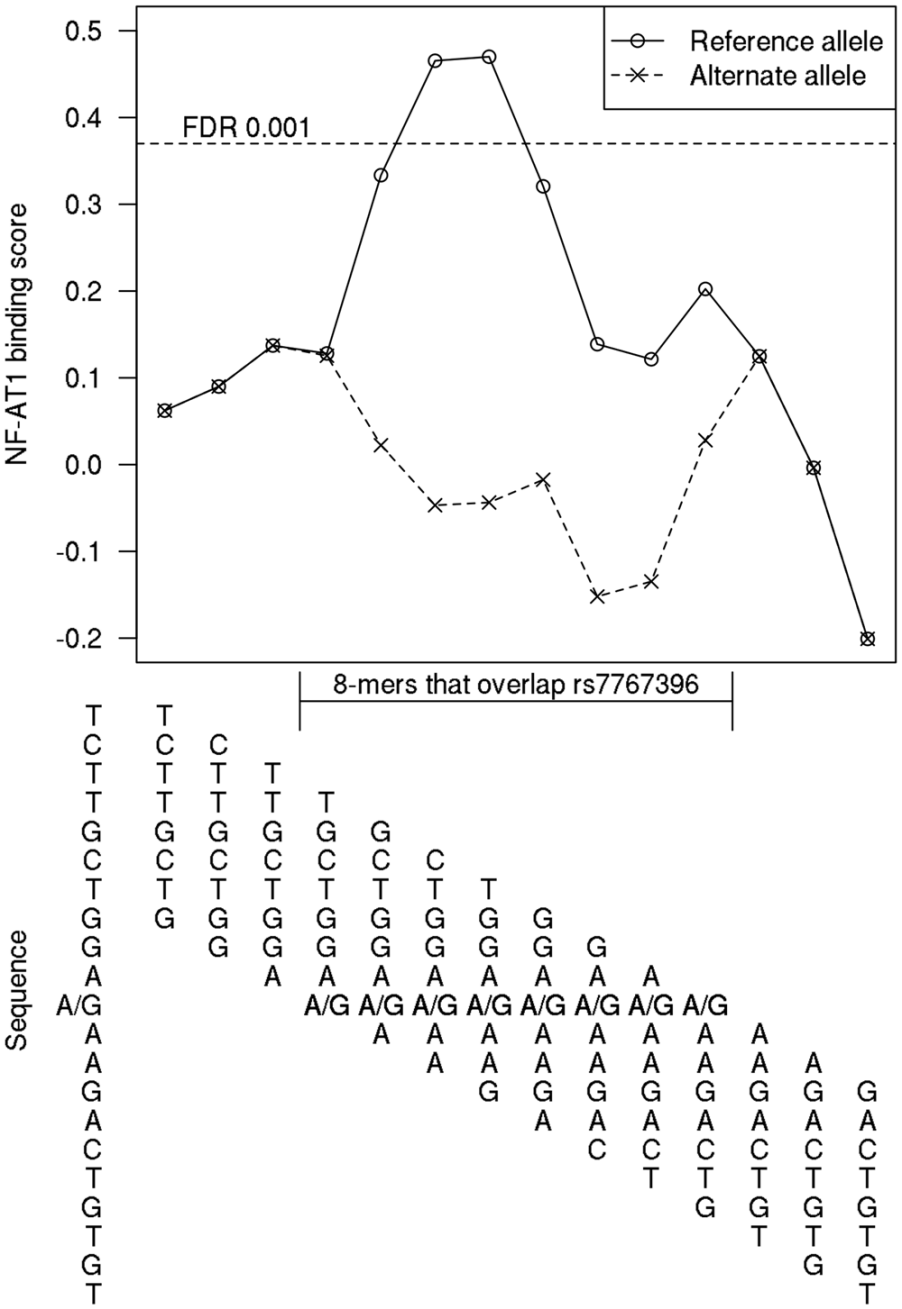
rs7767396 disrupts NF-AT1-DNA binding *in vitro*. Plot shows the NF-AT1 8-mer PBM data for a 21-bp genomic region centered at rs7767396. The reference allele is bound specifically by the protein, as indicated by the fact that it overlaps two 8-mers with a binding score above 0.37 (dotted line), which corresponds to a false discovery rate (FDR) of 0.001 for calling transcription factor binding sites^46^. The alternate allele is not bound specifically by NF-AT1 *in vitro*, as indicated by the fact that all 8-mers overlapping the allele have low binding scores.

A bioinformatics analysis was conducted to explore potential mechanisms for the observed association between rs7767396 and VEGF-A plasma levels. HaploReg indicates that the binding of NF-AT1 and ZBRK1 transcription factors may be altered by the presence of the rs7767396 variant, based on the DNA binding motifs of the two factors. For the NF-AT1 transcription factor, DNA binding specificity data is also available for all possible 8-bp sequences from the cis-BP database^15^. The corresponding protein binding microarray (PBM) data to confirm ZBRK1 is not available. Analysis of rs7767396 using these data (Fig. 4) shows that the reference allele is bound with high affinity by NF-AT1, while the alternative allele is not bound specifically by the protein.

## Discussion

The present study has assessed, for the first time, the genomic basis of differences in levels of circulatory proteins among cancer patients. Using a discovery/validation approach, a common, heritable variant was associated with plasma levels of VEGF-A in two different clinical trials of patients with two types of cancers.

This variant, rs7767396, is located 173 Kbs downstream from *VEGFA* and could be regarded as a *cis* protein quantitative trait locus (pQTL) for circulating levels of VEGF-A. Its relative minor allelic frequencies in the CALGB 80303 and 80203 studies are 0.47 and 0.52, respectively, compared to 0.49 reported for the 1000 Genomes Project 1 CEU population^20^. In both CALGB 80303 and 80203, the association between this variant and circulating VEGF-A levels is strong, with median differences in circulating VEGF-A levels of 2–3 folds across genotypes.

Debette *et al*. have evaluated the genetic basis of heritability in VEGF-A levels in non-cancer individuals^21^. For their validation cohort, they report four common variants (rs6921438, rs4416670, rs6993770, and rs10738760) that explain up to 48% of the heritability of serum VEGF-A levels. Although rs7767396 is not among those four variants, they report the results for the top hit of the present study, rs7767396, from their discovery set. In Debette *et al*., rs7767396 was associated with VEGF-A levels with an estimated effect size of beta = −0.71 and a P-value < 1.71e–482^21^. The estimated effects in our study are concordant, namely that copies of the G allele are associated with lower circulating VEGF-A levels. It is of interest to note that while only rs10738760 and a proxy for rs4416670, rs3734693, were genotyped in CALGB 80303, there was no evidence for linkage disequilibrium between rs7767396 and rs10738760 (r^2^ = 0 in CALGB 80303 and 1000 G CEU), or rs7767396 and rs3734693 (r^2^ = 0 in CALGB 80303 and r^2^ = 0.01 in 1000 G CEU). However, rs6921438 is in high LD with rs7767396 in 1000 G CEU (r^2^ = 0.93), and it’s likely that rs7767396 is the causal variant of that haplotype, due to its mechanism elucidated by our results.

Choi *et al*. report a genome-wide association study (GWAS) meta-analysis consisting of six discovery cohorts to identify SNPs associated with circulating VEGF-A levels^22^. They identify a panel of 10 SNPs, including three SNPs from the Debette *et al*. panel, which they report as accounting for 52% of the variability in circulating VEGF-A. For the top hit of the present study, they report a P-value of <4.85e–1284^22^. The reported effect size in each of their cohorts is positive with respect to the A allele, i.e. the A allele is associated with an increase in VEGF-A levels. Again, this is concordant with the direction of the effect size of rs7767396 in our study. Sun *et al*.^23^ report results from a pQTL GWAS using blood protein analytes in current and former smokers with or without chronic obstructive pulmonary disease (COPD). They report rs7767396 as the top variant for *VEGFA* (P-value = 5e–26). They do not report the direction of the effect.

For the first time, we report that rs7767396 predicts levels of circulating VEGF-A also in patients with cancer, in addition to its prediction in patients without cancer, as shown by the studies reported above. This novel finding is of relevance, as cancer patients tend to have higher VEGF-A levels as compared to non-cancer patients, probably due to the influence of somatic and tumor-specific factors^9,17,18,24,25^. This study cannot establish a biological link between the plasma levels of VEGF-A and the expression of the gene in tumor cells. In fact, rs7767396 in VEGF-A does not predict for mRNA levels of *VEGFA* in the tumor. This lack of association does not contradict the pQTL observation. It suggests that other factors, very likely of somatic nature and/or less heritable, control the variability of mRNA levels of *VEGFA* in the tumor of colorectal cancer patients. In addition to the possibility that technical noise is introduced by fixation of tumor cells with formalin (which can affect mRNA stability and quality), another explanation could be that *VEGFA* mRNA levels in the tumor cells might not be reflective of the expression in the endothelial cells. Additional studies should shed light on the genetic and environmental basis of *VEGFA* expression in the tumor.

Similar to other genomic studies, the difficulty of interpreting phenotypic associations lies in the demonstration of the mechanistic basis of the findings. Previous studies of pQTLs for VEGF-A have not provided the mechanistic basis of the associations^16–18^ and performing functional experiments can identify new regulators of *VEGFA* expression. The NF-AT1 transcription factor was predicted by HaploReg to bind to the region where rs7767396 is located, and variation of rs7767396 alters the binding of the NF-AT1 motif. A complementary analysis using the PBM data from the cis-BP database has confirmed this prediction, providing strong evidence in support of the role of rs7767396 with respect to the NF-AT1 transcription factor. More importantly, the PBM data have demonstrated that the binding efficiency of NF-AT1 is dramatically reduced by the presence of the G allele of rs7767396 as compared to the A allele (Fig. 4). VEGF-A triggers activation of NF-AT1 in vascular endothelial cells^26–28^, and NF-AT1 inhibition has been shown to block expression of certain VEGF-A-induced genes^29^, although it is currently unknown whether NF-AT1 can act as a distal regulator of VEGF-A expression^28,30,31^. Our study results support the mechanism that, because of reduced transcriptional activation of *VEGFA* through reduced binding of NF-AT1, subjects with the G allele of rs7767396 have significantly reduced VEGF-A plasma levels in the circulation.

In our discovery cohort, the P-value for the SNP-protein pair (rs7767396-VEGF-A) (P-value = 5.8e–09) did not surpass the strict Bonferroni-corrected threshold of statistical significance (P-value < 3.3e–9). In our validation cohort, however, the corresponding P-value (5.2e–5) surpassed the equivalent threshold (P-value < 1.7e–2). The evidence in support for this pQTL is further bolstered by the strong associations reported in the Debette *et al*.^21^ and Sun *et al*.^23^ papers.

In addition to the results discussed above, we also provide genome-wide associations of SNPs regarding an additional 28 proteins (Supplementary File 1) to guide future research on these associations. Availability of results of SNP-protein pair associations will allow replication of hits with less statistical significance by other investigators who will test such associations prospectively.

In summary, we have identified a heritable common *cis* variant that regulates circulating VEGF-A levels in plasma of patients with advanced adenocarcinoma of the pancreas or colon. This variant has been reported to regulate circulating VEGF-A levels in a large cardiology cohort and a large COPD case-control cohort, but no data have been reported in a cancer population. This study is the first to identify a common variant near the *VEGFA* gene regulating the plasma levels of the protein in cancer patients. It is also the first study elucidating how this variant changes VEGF-A plasma levels, by altering the binding of the NF-AT1 transcription factor to a regulatory element about 170 Kb distant from *VEGFA*. Similar studies in tumor types outside of the gastrointestinal tract are warranted.

## Methods

### Clinical trials and patients

CALGB 80303 was the discovery cohort study. CALGB 80303 was a double-blind, placebo-controlled, randomized phase III study of bevacizumab in combination with gemcitabine in treatment-naïve advanced pancreatic adenocarcinoma patients. Patient eligibility, characteristics, stratifications, response evaluation, and treatments have been previously described^32^. The characteristics of the genetically estimated European patients for whom genotype data and plasma protein levels were available are described in Table 2.

**Table 2:** Baseline clinical characteristics of patients in CALGB 80303 and 80203.

		CALGB 80303	CALGB 80203
Number of patients		216	114
Sex	Male	118	68
	Female	98	46
Age (years)	Median (range)	64 (35–84)	63 (23–83)
Extent of disease	Metastatic	183	110
	Locally Advanced	33	3
	Unknown	0	1
Performance status	ECOG 0 or 1	195	114
	2	21	

Patients in the primary analysis population, i.e., having both genotype and circulating protein data, are summarized. Summaries are restricted to CALGB 80303 patients estimated to be genetically European and CALGB 80203 patients who self-reported as white and non-Hispanic. Patients with ECOG status 2 were excluded from registration to the CALGB 80203 clinical study per protocol.

CALGB 80203 was the validation cohort study. CALGB 80203 was a double-blind, randomized study in patients with previously untreated, advanced or metastatic colorectal cancer. Patients were treated with 5-fluorouracil/oxaliplatin or 5-fluorouracil/irinotecan with or without cetuximab. Additional design details of the CALGB 80203 study were previously described^33,34^. The characteristics of the self-reported white and non-Hispanic patients, for whom genotype data and plasma protein levels were available, are described in Table 2.

This research was approved by the Institutional Review Board of each participating institution. Each participant signed an IRB-approved, protocol-specific informed consent. All experiments were performed in accordance with relevant guidelines and regulations for the clinical, SNP and protein marker analysis; work was performed under the auspices of protocol number Pro00018430.

### Genotyping

In CALGB 80303, germline genome-wide genotype data of 484,523 directly interrogated SNPs were collected from 294 genetically estimated European patients using the Illumina 550 K platform^35^. Quality control of the genotyping has been previously described^35^. Among these 294 patients, 216 had consented samples available for measurement of plasma proteins of angiogenesis (Fig. 1). The genotyping was conducted at the Center for Genomic Medicine at the RIKEN Institute.

In CALGB 80203, three SNPs (rs2284284, rs7504372, and rs7767396) were genotyped from germline DNA in 117 self-reported white, non-Hispanic patients (Fig. 2), using TaqMan SNP genotyping assays (Applied Biosystems, Foster City, CA). Sanger-based DNA sequencing (Mammalian Genotyping Core, University of North Carolina-Chapel Hill) was used to validate representative samples and determine thresholds for allelic discrimination. PCR primers used for amplification of genomic DNA prior to sequencing are listed in Supplementary Table 1.

### Measurement of plasma proteins of angiogenesis

In CALGB 80303 and 80203, 31 proteins were measured (Table 3) using the SearchLight multiplex platform (Aushon BioSystems, Inc., Billerica, MA). The VEGF-A assay detects the VEGF-A_165_ isoform (the predominant isoform among the circulating ones) preferentially, but it is not considered isoform-specific^36,37^. Plasma samples were collected before treatment and stored at −80 °C until analysis. The frozen samples were thawed on ice, centrifuged at 20,000 × g for 5 min to remove any precipitate, and appropriately diluted before placement onto multiplex plates. Plasma samples had no more than two freeze-thaw cycles and all assays were performed in duplicate. The number of patients for whom proteins were measured is described in Table 2. Detailed information about the distributions of these protein markers and their relationship to clinical outcomes in CALGB 80303^36^ and 80203^37^ have been published. The analyses were carried out at the Phase I Biomarker Laboratory at Duke University Medical Center.

**Table 3:** Proteins measured in plasma of patients enrolled in CALGB 80303 and 80203.

Soluble Angiogenic Factors		Matrix-Derived Angiogenic Factors	Markers of Vascular Activation and Inflammation	
ANG-2	PEDF	Osteopontin	CRP	PAI-1 Active
bFGF	PlGF	TGFβ1	Gro-α	PAI-1 Total
HGF	VEGF-A	TGFβ2	ICAM-1	P-selectin
IGF-1	VEGF-C	sTGFβRIII	IL-6	SDF1
IGFBP1	VEGF-D	TSP2	IL-8	VCAM-1
IGFBP3	sVEGFR1		MCP-1	
PDGF-AA	sVEGFR2			
PDGF-BB				

ANG-2 = angiopoietin-2; bFGF = basic fibroblast growth factor; HGF = hepatocyte growth factor; IGF-1 = insulin-like growth factor-1; IGFBP = insulin-like growth factor-binding protein; PDGF = platelet-derived growth factor; PEDF = pigment epithelium-derived factor; PlGF = placental growth factor; VEGF = vascular endothelial growth factor; sVEGFR = soluble vascular endothelial growth factor receptor; TGFβ = transforming growth factor beta; sTGFβRIII = soluble transforming growth factor beta receptor type III; TSP = thrombospondin; CRP = c-reactive protein; PAI-1 = plasminogen activator inhibitor-1; Gro-α = growth regulated oncogene-alpha; ICAM-1 = intercellular adhesion molecule 1; IL = interleukin; MCP-1 = macrophage chemoattractant protein-1; SDF1 = stromal cell-derived factor-1; VCAM-1 = vascular cell adhesion molecule 1.

### Statistics

A two-stage approach was used to detect genetic associations with circulating protein levels. CALGB 80303 served as the discovery cohort, and selected genetic variants were then genotyped in CALGB 80203, which served as the validation cohort. In the discovery cohort, the genome-wide analyses used an additive genetic model, and only autosomal SNPs were evaluated.

The Jonckheere-Terpstra^38,39^ test was used to test the association between each SNP and protein level. The Jonckheere-Terpstra test was selected for its desirable qualities of being rank-based, making it robust to outliers, and distribution-free, meaning its validity would not depend on the normality and homogeneity of the variances. Since the test is powered for ordered alternatives, it is applicable to pQTL-type analyses (analogous to the Cochran-Armitage test for binary outcomes). The variance of the Jonckheere-Terpstra test was approximated using expression 6.19 in Hollander, *et al*.^40^. For each protein level, the distribution of the marginal P-values across all SNPs was assessed empirically using QQ and Manhattan plots. A robust linear regression rank-based approach^41,42^ was used to estimate the proportion of variance of the phenotype explained by SNPs. For this analysis, the protein level was log base 2 transformed. This regression approach was also used to investigate the relationship between protein level and genotype, accounting for sex and age (log_10_ transformed) as baseline covariates, in both CALGB 80303 and 80203. The concordance between plasma protein levels and mRNA levels in tumors was assessed using Kendall’s test of concordance^43^.

All statistical analyses were conducted using two-sided alternative hypotheses and were restricted to genetic Europeans for CALGB 80303 or self-reported white, non-Hispanic patients for CALGB 80203. For validation of SNP-protein pairs, we specified that the direction of effect with respect to the minor frequency allele must be the same in both studies and used a marginal two-sided P-value cutoff of 0.05/k, where k denotes the number of pairs chosen for validation based on the results from the discovery cohort. Additional statistical details are reported in the Supplementary Information section.

### Protein-DNA binding microarray data

After using HaploReg v4.1^44^ to identify putative transcription factors binding in the region of rs7767396, we tested the specificity of binding used PBM data from the cis-BP database^45^. PBM experiments provide, in addition to DNA motif models, quantitative measurements of *in vitro* protein-DNA binding specificity for all possible 8-bp DNA sequences. Such measurements can be downloaded from cis-BP in the form of 8-mer binding enrichment scores, which vary from −0.5 to 0.5, with values above 0.37 corresponding to 8-mers specifically bound by the protein (false discovery rate, FDR, 0.001)^46^.

### Quantification of mRNA VEGF-A isoforms in primary tissues from CALGB 80203 patients

These methods are reported in the Supplementary Information section.

## Electronic supplementary material

Supplementary Information

Supplementary File 2

## Data Availability

The GWAS data from the CALGB 80303 discovery cohort are available from the database of Genotypes and Phenotypes (dbGaP) through Study Accession: phs000250.v1.p1. The baseline characteristic data from CALGB 80303 and CALGB 80203, the candidate SNP and *VEGFA* isoform data from CALGB 80203, the protein data from CALGB 80203 and 80303, and the SNP annotation for the top hits of the Jonckheere-Terpstra test for the 31 proteins are provided as an Excel workbook (Supplemental File 2). Code and scripts to replicate the analyses presented in this paper are included as appendices here and are also available through a source code repository (https://bitbucket.org/calgbgwas/calgb80303-vegfa-nfat1-paper).

## References

[1] Ferrara N, Gerber HP, LeCouter J (2003). The biology of VEGF and its receptors. Nat Med.

[2] Folkman J (2002). Role of angiogenesis in tumor growth and metastasis. Semin Oncol.

[3] Veikkola T, Karkkainen M, Claesson-Welsh L, Alitalo K (2000). Regulation of angiogenesis via vascular endothelial growth factor receptors. Cancer Res.

[4] Iqbal S, Lenz HJ (2004). Angiogenesis inhibitors in the treatment of colorectal cancer. Semin Oncol.

[5] Ramjiawan RR, Griffioen AW, Duda DG (2017). Anti-angiogenesis for cancer revisited: Is there a role for combinations with immunotherapy?. Angiogenesis.

[6] Wang Z (2015). Broad targeting of angiogenesis for cancer prevention and therapy. Semin Cancer Biol.

[7] Takahashi Y, Kitadai Y, Bucana CD, Cleary KR, Ellis LM (1995). Expression of vascular endothelial growth factor and its receptor, KDR, correlates with vascularity, metastasis, and proliferation of human colon cancer. Cancer Res.

[8] Amaya H (1997). Association of vascular endothelial growth factor expression with tumor angiogenesis, survival and thymidine phosphorylase/platelet-derived endothelial cell growth factor expression in human colorectal cancer. Cancer Lett.

[9] Fujisaki K, Mitsuyama K, Toyonaga A, Matsuo K, Tanikawa K (1998). Circulating vascular endothelial growth factor in patients with colorectal cancer. Am J Gastroenterol.

[10] Karayiannakis AJ (2002). Circulating VEGF levels in the serum of gastric cancer patients: correlation with pathological variables, patient survival, and tumor surgery. Ann Surg.

[11] Jelkmann W (2001). Pitfalls in the measurement of circulating vascular endothelial growth factor. Clin Chem.

[12] Hatch AJ, Clarke JM, Nixon AB, Hurwitz HI (2015). Identifying Blood-Based Protein Biomarkers for Antiangiogenic Agents in the Clinic: A Decade of Progress. Cancer J.

[13] Lissoni P (2003). Changes in circulating VEGF levels in relation to clinical response during chemotherapy for metastatic cancer. Int J Biol Markers.

[14] Berrahmoune H (2007). Heritability for plasma VEGF concentration in the Stanislas family study. Ann Hum Genet.

[15] Lieb W (2009). Vascular endothelial growth factor, its soluble receptor, and hepatocyte growth factor: clinical and genetic correlates and association with vascular function. Eur Heart J.

[16] Pantsulaia I, Trofimov S, Kobyliansky E, Livshits G (2004). Heritability of circulating growth factors involved in the angiogenesis in healthy human population. Cytokine.

[17] Niers TM, Richel DJ, Meijers JC, Schlingemann RO (2011). Vascular endothelial growth factor in the circulation in cancer patients may not be a relevant biomarker. PLoS One.

[18] Nakamura I (2013). Serum levels of vascular endothelial growth factor are increased and correlate with malnutrition, immunosuppression involving MDSCs and systemic inflammation in patients with cancer of the digestive system. Oncol Lett.

[19] Begg C (1996). Improving the quality of reporting of randomized controlled trials. The CONSORT statement. JAMA.

[20] National Center for Biotechnology Information, U.S. National Library of Medicine. *Database of Single Nucleotide Polymorphisms (dbSNP); dbSNP Build ID*: **148**, http://www.ncbi.nlm.nih.gov/SNP/ (2017).

[21] Debette S (2011). Identification of cis- and trans-acting genetic variants explaining up to half the variation in circulating vascular endothelial growth factor levels. Circ Res.

[22] Choi Seung Hoan, Ruggiero Daniela, Sorice Rossella, Song Ci, Nutile Teresa, Vernon Smith Albert, Concas Maria Pina, Traglia Michela, Barbieri Caterina, Ndiaye Ndeye Coumba, Stathopoulou Maria G., Lagou Vasiliki, Maestrale Giovanni Battista, Sala Cinzia, Debette Stephanie, Kovacs Peter, Lind Lars, Lamont John, Fitzgerald Peter, Tönjes Anke, Gudnason Vilmundur, Toniolo Daniela, Pirastu Mario, Bellenguez Celine, Vasan Ramachandran S., Ingelsson Erik, Leutenegger Anne-Louise, Johnson Andrew D., DeStefano Anita L., Visvikis-Siest Sophie, Seshadri Sudha, Ciullo Marina (2016). Six Novel Loci Associated with Circulating VEGF Levels Identified by a Meta-analysis of Genome-Wide Association Studies. PLOS Genetics.

[23] Sun W (2016). Common Genetic Polymorphisms Influence Blood Biomarker Measurements in COPD. PLoS Genet.

[24] Hegde PS (2013). Predictive impact of circulating vascular endothelial growth factor in four phase III trials evaluating bevacizumab. Clin Cancer Res.

[25] Aoyagi Y, Iinuma H, Horiuchi A, Shimada R, Watanabe T (2010). Association of plasma VEGF-A, soluble VEGFR-1 and VEGFR-2 levels and clinical response and survival in advanced colorectal cancer patients receiving bevacizumab with modified FOLFOX6. Oncol Lett.

[26] Armesilla AL (1999). Vascular endothelial growth factor activates nuclear factor of activated T cells in human endothelial cells: a role for tissue factor gene expression. Mol Cell Biol.

[27] Hernandez GL (2001). Selective inhibition of vascular endothelial growth factor-mediated angiogenesis by cyclosporin A: roles of the nuclear factor of activated T cells and cyclooxygenase 2. J Exp Med.

[28] Muller MR, Rao A (2010). NFAT, immunity and cancer: a transcription factor comes of age. Nat Rev Immunol.

[29] Voron T (2015). VEGF-A modulates expression of inhibitory checkpoints on CD8 + T cells in tumors. J Exp Med.

[30] Suehiro J (2014). Genome-wide approaches reveal functional vascular endothelial growth factor (VEGF)-inducible nuclear factor of activated T cells (NFAT) c1 binding to angiogenesis-related genes in the endothelium. J Biol Chem.

[31] Chen K (2015). ZBRK1, a novel tumor suppressor, activates VHL gene transcription through formation of a complex with VHL and p300 in renal cancer. Oncotarget.

[32] Kindler HL (2010). Gemcitabine plus bevacizumab compared with gemcitabine plus placebo in patients with advanced pancreatic cancer: phase III trial of the Cancer and Leukemia Group B (CALGB 80303). J Clin Oncol.

[33] Venook A (2006). Phase III study of irinotecan/5FU/LV (FOLFIRI) or oxaliplatin/5FU/LV (FOLFOX) ± cetuximab for patients (pts) with untreated metastatic adenocarcinoma of the colon or rectum (MCRC): CALGB 80203 preliminary results. J Clin Onc.

[34] Cushman SM (2015). Gene expression markers of efficacy and resistance to cetuximab treatment in metastatic colorectal cancer: results from CALGB 80203 (Alliance). Clin Cancer Res.

[35] Innocenti F (2012). A genome-wide association study of overall survival in pancreatic cancer patients treated with gemcitabine in CALGB 80303. Clin Cancer Res.

[36] Nixon AB (2013). Prognostic and predictive blood-based biomarkers in patients with advanced pancreatic cancer: results from CALGB80303 (Alliance). Clin Cancer Res.

[37] Hatch AJ (2016). Blood-based markers of efficacy and resistance to cetuximab treatment in metastatic colorectal cancer: results from CALGB 80203 (Alliance). Cancer Med.

[38] Terpstra TJ (1952). The Asymptotic Normality and Consistency of Kendall’s Test Against Trend, When Ties are Present in One Ranking. Indagat Math.

[39] Jonckheere AR (1954). A Distribution-Free Kappa-Sample Test against Ordered Alternatives. Biometrika.

[40] Hollander, M. & Wolfe, D. A. *Nonparametric Statistical Methods* (2nd ed), (John Wiley & Sons 1999).

[41] Jaeckel LA (1972). Estimating regression coefficients by minimizing the dispersion of residuals. Ann Math Stat.

[42] Jureckova J (1971). Nonparametric estimate of regression coefficients. Ann Math Stat.

[43] Kendall M (1938). A New Measure of Rank Correlation. Biometrika.

[44] Ward LD, Kellis M (2016). HaploRegv4: systematic mining of putative causal variants, cell types, regulators and target genes for human complex traits and disease. Nucleic Acids Res.

[45] Weirauch MT (2014). Determination and inference of eukaryotic transcription factor sequence specificity. Cell.

[46] Badis G (2009). Diversity and complexity in DNA recognition by transcription factors. Science.

