# Supplementary material for: Genetic variation determines VEGF-A plasma levels in cancer patients
Source: Sci Rep. 2018 Nov 5;8:16332. doi: 10.1038/s41598-018-34506-4 (PMC6218528; doi:10.1038/s41598-018-34506-4)
Supplement: Supplementary file 1 — Supplementary Information [file 41598_2018_34506_MOESM1_ESM.pdf]

Genetic variation determines VEGF-A plasma levels in cancer patients

Federico Innocenti[1]*, Chen Jiang[2], Alexander B. Sibley[2], Amy S. Etheridge[1], Ace J. Hatch[2], Stefanie Denning[1], Donna Niedzwiecki[2], Ivo D. Shterev[3], Jiaxing Lin[4], Yoichi Furukawa[5], Michiaki Kubo[6], Hedy L. Kindler[7], J. Todd Auman[8], Alan P. Venook[9], Herbert I. Hurwitz[2], Howard L. McLeod[10], Mark J. Ratain[7], Raluca Gordan[4], Andrew B. Nixon[2], Kouros Owzar[2,4]

Supplementary Information

Measurement of *VEGFA* mRNA levels by quantitative real-time PCR (qRT-PCR) in primary tumors from CALGB 80203

For the CALGB 80203 samples, RNA was isolated and analyzed as previously described [1]. After pathologist review, slides were manually macro-dissected to isolate regions containing >70% tumor. RNA was isolated from six 10-µm sections using the Ambion RecoverAll Total Nucleic Isolation kit according to the manufacturer's protocol (Ambion-Life Technologies, Austin, TX, USA). The High Capacity cDNA Reverse Transcription kit (Applied Biosystems-Life Technologies, Foster City, CA, USA) was used to reverse-transcribe RNA (200 ng) from each sample. Taqman qRT-PCR was performed using the StepOne Real-Time PCR System (Applied Biosystems-Life Technologies, Foster City, CA, USA). Levels of total VEGF-A and β-actin were quantified using the TaqMan assays Hs00900055_m1 and Hs00357333_g1, respectively (Applied Biosystems-Life Technologies, Foster City, CA, USA). Four *VEGFA* mRNA isoforms (121, 145, 165 and 189), were quantified using custom

TaqMan assays consisting of the components shown in Supplementary Table 2. Relative *VEGFA* isoform mRNA expression was normalized to β-actin mRNA and expressed as $\log2(2^{-(CycleX-Cycle\beta\text{-}actin)}) = -(CycleX-Cycle\beta\text{-}actin)$, where Cycle is the threshold cycle.

Tumor tissue levels of *VEGFA* mRNA isoforms were measured for 96 CALGB 80203 patients (Figure 2). However, only 65 patients had both VEGF-A plasma and *VEGFA* mRNA available for analysis. Fifty-one self-reported white, non-Hispanic patients were typed for rs7767396 and had *VEGFA* mRNA levels from tumor. No primary tumor tissue was available from CALGB 80303. The analyses were carried out at the Phase I Biomarker Laboratory at Duke University Medical Center.

Additional statistical considerations and study design

The genome-wide level of significance for P-values for the CALGB 80303 discovery cohort was determined by comparing each marginal P-value to the Bonferroni threshold of 0.05/(31*484,523) = 3.3e-9 to account for testing 484,523 SNPs that passed the genome-wide GWAS quality control battery over 31 proteins.

The results of the Jonckheere-Terpstra (JT) test were compared with those of the commonly used linear regression model (LM) of quantile-normalized data for the three SNP-protein pairs tested in CALGB 80303. To control for population structure, the VEGF-A-rs7767396 pair was reanalyzed in CALGB 80303 using the robust rank-based linear regression framework outlined in the paper, adjusted for the first three principal components.

All statistical analyses were carried out using the R [2] statistical environment. The analyses were carried out by Alliance statisticians. The GenABEL package [3] was used

to calculate P-values for the exact test for departure from Hardy-Weinberg Equilibrium in the discovery cohort. The fastJT package [4] was used to conduct genome-wide inference on the basis of the Jockheere-Terpstra test and the Rfit [5] extension package was used for estimating the proportion of genetically determined variance in protein expression and for the multivariable analyses using baseline covariates. The genetics package [6] was used to calculate P-values for the exact test for departure from Hardy-Weinberg in the validation cohort. The knitr extension package was used to generate dynamic analysis reports [7].

Bioinformatics analyses

The genomic locations of the SNPs and genes are from build hg19 of the human genome. The Locus Zoom software (version 1.3) was used to assess the extent of the signals relative to the genomic position and linkage disequilibrium (LD) within a region (e.g., gene or neighborhood around a SNP). The 1000 Genomes Pilot 1 EUR panel data (June 2010 release), based on human genome build hg19, were used as the reference data. Putative estimates of LD among SNPs were obtained from the 1000 Genomes Project 1 CEU panel data through the SNAP web interface [8,9]. The HaploReg v4 [10] resource was used to explore the putative functional mechanism of non-coding variants with respect to transcription factors.

Additional Results

Supplementary Table 3 summarizes the comparison between the Jonckheere-Terpstra (JT) and the linear model (LM) for 3 proteins. In addition, within the framework of a multivariable regression model, with PC1, PC2 and PC3 as the covariates, the

association between rs7767396 and VEGF-A levels remained strong (P-value = 9.7e-10).

References

1. Cushman, S. M. *et al.* Gene expression markers of efficacy and resistance to cetuximab treatment in metastatic colorectal cancer: results from CALGB 80203 (Alliance). *Clin. Cancer Res.* 21, 1078-1086 (2015).
2. R Core Team (2016). *R: A Language and Environment for Statistical Computing.* R Foundation for Statistical Computing. Vienna, Austria. URL http://www.R-project.org
3. GenABEL (2013): genome-wide SNP association analysis. R package version 1.8-0. URL https://CRAN.R-project.org/package=GenABEL
4. Lin, J., Sibley, A., Shterev, I. & Owzar, K. (2017). fastJT: Efficient Jonckheere-Terpstra Test Statistics for Robust Machine Learning and Genome-Wide Association Studies. R package version 1.0. URL https://CRAN.R-project.org/package=fastJT
5. Kloke, J. D. & Mckean, J. W. Rfit: Rank-based estimation for linear models. *The R Journal* 4, 57-64 (2012).
6. Warnes, G., Gorjanc, G., Leisch, F. & Man, M. (2013). *genetics: Population Genetics. R package version 1.3.8.1.* URL https://CRAN.R-project.org/package=genetics
7. Xie, Y. *Dynamic Documents with R and Knitr*, 2nd edition. (CRC Press, 2015).
8. Pruim, R. J. *et al.* LocusZoom: regional visualization of genome-wide association scan results. *Bioinformatics* 26, 2336-2337 (2010).
9. Johnson, A. D. *et al.* SNAP: a web-based tool for identification and annotation of proxy SNPs using HapMap. *Bioinformatics* 24, 2938-2939 (2008).

10. Ward, L. D. & Kellis, M. HaploReg v4: systematic mining of putative causal variants, cell types, regulators and target genes for human complex traits and disease. *Nucleic Acids Res.* 44, D877-881 (2016).

Supplementary Figure 1. Quantile-quantile plots of SNP associations with plasma levels of VEGF-A, VEGF-C, and MCP1 in CALGB 80303. –log10(P-values) from the Jonckheere-Terpstra test for ordered alternatives for 484,523 SNPs in 216 patients are plotted versus random values from a uniform distribution over the interval [0,1].

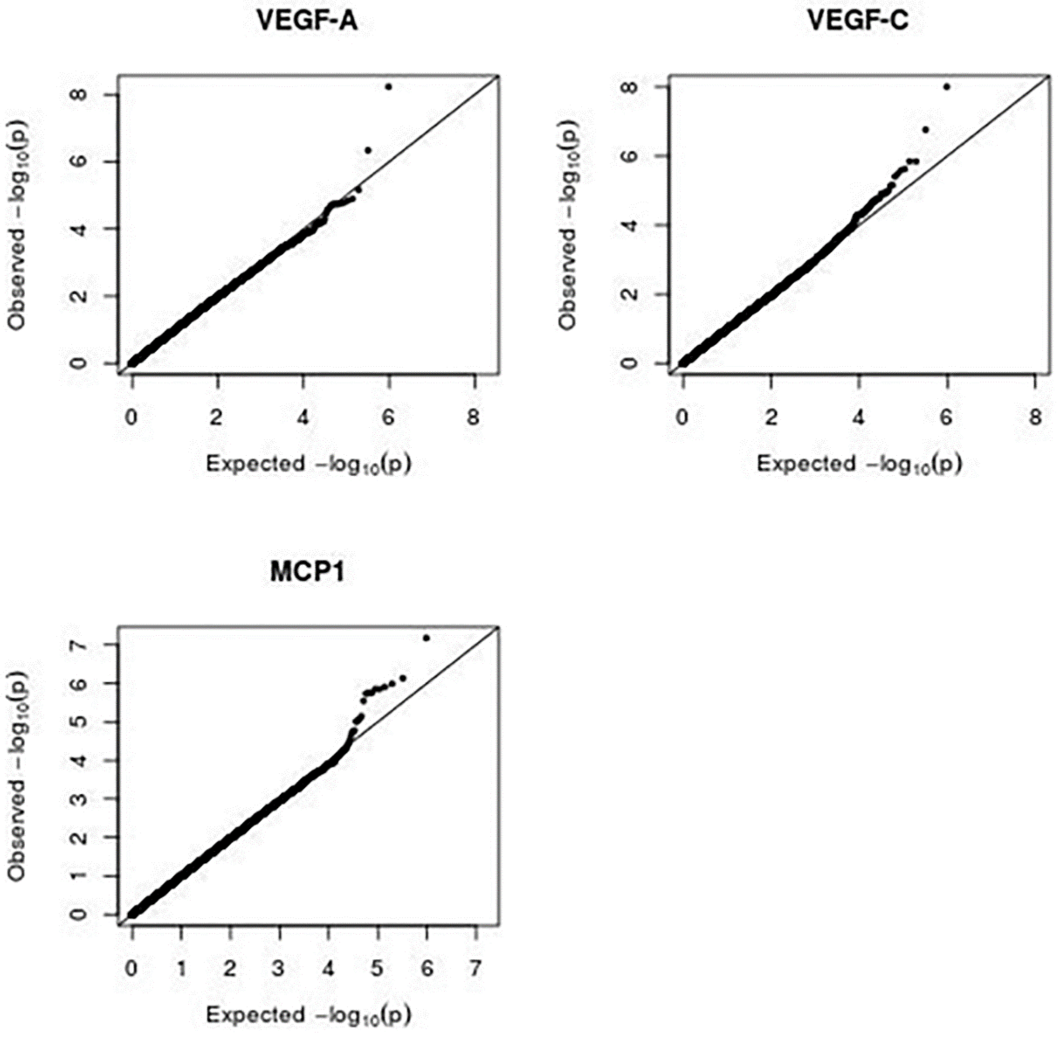

Supplementary Figure 2. Manhattan plots of SNP associations with plasma levels of VEGF-A, VEGF-C, and MCP1 in CALGB 80303. P-values from the Jonckheere-Terpstra test for ordered alternatives are plotted for 484,523 SNPs in 216 patients. Analyzed SNPs are ordered on the x-axis by chromosomal position. The y-axis corresponds to the –$\log_{10}$(P-value).

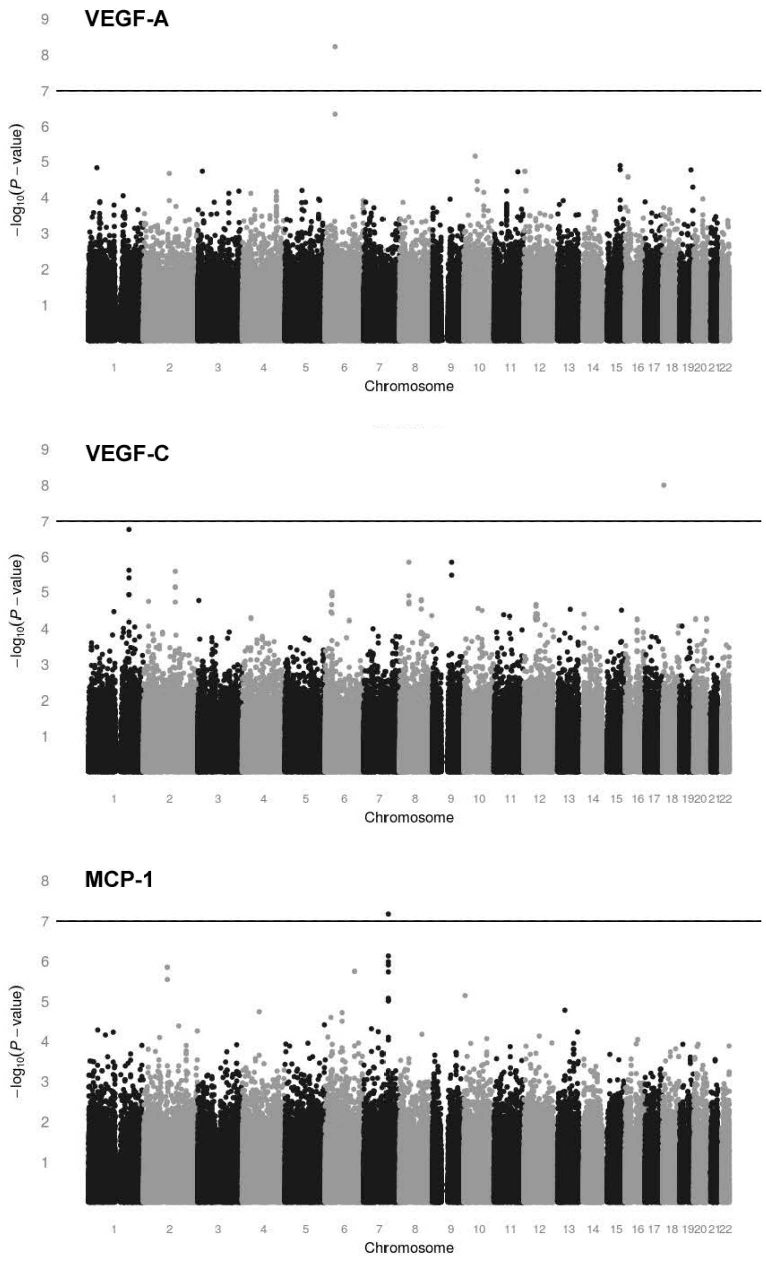

Supplementary Figure 3. Scatter plots of tumor VEGF-A isoform levels vs. plasma levels of VEGF-A in CALGB 80203. Kendall's Tau was used to test for association between levels of circulating VEGF-A in plasma and *VEFGA* mRNA isoform levels in tumors. Data were available for isoforms 121, 145, 165, 189, and total *VEFGA* for 63, 33, 64, 63, and 65 patients, respectively.

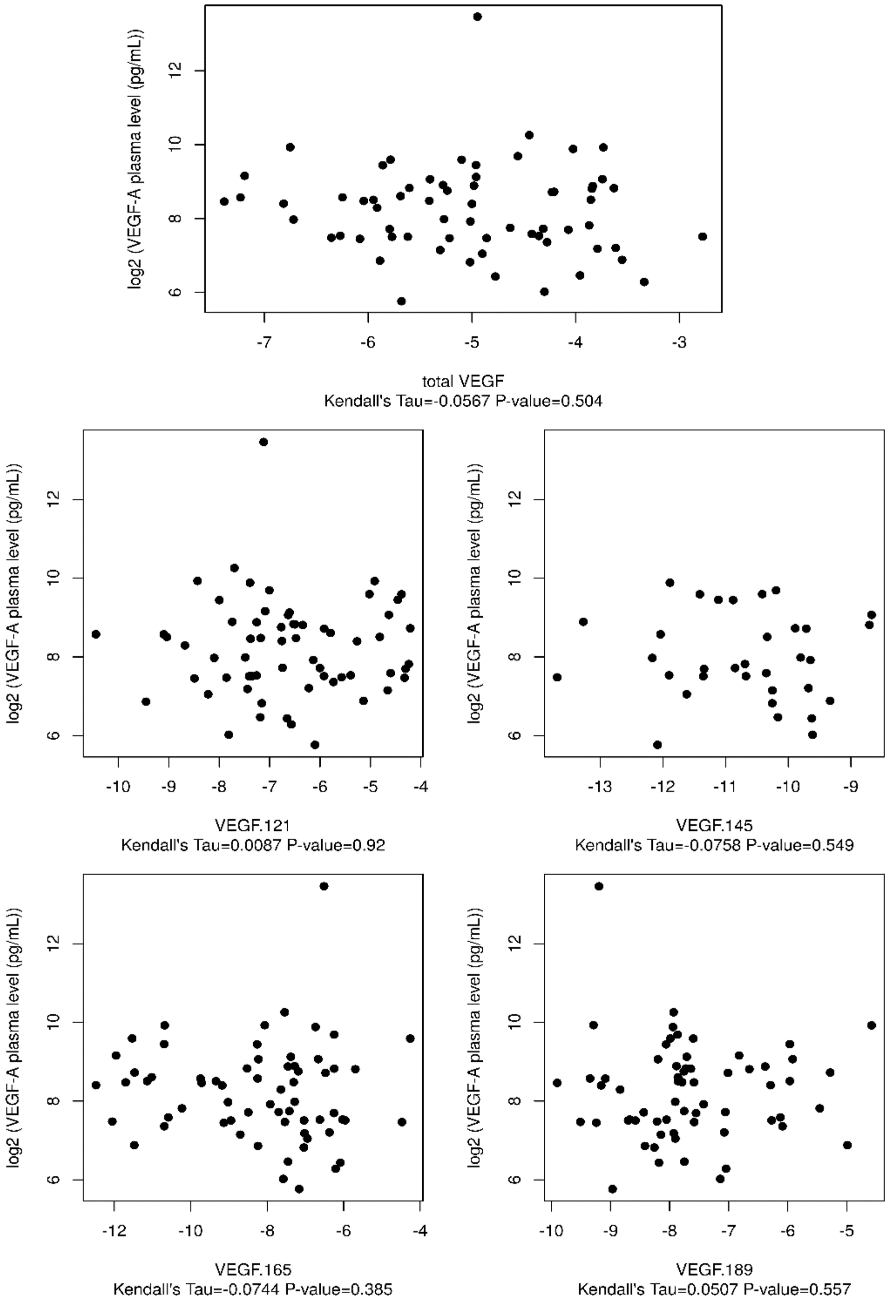

Supplementary Table 1. Primers used to amplify genomic DNA regions for Sanger sequencing. Following TaqMan SNP genotyping of rs2284284, rs7504372, and rs7767396, Sanger sequencing is used to validate representative samples and determine thresholds for allelic discrimination. PCR primers are designed to amplify genomic DNA regions used for sequencing.

SNP in fragment	Forward primer	Reverse primer	Genomic region
rs2284284	CCAGAAATAGCTGCAGAAGG	CTGAAAGTCCGCTCAGACG	chr7: 107892970-107893470
rs7504372	GCTTCTGCCACCAGTAAAGG	GGTTATTTTGAGGATGTCGAAGG	chr18: 5931835-5932335
rs7767396	CCTTTAAGGCTCATTACACTTGG	GGAAGCACTCATCTTCATCAGG	chr6: 43926876-439277267

Supplementary Table 2. Primers used to measure four *VEGFA* mRNA isoforms from formalin fixed, paraffin-embedded tumor samples from CALGB 80203 patients.

Assay target	Forward primer	Reverse primer	Probe
VEGFA121	ACAACAAATGTGAATGCAGACCAAA	CTGAGGGAGGCTCCTTCCT	CAAGAAAAATGTGACAAGCCG
VEGFA145	GCAAGAAATCCCGGTATAAGTCC	GCTCACCGCCTCGGC	GGAGCGTATGTGAC
VEGFA165	ACAACAAATGTGAATGCAGACCAAA	GCTTTCTCCGCTCTGAGCAA	CCACAGGGATTTTCT
VEGFA189	GCGCAAGAAATCCCGGTATAAGT	GCTTTCTCCGCTCTGAGCAA	CCTGGAGCGTTCCCTG

Supplementary Table 3. Comparison between the Jonckheere-Terpstra (JT) and linear model (LM) analyses for three SNP-protein pairs.

**Protein**	**SNP**	**P-value (JT)**	***P-value (LM)***
MCP1	rs2284284	6.7e-08	*2.6e-04*
VEGF-C	rs7504372	9.8e-09	*3.5e-06*
VEGF-A	rs7767396	5.8e-09	*4.2e-07*

Supplementary File 1. Top 50 most statistically significant associations for each of the 31 proteins levels measured in CALGB 80303. We either report the gene in which the SNP is located (overlapping symbols are concatenated with "&"), or the flanking genes (within 1e06bp) when the SNP is intergenic.

	FGFb	VEGF.C	PlGF	GROa	PDGF.AA	SDF.1b	VEGF.D	IL.6
1	rs508362<br>2.2e-06<br>RREB1	rs7504372<br>9.8e-09<br>TMEM200C<br>L3MBTL4	rs1673082<br>1.3e-05<br>ZNF850	rs2729747<br>1.5e-06<br>PAK1	rs9972517<br>1.8e-07<br>TMC3<br>MEX3B	rs752388<br>1.6e-07<br>PENK<br>LINC00968	rs9853624<br>8.7e-06<br>LARS2<br>LIMD1	rs10510941<br>3.8e-06<br>MAGI1
2	rs200853<br>6.1e-06<br>RPP40<br>PPP1R3G	rs12031722<br>1.7e-07<br>RGL1	rs522636<br>1.4e-05<br>GABRG1<br>GABRA2	rs2725830<br>1.5e-06<br>PAK1	rs238117<br>1.3e-06<br>TGIF1<br>DLGAP1	rs10504202<br>3.7e-07<br>SDR16C5<br>PENK	rs4977427<br>1.1e-05<br>MLLT3	rs12537330<br>7.1e-06<br>NXPH1<br>PER4
3	rs4327622<br>1.2e-05<br>NA<br>MIR3660	rs4877822<br>1.4e-06<br>RMI1<br>SLC28A3	rs534787<br>1.4e-05<br>GABRG1<br>GABRA2	rs2729760<br>1.7e-06<br>PAK1	rs6601604<br>3.8e-06<br>GATA4	rs7007715<br>3.9e-07<br>SDR16C5<br>PENK	rs12377027<br>1.8e-05<br>MLLT3	rs10242846<br>7.5e-06<br>AHR<br>SNX13
4	rs7720113<br>1.2e-05<br>NA<br>MIR3660	rs2599662<br>1.4e-06<br>ZMAT4	rs2162296<br>1.4e-05<br>ZNF382	rs2852394<br>3.2e-06<br>PAK1	rs7908340<br>6.2e-06<br>FZD8<br>NA	rs17828094<br>5.2e-07<br>SDR16C5<br>PENK	rs10964620<br>1.9e-05<br>MLLT3	rs4917978<br>1.1e-05<br>SUFU
5	rs6884617<br>1.2e-05<br>SH3TC2	rs3814329<br>2.4e-06<br>RGL1	rs572227<br>1.6e-05<br>GABRG1<br>GABRA2	rs9386913<br>3.9e-06<br>SLC22A16<br>CDK19	rs4482239<br>6.6e-06<br>TMC3<br>MEX3B	rs2670049<br>7.8e-07<br>PENK<br>LINC00968	rs1979923<br>2.3e-05<br>SLIT3	rs10786696<br>1.1e-05<br>SUFU
6	rs1246996<br>1.4e-05<br>RPP40<br>PPP1R3G	rs972485<br>2.6e-06<br>LRP1B	rs534459<br>1.7e-05<br>GABRA2	rs11153238<br>7.5e-06<br>SLC22A16<br>CDK19	rs6544272<br>6.8e-06<br>NA<br>KLHL29	rs13256083<br>8.5e-07<br>SDR16C5<br>PENK	rs6448932<br>2.4e-05<br>NA<br>RAB28	rs2839947<br>1.1e-05<br>MTHFD1L
7	rs1511168<br>1.5e-05<br>MIR3148<br>MIR548O2	rs1932660<br>3.3e-06<br>RMI1<br>SLC28A3	rs7115956<br>1.7e-05<br>C11orf87<br>ZC3H12C	rs11237146<br>1.0e-05<br>GDPD4	rs4778930<br>7.0e-06<br>TMC3<br>MEX3B	rs2582394<br>1.9e-06<br>PENK<br>LINC00968	rs7552722<br>2.5e-05<br>NGF<br>VANGL1	rs9557595<br>1.3e-05<br>NALCN
8	rs12676771<br>1.8e-05<br>TRIB1<br>LINC00861	rs17504460<br>3.9e-06<br>COLGALT2	rs4053616<br>2.6e-05<br>BACH2	rs1793467<br>1.1e-05<br>GDPD4	rs2001427<br>8.9e-06<br>NA<br>KLHL29	rs13325144<br>3.8e-06<br>COPB2<br>RBP2	rs1548586<br>2.9e-05<br>MAP3K9<br>PCNX1	rs11718781<br>1.3e-05<br>PROK2<br>LINC00870
9	rs2832616<br>1.9e-05<br>GRIK1<br>CLDN17	rs974973<br>7.0e-06<br>LRP1B	rs2850094<br>2.7e-05<br>CLDN14<br>SIM2	rs4925088<br>1.1e-05<br>SPECC1	rs6133566<br>1.4e-05<br>PLCB1	rs4644241<br>9.8e-06<br>SDR16C5<br>PENK	rs1204371<br>3.6e-05<br>ATXN7L1	rs3024896<br>1.9e-05<br>STAT4
10	rs1943950<br>1.9e-05<br>GRIK1<br>CLDN17	rs4954923<br>7.3e-06<br>LRP1B	rs10880346<br>3.1e-05<br>PRICKLE1<br>ADAMTS20	rs7160403<br>1.5e-05<br>GSC<br>DICER1	rs4510255<br>1.6e-05<br>NA<br>KLHL29	rs12545109<br>1.1e-05<br>SDR16C5<br>PENK	rs213599<br>3.8e-05<br>LINC00398<br>TEX26-AS1	rs2237444<br>1.9e-05<br>GRB10
11	rs9392650<br>2.4e-05<br>RPP40<br>PPP1R3G	rs1264583<br>9.9e-06<br>TRIM39	rs6022321<br>3.1e-05<br>TSHZ2	rs17499929<br>1.6e-05<br>LAP3	rs6126742<br>1.9e-05<br>TSHZ2	rs13227498<br>1.2e-05<br>TPI1P2<br>LOC407835	rs2306910<br>3.9e-05<br>ARHGAP10	rs2329487<br>1.9e-05<br>GRB10
12	rs12741900<br>3.0e-05<br>KCNH1	rs392230<br>1.1e-05<br>GPX6<br>NA	rs4561177<br>3.4e-05<br>C11orf87<br>ZC3H12C	rs1450525<br>1.9e-05<br>LOC101243545	rs516872<br>2.1e-05<br>LINC00347<br>CTAGE11P	rs10059061<br>2.0e-05<br>PCSK1<br>CAST	rs4676617<br>4.0e-05<br>XIRP1<br>CX3CR1	rs4869953<br>2.2e-05<br>MTHFD1L
13	rs314320<br>3.1e-05<br>EPHB4<br>SLC12A9	rs10911467<br>1.2e-05<br>RGL1	rs4480597<br>3.9e-05<br>PRICKLE1<br>ADAMTS20	rs9828938<br>1.9e-05<br>LOC101243545	rs13310635<br>2.1e-05<br>DNAH11	rs1992120<br>2.5e-05<br>SDR16C5<br>PENK	rs11768267<br>4.0e-05<br>DDC	rs2251806<br>2.2e-05<br>NXPH1<br>PER4
14	rs10773097<br>3.5e-05<br>NCOR2<br>SCARB1	rs10752924<br>1.2e-05<br>RGL1	rs1859668<br>3.9e-05<br>EVX1<br>HIBADH	rs685316<br>2.4e-05<br>SORCS1	rs2339704<br>2.4e-05<br>NA<br>KLHL29	rs11707887<br>3.0e-05<br>NMNAT3	rs9353392<br>4.3e-05<br>SNHG5<br>HTR1E	rs9911615<br>2.8e-05<br>KRT12<br>KRT20
15	rs16962812<br>3.7e-05<br>MEP1B	rs6474262<br>1.2e-05<br>ZMAT4	rs1119754<br>4.0e-05<br>SALL1<br>C16orf97	rs669061<br>2.4e-05<br>SORCS1	rs9966612<br>2.5e-05<br>CLUL1	rs550170<br>3.0e-05<br>ECI2<br>CDYL	rs12679499<br>4.9e-05<br>TPD52	rs2430608<br>3.1e-05<br>BCL2L14&ETV6
16	rs8098766<br>3.7e-05<br>MEP1B	rs12212092<br>1.2e-05<br>TRIM10	rs7615965<br>4.1e-05<br>TNIK	rs2027801<br>2.5e-05<br>MTRNR2L6<br>PRSS1	rs877979<br>2.5e-05<br>PDP1<br>CDH17	rs10098993<br>3.8e-05<br>SDR16C5<br>PENK	rs4282339<br>5.0e-05<br>SLIT3	rs12414011<br>3.5e-05<br>EMX2<br>RAB11FIP2
17	rs8095196<br>3.7e-05<br>MEP1B	rs2514552<br>1.6e-05<br>LINC01298<br>C8orf37	rs1867802<br>4.4e-05<br>MOGAT1<br>ACSL3	rs7215803<br>2.9e-05<br>SPECC1	rs7920214<br>2.6e-05<br>FZD8<br>NA	rs17095398<br>4.3e-05<br>SHTN1	rs7410540<br>5.1e-05<br>PPP6R2	rs278391<br>3.7e-05<br>PDZRN3<br>CNTN3
18	rs9963993<br>3.7e-05<br>MEP1B<br>GAREM1	rs1588794<br>1.7e-05<br>BHLHE40-AS1	rs4699496<br>4.7e-05<br>PDHA2<br>NA	rs9478745<br>2.9e-05<br>ARID1B	rs12746576<br>3.0e-05<br>EDARADD	rs2492367<br>5.1e-05<br>DISC1	rs1501702<br>5.9e-05<br>DAB2<br>NA	rs7616034<br>4.3e-05<br>PROK2<br>LINC00870
19	rs1560407<br>3.8e-05<br>SC5D<br>SORL1	rs2514551<br>1.7e-05<br>LINC01298<br>C8orf37	rs9635952<br>4.9e-05<br>DYM	rs7550930<br>3.0e-05<br>PTPRU	rs238120<br>3.2e-05<br>TGIF1<br>DLGAP1	rs7433057<br>5.2e-05<br>ADAMTS9-AS2<br>MAGI1	rs2161994<br>6.0e-05<br>RAPGEF4	rs4622658<br>5.0e-05<br>NA<br>LOC389033
20	rs10954851<br>3.9e-05<br>MIR548O2	rs2339704<br>1.8e-05<br>NA<br>KLHL29	rs608517<br>5.0e-05<br>CCR7<br>SMARCE1	rs7618391<br>3.3e-05<br>LOC101243545	rs2040663<br>3.6e-05<br>DNAH11	rs2298785<br>5.8e-05<br>DOK6	rs5000749<br>6.3e-05<br>HCCAT5<br>LINC01568	rs9403121<br>5.1e-05<br>LOC100132735
21	rs2255464<br>3.9e-05<br>MATN2&RPL30	rs1111280<br>1.9e-05<br>ZMAT4	rs12460230<br>5.1e-05<br>ZNF540	rs178954<br>3.3e-05<br>DIO2-AS1	rs3844534<br>3.8e-05<br>STON2	rs7283383<br>6.2e-05<br>RSPH1	rs1876527<br>6.6e-05<br>SNHG5<br>HTR1E	rs1719980<br>5.8e-05<br>EPB41L3
22	rs7993465<br>4.0e-05<br>LINC00550<br>KLHL1	rs1866026<br>1.9e-05<br>LRP1B	rs488447<br>5.6e-05<br>GABRG1<br>GABRA2	rs12411833<br>3.4e-05<br>DYDC2	rs6039899<br>4.1e-05<br>SNAP25<br>MKKS	rs7193473<br>6.7e-05<br>CMTM1	rs6475448<br>6.6e-05<br>MLLT3	rs2845889<br>6.0e-05<br>MACROD1
23	rs11815834<br>4.1e-05<br>DOCK1	rs2722416<br>2.1e-05<br>ZMAT4	rs12466268<br>5.9e-05<br>FAM117B	rs10122587<br>3.4e-05<br>VLDLR<br>KCNV2	rs4719677<br>4.4e-05<br>DNAH11	rs623400<br>6.7e-05<br>DENND2D<br>CHI3L2	rs704785<br>6.8e-05<br>HPCAL4<br>PPIE	rs326755<br>6.4e-05<br>NA<br>KCNA4
24	rs11044737<br>4.1e-05<br>AEBP2<br>LOC100506393	rs2599656<br>2.1e-05<br>ZMAT4	rs1868877<br>6.5e-05<br>LOC729950<br>ZNF521	rs12548726<br>3.6e-05<br>NA<br>ZFHX4-AS1	rs7980576<br>5.8e-05<br>PKP2<br>SYT10	rs12371268<br>7.0e-05<br>TESC	rs2650044<br>6.9e-05<br>TMEM132D<br>LOC100190940	rs7165173<br>7.4e-05<br>FMN1
25	rs1033824<br>4.4e-05<br>FARS2<br>NRN1	rs10876730<br>2.2e-05<br>OR10A7<br>OR6C74	rs930698<br>6.5e-05<br>LOC729950<br>ZNF521	rs10483761<br>3.6e-05<br>KCNH5	rs4719678<br>5.9e-05<br>DNAH11	rs1534993<br>7.3e-05<br>CDKAL1<br>SOX4	rs11617984<br>7.4e-05<br>LINC00442<br>RNU6-52P	rs1995454<br>7.5e-05<br>PROK2<br>LINC00870
26	rs8131597<br>4.5e-05<br>GRIK1<br>CLDN17	rs4711174<br>2.2e-05<br>GPX6<br>NA	rs1869156<br>6.9e-05<br>ERC2	rs10046325<br>3.6e-05<br>PARK2	rs17814011<br>6.1e-05<br>ZDHHC17	rs11681700<br>7.4e-05<br>HPCAL1	rs250341<br>7.9e-05<br>MYO10	rs17636783<br>7.5e-05<br>NA<br>KCNT2
27	rs3762179<br>4.7e-05<br>FOXL1<br>C16orf95	rs7302063<br>2.4e-05<br>OR10A7<br>OR6C74	rs7906770<br>6.9e-05<br>TCERG1L<br>LINC01164	rs3737807<br>3.7e-05<br>SMPDL3B	rs1570881<br>6.2e-05<br>DCT<br>TGDS	rs2065219<br>8.0e-05<br>ECI2<br>CDYL	rs2272287<br>8.2e-05<br>IQCE	rs12714781<br>7.6e-05<br>PROK2<br>LINC00870
28	rs4735521<br>4.9e-05<br>MATN2	rs7915131<br>2.8e-05<br>ZNF365	rs12938178<br>7.1e-05<br>CDRT7<br>PMP22	rs7766377<br>3.8e-05<br>PARK2	rs10490807<br>6.4e-05<br>ST6GAL1<br>RPL39L	rs7314617<br>8.7e-05<br>MAP1LC3B2<br>C12orf49	rs984119<br>8.4e-05<br>PTPRD<br>NA	rs3132332<br>7.7e-05<br>RXRA<br>COL5A1
29	rs4732956<br>5.2e-05<br>DUSP4<br>LINC00589	rs2439630<br>2.9e-05<br>LINC01298<br>C8orf37	rs1919780<br>7.1e-05<br>DKK1<br>MBL2	rs2259599<br>3.8e-05<br>TMCO3<br>TFDP1	rs2990536<br>6.6e-05<br>FZD8<br>ANKRD30A	rs17257349<br>9.0e-05<br>IRX3<br>CRNDE	rs3757318<br>9.2e-05<br>CCDC170	rs1424883<br>8.9e-05<br>EYA1<br>MSC
30	rs6996857<br>5.2e-05<br>DUSP4<br>LINC00589	rs1864726<br>3.0e-05<br>CTAGE11P<br>TBC1D4	rs701307<br>7.3e-05<br>SEMA3E<br>SEMA3A	rs11057121<br>4.1e-05<br>LMO3<br>SKP1P2	rs167049<br>6.9e-05<br>ST6GALNAC3	rs11177160<br>9.1e-05<br>MDM1	rs7215341<br>9.2e-05<br>NA	rs1336188<br>9.0e-05<br>MIR603
31	rs4733133<br>5.4e-05<br>LEPROTL1&MIR548O2	rs756426<br>3.2e-05<br>AKAP13	rs1484009<br>7.4e-05<br>TMEM132D	rs9972707<br>4.5e-05<br>LINC00922<br>CDH5	rs4978827<br>7.1e-05<br>PTPN3<br>PALM2	rs461964<br>9.2e-05<br>KIFC1	rs9643749<br>9.3e-05<br>TPD52	rs6970449<br>9.3e-05<br>XRCC2<br>ACTR3B
32	rs10830566<br>5.4e-05<br>MIR4490<br>NA	rs911887<br>3.2e-05<br>SFTPD	rs6773938<br>8.5e-05<br>MOBP<br>MYRIP	rs8536<br>4.6e-05<br>IFT43	rs10245983<br>7.4e-05<br>DNAH11	rs2582379<br>9.9e-05<br>PENK<br>LINC00968	rs16874378<br>9.3e-05<br>CLNK<br>MIR572	rs12664111<br>9.4e-05<br>LOC100132735
	rs1837712	rs209857			rs879122		rs1846703	

	HGF	P.Selectin	MCP.1	OPN	TSP.2	Ang2	IGFBP.1	IGFBBP.3
1	rs1344220<br>8.6e-07<br>XIRP1<br>CX3CR1	rs7665177<br>1.1e-06<br>NPFFR2<br>ADAMTS3	rs2284284<br>6.7e-08<br>NRCAM	rs1019800<br>1.9e-06<br>ADAMTS18	rs10431808<br>8.9e-06<br>CLN6	rs6789188<br>1.5e-06<br>NPHP3-AS1<br>TMEM108	rs11672269<br>1.5e-06<br>ZNRF4<br>TINCR	rs1785739<br>5.1e-06<br>ZNF521<br>SS18
2	rs10900490<br>2.4e-06<br>LEMD1<br>MIR135B	rs4876805<br>4.1e-06<br>SAMD12	rs1269650<br>7.4e-07<br>NRCAM	rs4731401<br>1.1e-05<br>SND1<br>MIR129-1	rs7162430<br>1.1e-05<br>PIAS1	rs4280636<br>1.5e-06<br>NPHP3-AS1<br>TMEM108	rs6802458<br>3.4e-06<br>ERC2	rs2350842<br>9.5e-06<br>NXPH1<br>PER4
3	rs787650<br>3.2e-06<br>MYOF	rs1425263<br>7.0e-06<br>GIMD1<br>DKK2	rs2111203<br>1.0e-06<br>NRCAM	rs17770042<br>1.7e-05<br>ADAMTS18	rs6713620<br>1.1e-05<br>B3GNT2<br>TMEM17	rs17229725<br>2.6e-06<br>ZNF438	rs4235245<br>8.8e-06<br>DCHS2	rs10513767<br>1.2e-05<br>PEX5L<br>TTC14
4	rs4730197<br>3.4e-06<br>CCDC71L<br>PIK3CG	rs2340988<br>9.4e-06<br>PRKCB	rs2267878<br>1.2e-06<br>NRCAM	rs2073241<br>2.5e-05<br>PAX9	rs7166850<br>1.1e-05<br>CLN6	rs7918150<br>2.6e-06<br>ZNF438	rs6828176<br>8.8e-06<br>DCHS2	rs1522117<br>1.2e-05<br>PEX5L<br>TTC14
5	rs6675524<br>4.3e-06<br>MIR135B<br>CDK18	rs4555714<br>1.6e-05<br>LARP7<br>ANK2	rs17220280<br>1.4e-06<br>RGPD3<br>ST6GAL2	rs2073246<br>2.5e-05<br>PAX9	rs10180659<br>1.1e-05<br>NCK2	rs11170702<br>7.2e-06<br>CALCOCO1<br>HOXC13-AS	rs4941690<br>1.1e-05<br>LINC00371	rs1058010<br>1.4e-05<br>NAA30
6	rs6692041<br>4.3e-06<br>MIR135B<br>CDK18	rs9832424<br>1.8e-05<br>PARP15<br>PARP14	rs1868255<br>1.4e-06<br>RGPD3<br>ST6GAL2	rs10984490<br>2.5e-05<br>BRINP1	rs8038236<br>1.6e-05<br>PIAS1	rs11170706<br>7.2e-06<br>CALCOCO1<br>HOXC13-AS	rs11159638<br>2.8e-05<br>NA	rs10513770<br>1.5e-05<br>PEX5L<br>TTC14
7	rs2490198<br>1.1e-05<br>SPAG17<br>TBX15	rs10508810<br>2.1e-05<br>PARD3	rs9388833<br>1.8e-06<br>TMEM200A<br>SMLR1	rs12919350<br>3.1e-05<br>RBFOX1	rs6907825<br>1.7e-05<br>LINC01590	rs10876503<br>7.2e-06<br>CALCOCO1<br>HOXC13-AS	rs4267616<br>3.2e-05<br>LRRC3B<br>NEK10	rs17339157<br>1.6e-05<br>FHIT
8	rs2825928<br>1.5e-05<br>NA<br>LINC00320	rs1364808<br>2.2e-05<br>GIMD1<br>DKK2	rs9402271<br>1.8e-06<br>TMEM200A<br>SMLR1	rs2115350<br>3.2e-05<br>LINC01019	rs8031079<br>1.9e-05<br>PIAS1	rs752675<br>7.2e-06<br>CALCOCO1<br>HOXC13-AS	rs3784545<br>3.2e-05<br>TMED3	rs4804310<br>2.0e-05<br>MYO1F
9	rs7276605<br>1.7e-05<br>HLCS	rs1373262<br>2.2e-05<br>GIMD1<br>DKK2	rs6970096<br>1.8e-06<br>NRCAM	rs7073062<br>3.5e-05<br>ARHGAP21<br>PRTFDC1	rs4917978<br>2.1e-05<br>SUFU	rs12889159<br>7.3e-06<br>NID2<br>PTGDR	rs11158157<br>3.4e-05<br>AP5M1<br>NAA30	rs12431274<br>2.0e-05<br>ARGLU1<br>LINC00551
10	rs7233770<br>1.7e-05<br>MTCL1	rs2205796<br>2.2e-05<br>MYLIP	rs11693050<br>2.8e-06<br>RGPD3<br>ST6GAL2	rs10984477<br>4.1e-05<br>BRINP1	rs10786696<br>2.1e-05<br>SUFU	rs2331<br>1.1e-05<br>TMEM108	rs6074113<br>3.5e-05<br>SNAP25	rs9869897<br>2.3e-05<br>PDZRN3<br>CNTN3
11	rs12086423<br>1.8e-05<br>MIR135B<br>CDK18	rs11733406<br>2.4e-05<br>GIMD1<br>DKK2	rs3136630<br>7.2e-06<br>IL15RA	rs11024789<br>4.1e-05<br>PTPN5	rs11071986<br>2.4e-05<br>CALML4	rs11935545<br>1.3e-05<br>TENM3	rs7781415<br>4.4e-05<br>AMPH<br>FAM183BP	rs2350835<br>2.3e-05<br>NXPH1<br>PER4
12	rs2193186<br>1.8e-05<br>MGST1<br>LMO3	rs7695009<br>2.4e-05<br>GIMD1<br>DKK2	rs1269621<br>8.4e-06<br>NRCAM	rs2033074<br>4.7e-05<br>ESRP1	rs3811047<br>3.3e-05<br>IL37	rs11038546<br>1.7e-05<br>HBG2<br>TRIM5	rs17453503<br>4.9e-05<br>ADGRL4<br>NA	rs17189091<br>2.8e-05<br>NA<br>ASB3
13	rs1984880<br>2.1e-05<br>NUGGC	rs16823195<br>2.5e-05<br>C1orf21	rs1269629<br>9.4e-06<br>NRCAM	rs13163497<br>4.9e-05<br>ITGA1	rs4818649<br>3.5e-05<br>LINC00317<br>LINC00308	rs17832939<br>1.8e-05<br>ARID4A	rs1869156<br>5.4e-05<br>ERC2	rs7893147<br>3.0e-05<br>MKI67<br>NA
14	rs1056607<br>2.7e-05<br>LBR	rs5744761<br>2.6e-05<br>POLE	rs12534073<br>9.8e-06<br>NRCAM	rs208549<br>5.3e-05<br>NPVF<br>RNU6-16P	rs914464<br>3.7e-05<br>KCNQ2	rs236965<br>1.9e-05<br>ATXN1	rs11154260<br>5.5e-05<br>NKAIN2	rs6482670<br>3.0e-05<br>MKI67<br>NA
15	rs7994222<br>3.0e-05<br>LINC00297<br>LINC00544	rs13141555<br>2.8e-05<br>GIMD1<br>DKK2	rs17252076<br>1.7e-05<br>FAM124A	rs752653<br>5.6e-05<br>LINC01019	rs965033<br>4.3e-05<br>FHIT	rs7633217<br>2.2e-05<br>NPHP3-AS1<br>TMEM108	rs11986236<br>5.9e-05<br>SNTB1<br>HAS2	rs394431<br>4.2e-05<br>MIR3977<br>TMEM167A
16	rs12259464<br>3.9e-05<br>PRKG1	rs2081453<br>2.8e-05<br>GIMD1<br>DKK2	rs2194941<br>1.8e-05<br>PARM1<br>LOC441025	rs6590334<br>5.9e-05<br>ETS1	rs10076429<br>4.6e-05<br>EDIL3<br>NA	rs4867697<br>2.5e-05<br>NKX2-5<br>STC2	rs1564569<br>5.9e-05<br>SNTB1<br>HAS2	rs13435787<br>4.2e-05<br>NPFFR2
17	rs2793823<br>4.0e-05<br>ADAM30	rs2727668<br>2.9e-05<br>MIR3685<br>METAP2	rs1577217<br>1.9e-05<br>CD109<br>COL12A1	rs4493851<br>5.9e-05<br>KLHDC10<br>TMEM209	rs757459<br>4.8e-05<br>CTNND2	rs8034917<br>2.6e-05<br>PRTG<br>NEDD4	rs2123183<br>5.9e-05<br>ERC2	rs17043794<br>4.3e-05<br>NA<br>ASB3
18	rs872069<br>4.2e-05<br>LPAR1<br>OR2K2	rs17635673<br>4.0e-05<br>KCNIP4	rs11967836<br>2.5e-05<br>LRRC16A	rs1245390<br>7.0e-05<br>MIR4307<br>LINC00645	rs7498094<br>5.3e-05<br>CALML4	rs1571377<br>2.9e-05<br>SEL1L<br>NA	rs1381798<br>6.0e-05<br>XYLB	rs10282765<br>4.6e-05<br>TSPYL5<br>MTDH
19	rs6442749<br>4.4e-05<br>CNTN4	rs10516537<br>4.2e-05<br>GIMD1<br>DKK2	rs6923011<br>3.1e-05<br>CD109<br>COL12A1	rs17315882<br>7.2e-05<br>NRG3<br>GHITM	rs1174957<br>5.5e-05<br>CDCA7L<br>RAPGEF5	rs220601<br>3.3e-05<br>LEF1-AS1<br>RPL34-AS1	rs1488219<br>6.1e-05<br>LRRC3B<br>NEK10	rs821378<br>4.6e-05<br>ZNHIT6
20	rs1451897<br>4.5e-05<br>GRB14	rs1437701<br>4.4e-05<br>LINC01304<br>LINC01249	rs6556312<br>3.8e-05<br>RGS14	rs358019<br>7.3e-05<br>CACNA2D3<br>WNT5A	rs1174958<br>5.5e-05<br>CDCA7L<br>RAPGEF5	rs6540940<br>3.6e-05<br>PEX14	rs7428045<br>6.2e-05<br>PROK2<br>LINC00870	rs2396105<br>4.6e-05<br>PRAM1<br>ZNF414
21	rs11701698<br>4.6e-05<br>C21orf62<br>OLIG2	rs17443878<br>4.8e-05<br>STRN	rs17279221<br>4.1e-05<br>GPD2<br>GALNT5	rs7814447<br>7.4e-05<br>CPQ	rs12663865<br>5.6e-05<br>LINC01590	rs4235991<br>3.6e-05<br>LINC00574<br>LOC154449	rs1525510<br>7.3e-05<br>EIF3IP1<br>IMMP2L	rs2042300<br>4.6e-05<br>ZNF414<br>MYO1F
22	rs12806537<br>4.9e-05<br>B3GAT1	rs1008839<br>4.8e-05<br>HEATR5B	rs7793728<br>4.8e-05<br>SEPT7<br>EEPD1	rs1219671<br>7.8e-05<br>H3F3A<br>ACBD3	rs406900<br>5.7e-05<br>SMIM8<br>LINC01590	rs1952373<br>3.8e-05<br>SEL1L<br>NA	rs2459791<br>7.3e-05<br>LOC401177<br>NA	rs6462246<br>5.1e-05<br>GHRHR<br>ADCYAP1R1
23	rs2333967<br>4.9e-05<br>EEF2KMT<br>RBFOX1	rs6079461<br>5.1e-05<br>MACROD2	rs7519611<br>5.2e-05<br>HIVEP3<br>GUCA2B	rs395489<br>7.8e-05<br>TMEM132B	rs10171167<br>6.0e-05<br>SH3RF3	rs6455655<br>3.9e-05<br>FNDC1<br>SOD2	rs1243701<br>8.0e-05<br>RNASE10<br>RNASE9	rs6483543<br>5.2e-05<br>PTPN5<br>MRGPRX1
24	rs4287433<br>4.9e-05<br>LINC00443<br>FAM155A	rs6875936<br>5.4e-05<br>LOC285692<br>FAM173B	rs1009283<br>5.5e-05<br>OTOS<br>GPC1	rs727670<br>8.8e-05<br>MIR4255<br>LINC01137	rs2745492<br>6.1e-05<br>MIR4255<br>LINC01137	rs11597112<br>4.2e-05<br>ZNF438<br>ZEB1-AS1	rs17412329<br>8.3e-05<br>ELP3<br>PNOC	rs11024865<br>5.2e-05<br>PTPN5<br>MRGPRX1
25	rs10900489<br>5.0e-05<br>LEMD1	rs757411<br>5.6e-05<br>CCR7<br>SMARCE1	rs4718560<br>5.7e-05<br>STAG3L4<br>NA	rs11625200<br>9.7e-05<br>NRXN3	rs6543342<br>6.4e-05<br>NCK2	rs7644746<br>4.4e-05<br>PARL	rs4604357<br>8.3e-05<br>EIF3IP1<br>IMMP2L	rs4570601<br>5.2e-05<br>ME3<br>PRSS23
26	rs2793830<br>5.2e-05<br>NOTCH2	rs2216050<br>5.9e-05<br>TMEM106B<br>VWDE	rs7999900<br>5.8e-05<br>TNFSF13B<br>MYO16	rs11154260<br>1.0e-04<br>NKAIN2	rs372543<br>6.6e-05<br>MIS18BP1<br>LINC00871	rs10518615<br>4.4e-05<br>NA<br>PCDH10	rs6540940<br>8.6e-05<br>PEX14	rs213366<br>5.4e-05<br>FHIT
27	rs11576263<br>5.4e-05<br>SPAG17<br>TBX15	rs9462589<br>6.3e-05<br>MOCS1<br>LINC00951	rs3806448<br>5.9e-05<br>CHIAP2<br>CHIA	rs17195725<br>1.1e-04<br>NOBOX<br>TPK1	rs6925475<br>6.9e-05<br>GPLD1	rs16869977<br>4.7e-05<br>DAB2<br>PTGER4	rs17078172<br>9.1e-05<br>BASP1P1<br>SGCG	rs2965174<br>5.5e-05<br>CEACAM16<br>BCL3
28	rs2392933<br>5.9e-05<br>CCDC71L<br>PIK3CG	rs11791192<br>6.5e-05<br>ASTN2	rs977795<br>6.6e-05<br>MATN2	rs680951<br>1.1e-04<br>PFKFB3	rs1355285<br>7.2e-05<br>CCDC190	rs4744942<br>4.8e-05<br>PTAR1<br>C9orf135-AS1	rs7335200<br>9.1e-05<br>BASP1P1<br>SGCG	rs6702266<br>5.7e-05<br>NA<br>KCNT2
29	rs2407581<br>6.1e-05<br>NA<br>LINC00320	rs2514975<br>6.9e-05<br>SAMD12	rs315084<br>6.9e-05<br>ST6GALNAC3	rs10112364<br>1.2e-04<br>ESRP1	rs4761581<br>7.4e-05<br>CRADD<br>PLXNC1	rs10201913<br>5.6e-05<br>ECEL1	rs9875417<br>9.8e-05<br>CNTN6<br>CNTN4	rs17172499<br>5.8e-05<br>RAMP3<br>ADCY1
30	rs3803804<br>7.0e-05<br>RTN4RL1	rs10893545<br>7.3e-05<br>KIRREL3	rs1240265<br>7.3e-05<br>RAB3IP<br>CNOT2	rs1177986<br>1.2e-04<br>LINC00673	rs2001595<br>7.5e-05<br>SUFU<br>TRIM8	rs6812946<br>5.6e-05<br>DTHD1<br>MIR4801	rs1395479<br>1.0e-04<br>NEIL3<br>AGA	rs9572448<br>6.3e-05<br>ATXN8OS<br>LINC00348
31	rs4676617<br>7.5e-05<br>XIRP1<br>CX3CR1	rs919997<br>7.6e-05<br>CRADD<br>PLXNC1	rs9394270<br>7.7e-05<br>ADTRP	rs158805<br>1.2e-04<br>ARHGEF28<br>ENC1	rs1243701<br>7.6e-05<br>RNASE10<br>RNASE9	rs16996944<br>6.1e-05<br>LEF1-AS1<br>RPL34-AS1	rs9563014<br>1.0e-04<br>GUCY1B2	rs2922851<br>6.4e-05<br>MCPH1
32	rs956675<br>7.6e-05<br>C1QTNF7	rs12599126<br>7.6e-05<br>ACSF3	rs1269662<br>7.8e-05<br>NRCAM	rs6456557<br>1.2e-04<br>NA<br>NRSN1	rs10055024<br>7.7e-05<br>CTNND2	rs744825<br>6.5e-05<br>LINC00457<br>NBEA	rs10488631<br>1.0e-04<br>IRF5<br>TNPO3	rs8099172<br>6.6e-05<br>ZNF521<br>SS18
	rs9557926	rs12587870	rs6546731	rs930026		rs2357442		

	PAI1.tot	ICAM.1	PEDF	VEGF.R2	PAI1.act	VEGF.R1	VCAM.1	CRP
1	rs2306633<br>3.5e-07<br>ANKRD11	rs11870629<br>3.1e-06<br>ASIC2	rs10893525<br>4.9e-06<br>KIRREL3	rs3886314<br>1.7e-06<br>F3<br>SLC44A3	rs4588563<br>6.7e-06<br>RAB9BP1<br>NA	rs16881855<br>6.3e-06<br>LINC00472<br>RIMS1	rs2529243<br>3.2e-06<br>MYL10<br>CUX1	rs10242846<br>6.1e-06<br>AHR<br>SNX13
2	rs11080562<br>1.4e-06<br>CIDEA	rs1468450<br>5.7e-06<br>GRB10	rs17476727<br>9.5e-06<br>KCNV1<br>NA	rs2803407<br>2.5e-06<br>PCSK5	rs6444386<br>1.2e-05<br>TPRG1<br>TP63	rs1192246<br>8.0e-06<br>LINC00472<br>RIMS1	rs9480518<br>7.3e-06<br>PLEKHG1	rs963201<br>1.5e-05<br>LINC00942<br>FBXL14
3	rs2353028<br>3.1e-06<br>ANKRD11	rs2006866<br>7.8e-06<br>CRYBB2P1	rs10097037<br>1.2e-05<br>KCNV1<br>NA	rs2257220<br>2.5e-06<br>PCSK5	rs11762282<br>1.2e-05<br>CARD11<br>SDK1	rs4675615<br>1.1e-05<br>ADAM23	rs1953624<br>8.3e-06<br>NA<br>KHDRBS2	rs8038236<br>2.1e-05<br>PIAS1
4	rs79091<br>6.0e-06<br>CRYBB2P1<br>ADRBK2	rs2285014<br>1.7e-05<br>CDC42BPB	rs987996<br>1.4e-05<br>KCNV1<br>NA	rs9749286<br>3.4e-06<br>PSG6	rs11154583<br>1.4e-05<br>TMEM200A<br>SMLR1	rs16829379<br>1.6e-05<br>NCKAP5<br>MIR3679	rs10245143<br>8.7e-06<br>NPSR1-AS1	rs7162430<br>2.3e-05<br>PIAS1
5	rs238117<br>6.7e-06<br>TGIF1<br>DLGAP1	rs2269323<br>1.7e-05<br>CDC42BPB	rs929051<br>2.3e-05<br>CPNE5	rs1859406<br>3.4e-06<br>PSG6	rs152023<br>1.7e-05<br>ABCC1	rs1429142<br>1.7e-05<br>TTC29<br>EDNRA	rs7113199<br>1.2e-05<br>GLB1L2	rs8031079<br>2.4e-05<br>PIAS1
6	rs2000869<br>1.2e-05<br>GRIK4	rs2013369<br>1.8e-05<br>CRYBB2P1	rs9525638<br>2.6e-05<br>AKAP11<br>TNFSF11	rs11987304<br>7.0e-06<br>PDE7A<br>DNAJC5B	rs12658762<br>1.8e-05<br>NA<br>CDH18	rs2714888<br>2.4e-05<br>TTC29<br>EDNRA	rs4937877<br>1.2e-05<br>GLB1L2	rs4579228<br>2.9e-05<br>OTP<br>TBCA
7	rs6531191<br>1.4e-05<br>OSR1<br>LINC00954	rs938712<br>2.0e-05<br>LOC100507391<br>XXYLT1	rs1437917<br>3.0e-05<br>CPS1<br>ERBB4	rs698964<br>7.9e-06<br>SLC44A3	rs6774354<br>1.9e-05<br>MON1A	rs4132816<br>2.7e-05<br>PCDH9<br>NA	rs6656660<br>1.3e-05<br>DPYD&DPYD-AS1	rs10431808<br>3.1e-05<br>CLN6
8	rs2614079<br>1.9e-05<br>ELP3<br>PNOC	rs2403583<br>3.2e-05<br>DBX1<br>HTATIP2	rs9533154<br>3.8e-05<br>TNFSF11	rs7961210<br>8.8e-06<br>TSPAN9<br>PRMT8	rs949745<br>2.2e-05<br>LINC00924<br>NR2F2-AS1	rs263576<br>3.6e-05<br>BNC2<br>CNTLN	rs12482570<br>1.4e-05<br>KCNJ6	rs9557595<br>3.1e-05<br>NALCN
9	rs1571571<br>2.0e-05<br>PBX3<br>MVB12B	rs11024789<br>3.4e-05<br>PTPN5	rs7325635<br>3.8e-05<br>TNFSF11	rs11245122<br>1.4e-05<br>C10orf90<br>DOCK1	rs16844487<br>2.4e-05<br>DOK7<br>LRPAP1	rs7748810<br>4.0e-05<br>LINC01622<br>FOXQ1	rs16900290<br>1.5e-05<br>KHDRBS2	rs7166850<br>4.1e-05<br>CLN6
10	rs768804<br>2.8e-05<br>DCN<br>LINC01619	rs7933344<br>3.6e-05<br>METTL15<br>NA	rs7740752<br>3.8e-05<br>SLC25A51P1<br>NA	rs391833<br>1.7e-05<br>LINC00886	rs7687823<br>2.6e-05<br>MTNR1A<br>FAT1	rs7748266<br>4.0e-05<br>FKBP5	rs6946436<br>2.0e-05<br>RAMP3<br>ADCY1	rs11071986<br>4.3e-05<br>CALML4
11	rs2614071<br>2.9e-05<br>ELP3<br>PNOC	rs4244542<br>3.8e-05<br>METTL15<br>NA	rs12605863<br>5.0e-05<br>SOCS6<br>LINC01541	rs6882155<br>1.8e-05<br>MCC	rs1398765<br>2.6e-05<br>CPNE4	rs263651<br>4.1e-05<br>BNC2<br>CNTLN	rs495587<br>2.1e-05<br>NEDD4L	rs2358866<br>4.5e-05<br>PLXDC2
12	rs507715<br>3.2e-05<br>CRYBB2P1<br>ADRBK2	rs3807549<br>3.9e-05<br>GRB10	rs6571990<br>5.1e-05<br>OR4E2<br>DAD1	rs4458522<br>1.8e-05<br>ARSJ<br>UGT8	rs7609306<br>2.7e-05<br>CCDC85A	rs716970<br>4.1e-05<br>PAQR8	rs10086758<br>2.4e-05<br>HNF4G<br>ZFHX4-AS1	rs10802043<br>4.8e-05<br>SPAG17<br>TBX15
13	rs2780695<br>3.2e-05<br>CRYBB2P1<br>ADRBK2	rs12806537<br>4.1e-05<br>B3GAT1	rs1127745<br>5.3e-05<br>ACOX2	rs6472235<br>1.9e-05<br>PDE7A<br>DNAJC5B	rs4899579<br>3.2e-05<br>IFT43<br>GPATCH2L	rs1536690<br>4.4e-05<br>ZNF169	rs7416<br>2.5e-05<br>AMPD3	rs2884876<br>4.8e-05<br>SPAG17<br>TBX15
14	rs12533809<br>3.3e-05<br>CDHR3	rs6713620<br>4.3e-05<br>B3GNT2<br>TMEM17	rs13434020<br>5.3e-05<br>ACOX2	rs6981354<br>2.0e-05<br>PDE7A<br>DNAJC5B	rs12202427<br>3.3e-05<br>TMEM200A	rs11223150<br>5.0e-05<br>OPCML	rs6910775<br>2.5e-05<br>AIG1	rs371454<br>5.0e-05<br>RANBP6<br>IL33
15	rs5761092<br>3.7e-05<br>CRYBB2P1	rs868134<br>4.3e-05<br>RBFOX3	rs4790229<br>5.3e-05<br>ZMYND15<br>TM4SF5	rs953446<br>2.0e-05<br>ARRDC3-AS1<br>NA	rs11933503<br>3.4e-05<br>NA	rs1498056<br>5.0e-05<br>TERF2IP<br>CNTNAP4	rs3821466<br>2.7e-05<br>ALDH1L1	rs216259<br>5.1e-05<br>FAM65B
16	rs7012528<br>3.8e-05<br>MIR4659B	rs2189977<br>4.3e-05<br>KCND2	rs4334769<br>6.0e-05<br>C4orf33<br>NA	rs11718266<br>2.2e-05<br>ROBO2	rs11124417<br>3.9e-05<br>NA	rs6455655<br>5.7e-05<br>FNDC1<br>SOD2	rs893952<br>3.2e-05<br>B3GAT1	rs823985<br>5.2e-05<br>FSTL4
17	rs10049947<br>4.8e-05<br>FAM13A	rs10245143<br>4.5e-05<br>NPSR1-AS1	rs17536328<br>6.1e-05<br>TNFSF11	rs4882801<br>2.5e-05<br>TMEM132C	rs11956638<br>4.0e-05<br>NDUFS4<br>ARL15	rs10498713<br>5.9e-05<br>HDGFL1<br>NA	rs1413228<br>3.7e-05<br>DPYD-AS1&DPYD	rs823993<br>5.2e-05<br>FSTL4
18	rs682483<br>4.9e-05<br>TGFB2<br>LYPLAL1-AS1	rs6944490<br>4.5e-05<br>KCND2	rs2047039<br>6.2e-05<br>C4orf22	rs2832660<br>2.9e-05<br>CLDN8<br>KRTAP24-1	rs968059<br>4.2e-05<br>NA	rs992105<br>5.9e-05<br>FKBP5	rs2621250<br>3.8e-05<br>NA<br>RAB28	rs2213448<br>5.6e-05<br>LARGE<br>ISX
19	rs4254088<br>5.1e-05<br>HEPACAM	rs3741353<br>4.6e-05<br>OSBPL5	rs4434576<br>6.3e-05<br>HTR5A<br>INSIG1	rs4611452<br>3.1e-05<br>PLCG2	rs3889926<br>4.3e-05<br>HAND2-AS1<br>FBXO8	rs7718220<br>5.9e-05<br>COL23A1	rs3807549<br>3.9e-05<br>GRB10	rs2451125<br>5.8e-05<br>SAMD12
20	rs2161994<br>5.1e-05<br>RAPGEF4	rs4730976<br>4.7e-05<br>KCND2	rs6795193<br>6.6e-05<br>ACOX2	rs7690911<br>3.4e-05<br>LDB2	rs2223071<br>4.3e-05<br>MIR99AHG<br>LINC01549	rs17445954<br>6.2e-05<br>TP63	rs2727996<br>4.0e-05<br>CHL1<br>CNTN6	rs7701985<br>5.9e-05<br>OTP<br>TBCA
21	rs2279349<br>5.3e-05<br>ANKRD11	rs2237441<br>4.8e-05<br>GRB10	rs10507678<br>6.6e-05<br>NA	rs10794108<br>3.6e-05<br>C10orf90<br>DOCK1	rs10002976<br>4.4e-05<br>NA	rs12111515<br>6.6e-05<br>KCNQ5	rs9324336<br>4.1e-05<br>OLFM3	rs6453340<br>5.9e-05<br>OTP<br>TBCA
22	rs970548<br>5.4e-05<br>MARCH8	rs7140671<br>5.3e-05<br>FUT8<br>LINC00238	rs2151197<br>6.9e-05<br>SPRY2<br>NA	rs1860394<br>3.6e-05<br>TSPAN9<br>PRMT8	rs6574271<br>4.5e-05<br>IFT43<br>GPATCH2L	rs11750504<br>6.8e-05<br>SV2C	rs9838717<br>4.1e-05<br>MIR548A3<br>ALCAM	rs6777714<br>6.5e-05<br>PLXNA1<br>TPRA1
23	rs7086046<br>5.4e-05<br>MARCH8	rs6122616<br>5.3e-05<br>SULF2<br>LINC00494	rs3094352<br>7.1e-05<br>LOC285692	rs7078439<br>3.7e-05<br>C10orf90<br>DOCK1	rs10165126<br>4.5e-05<br>HAT1	rs1444803<br>6.9e-05<br>HPSE2	rs16850761<br>4.1e-05<br>MIR548A3<br>ALCAM	rs2514949<br>7.4e-05<br>SAMD12
24	rs6574271<br>5.6e-05<br>IFT43<br>GPATCH2L	rs1105962<br>5.4e-05<br>NTRK3	rs9635033<br>7.6e-05<br>SPRY2<br>NA	rs4494239<br>3.9e-05<br>C10orf90<br>DOCK1	rs13076126<br>4.7e-05<br>PTPRG	rs31313<br>6.9e-05<br>MYO10	rs16850763<br>4.1e-05<br>MIR548A3<br>ALCAM	rs2514750<br>7.4e-05<br>SAMD12
25	rs2169643<br>5.7e-05<br>NECTIN3	rs4770344<br>5.4e-05<br>BASP1P1<br>SGCG	rs522676<br>7.8e-05<br>SARDH	rs9949492<br>3.9e-05<br>RNF152	rs17284917<br>4.8e-05<br>KCNJ15<br>ERG	rs9504743<br>7.1e-05<br>F13A1	rs7626601<br>4.1e-05<br>MIR548A3<br>ALCAM	rs13253524<br>7.4e-05<br>SAMD12
26	rs9996493<br>5.8e-05<br>SHROOM3	rs6466757<br>5.5e-05<br>KCND2	rs4355401<br>7.9e-05<br>C4orf33<br>NA	rs1409202<br>4.1e-05<br>CACNB2	rs817828<br>4.9e-05<br>RAD23B<br>KLF4	rs650342<br>7.4e-05<br>PAQR8	rs11606228<br>4.3e-05<br>GALNT18	rs746552<br>9.3e-05<br>SUFU
27	rs1466540<br>5.9e-05<br>ANKRD11	rs2596551<br>5.5e-05<br>NA	rs1016898<br>8.3e-05<br>LOC285692	rs7977571<br>4.5e-05<br>IGF1<br>LINC00485	rs17641704<br>5.0e-05<br>LINC00905<br>TPM4	rs118188<br>7.7e-05<br>FAM129A	rs7758052<br>4.4e-05<br>PRIM2<br>GUSBP4	rs10012953<br>9.3e-05<br>EGF<br>ELOVL6
28	rs8536<br>6.2e-05<br>IFT43	rs10833220<br>5.8e-05<br>OSBPL5	rs6899720<br>8.3e-05<br>SLC25A51P1<br>NA	rs6861336<br>4.9e-05<br>MCC	rs11024789<br>5.3e-05<br>PTPN5	rs7068299<br>7.8e-05<br>CDH23	rs6437554<br>4.6e-05<br>MIR548A3<br>ALCAM	rs4917978<br>9.4e-05<br>SUFU
29	rs4899579<br>6.5e-05<br>IFT43<br>GPATCH2L	rs8139619<br>5.8e-05<br>YPEL1	rs7698457<br>8.4e-05<br>SPOCK3	rs6797082<br>5.0e-05<br>LINC00886	rs9377127<br>5.6e-05<br>SAMD5<br>SASH1	rs1411790<br>7.8e-05<br>SH3GL2<br>ADAMTSL1	rs6781572<br>4.6e-05<br>MIR548A3<br>ALCAM	rs10786696<br>9.4e-05<br>SUFU
30	rs877979<br>6.9e-05<br>PDP1<br>CDH17	rs2359758<br>6.0e-05<br>PRDM2	rs3919526<br>8.5e-05<br>UMAD1<br>GLCCI1	rs12592193<br>5.2e-05<br>EFL1	rs1837452<br>5.7e-05<br>NA	rs2252680<br>8.1e-05<br>LINC01622<br>FOXQ1	rs6943444<br>4.6e-05<br>NPSR1-AS1	rs6762974<br>9.4e-05<br>MITF<br>FOXP1
31	rs2306815<br>7.1e-05<br>TBC1D9	rs11984204<br>6.0e-05<br>KCND2	rs17135074<br>8.6e-05<br>LINC00701<br>PFKP	rs13141037<br>6.4e-05<br>NA<br>INTU	rs1837449<br>5.7e-05<br>NA	rs7909464<br>8.6e-05<br>LINC00837<br>LYZL1	rs6947764<br>4.6e-05<br>NPSR1-AS1	rs11085914<br>9.5e-05<br>OR7A10<br>OR7A17
32	rs1793467<br>7.1e-05<br>GDPD4	rs7111226<br>6.4e-05<br>DLG2	rs2618114<br>8.8e-05<br>EOMES<br>CMC1	rs10794113<br>6.4e-05<br>C10orf90<br>DOCK1	rs2683529<br>5.7e-05<br>THRB	rs9360630<br>8.9e-05<br>KCNQ5	rs7564090<br>4.7e-05<br>ETAA1<br>C1D	rs17074954<br>1.0e-04<br>CSMD1<br>LOC100287015
	rs350930	rs153676		rs4962605		rs1001919	rs12266162	rs7777234

	TGFb1	TGFb2	VEGF	PDGFbb	IL.8	IGF.1	TGFb.R3
1	rs265008<br>5.8e-06<br>CMYA5<br>MTX3	rs10448137<br>3.3e-06<br>SH2D4A	rs7767396<br>5.8e-09<br>LINC01512<br>C6orf223	rs2709105<br>2.6e-06<br>ELMO1<br>GPR141	rs9478086<br>9.0e-06<br>WDR27	rs2357016<br>8.5e-06<br>NA<br>ASB3	rs7608404<br>6.6e-06<br>ICOS<br>PARD3B
2	rs9483132<br>8.3e-06<br>TMEM200A<br>SMLR1	rs748057<br>4.7e-06<br>STIM2<br>NA	rs9472159<br>4.5e-07<br>LINC01512<br>C6orf223	rs1117380<br>6.7e-06<br>POLR1D<br>GSX1	rs10979955<br>1.2e-05<br>PTPN3<br>PALM2	rs1430553<br>1.5e-05<br>LOC100505817<br>FBXO15	rs6742158<br>9.9e-06<br>MYT1L<br>TSSC1
3	rs4704601<br>9.1e-06<br>CMYA5<br>MTX3	rs12771265<br>4.8e-06<br>C10orf35<br>COL13A1	rs17720205<br>6.9e-06<br>PARG&NCOA4	rs12770083<br>8.6e-06<br>GRID1	rs1356998<br>3.2e-05<br>NA<br>NRSN1	rs7727304<br>3.7e-05<br>MEF2C<br>MIR3660	rs11216816<br>1.3e-05<br>JAML
4	rs11688320<br>1.4e-05<br>TEX41	rs7555212<br>5.7e-06<br>AJAP1<br>MIR4417	rs1081235<br>1.3e-05<br>KIAA1024<br>ST20-MTHFS	rs1364805<br>8.8e-06<br>GIMD1<br>DKK2	rs1979833<br>3.3e-05<br>CDH18<br>GUSBP1	rs1437288<br>4.2e-05<br>LINC01622<br>FOXQ1	rs11216829<br>1.4e-05<br>MPZL3
5	rs2146244<br>1.7e-05<br>STON2	rs6881435<br>8.6e-06<br>C5orf38<br>LINC01019	rs12075234<br>1.4e-05<br>LINC01343<br>RRAGC	rs8045288<br>9.3e-06<br>NPW<br>SLC9A3R2	rs3780813<br>3.4e-05<br>GTF3C4	rs12651295<br>4.3e-05<br>UGT2B17<br>UGT2B7	rs177421<br>1.5e-05<br>MED15
6	rs2042449<br>2.4e-05<br>SLC6A3	rs155514<br>1.1e-05<br>NA<br>GRM7	rs2733103<br>1.6e-05<br>MTHFS&ST20-MTHFS	rs1371901<br>9.4e-06<br>BCHE<br>ZBBX	rs940770<br>4.0e-05<br>EIF3IP1<br>IMMP2L	rs4742014<br>4.4e-05<br>SPATA6L	rs12126324<br>1.6e-05<br>TCEANC2
7	rs7736611<br>3.4e-05<br>MAP1B	rs1025159<br>1.3e-05<br>LOC400794	rs8102495<br>1.7e-05<br>CCDC61	rs17106642<br>1.3e-05<br>ADCK1<br>NRXN3	rs681604<br>4.0e-05<br>LOC399715<br>PRKCQ	rs661907<br>4.8e-05<br>LINC00331<br>RBM26	rs1417657<br>2.1e-05<br>CDYL
8	rs6702877<br>3.8e-05<br>SLC25A24<br>NBPF4	rs11601433<br>2.3e-05<br>NELL1	rs2068188<br>1.8e-05<br>SGOL1-AS1<br>VENTXP7	rs8057913<br>1.4e-05<br>ZNF598<br>NPW	rs13166076<br>4.1e-05<br>CDH6<br>DROSHA	rs2725759<br>5.9e-05<br>CXXC4<br>TET2	rs2298831<br>2.9e-05<br>JAML
9	rs238117<br>3.9e-05<br>TGIF1<br>DLGAP1	rs9528119<br>2.5e-05<br>DIAPH3<br>TDRD3	rs10849023<br>1.8e-05<br>PARP11<br>CCND2	rs3785284<br>1.6e-05<br>NPW<br>SLC9A3R2	rs6873705<br>4.1e-05<br>CDH6<br>DROSHA	rs11024789<br>6.4e-05<br>PTPN5	rs7740233<br>3.6e-05<br>IYD<br>PLEKHG1
10	rs12798614<br>4.5e-05<br>EMSY<br>LRRC32	rs12576288<br>2.7e-05<br>NELL1	rs11611647<br>1.8e-05<br>PARP11<br>CCND2	rs2717926<br>2.3e-05<br>ELMO1<br>GPR141	rs3807549<br>4.7e-05<br>GRB10	rs10210870<br>6.8e-05<br>MYT1L<br>TSSC1	rs12456391<br>3.8e-05<br>BOD1L2<br>ST8SIA3
11	rs3745568<br>5.1e-05<br>C3	rs2922188<br>2.7e-05<br>OSBPL11	rs7105672<br>1.9e-05<br>SLN<br>SLC35F2	rs238117<br>2.4e-05<br>TGIF1<br>DLGAP1	rs6501474<br>5.0e-05<br>NA<br>SOX9	rs6843220<br>6.9e-05<br>UGT2B17<br>UGT2B7	rs1009081<br>3.8e-05<br>BOD1L2<br>ST8SIA3
12	rs13274854<br>5.4e-05<br>ST18	rs1505035<br>2.7e-05<br>FBXL7	rs2175176<br>2.1e-05<br>DPP10	rs12435647<br>2.7e-05<br>DHRS2<br>DHRS4-AS1	rs975737<br>5.0e-05<br>NA<br>SOX9	rs7949751<br>7.1e-05<br>JAML	rs2002628<br>4.1e-05<br>RGS7BP<br>FAM159B
13	rs6133566<br>5.8e-05<br>PLCB1	rs10051783<br>4.3e-05<br>WWC1	rs1501304<br>2.6e-05<br>SHISA9	rs1922130<br>2.8e-05<br>PRKG1	rs6501475<br>5.0e-05<br>NA<br>SOX9	rs12563798<br>7.2e-05<br>NA<br>KCNT2	rs1830792<br>4.2e-05<br>PAN3<br>FLT1
14	rs11147005<br>5.8e-05<br>POLE	rs285438<br>4.6e-05<br>LOC400794&LRRC52	rs8049852<br>2.7e-05<br>SHISA9	rs12686355<br>3.3e-05<br>PIP5K1B	rs17525274<br>5.7e-05<br>MIOS<br>RPA3	rs1122834<br>7.3e-05<br>OPA1<br>LOC647323	rs11588657<br>4.4e-05<br>PARP1<br>C1orf95
15	rs17814011<br>6.5e-05<br>ZDHHC17	rs611917<br>4.8e-05<br>CELSR2	rs3844159<br>3.5e-05<br>BICC1<br>PHYHIPL	rs7920781<br>4.0e-05<br>GRID1	rs1525513<br>5.8e-05<br>EIF3IP1<br>IMMP2L	rs91294<br>7.8e-05<br>GAS2	rs11578771<br>4.4e-05<br>PARP1<br>C1orf95
16	rs2539357<br>6.6e-05<br>PMS2P9<br>CCDC146	rs39027<br>5.7e-05<br>ZNF804B<br>DPY19L2P4	rs3859532<br>5.1e-05<br>LILRA5<br>LILRA4	rs637999<br>4.0e-05<br>BCHE<br>ZBBX	rs1556632<br>6.0e-05<br>LINC00701<br>PFKP	rs2027494<br>7.9e-05<br>ALDH1A1<br>ANXA1	rs17815421<br>4.5e-05<br>TLN2<br>TPM1
17	rs4468284<br>6.7e-05<br>TACC2	rs1045634<br>6.5e-05<br>SBF2	rs3894101<br>5.9e-05<br>BICC1<br>PHYHIPL	rs238120<br>4.2e-05<br>TGIF1<br>DLGAP1	rs4905431<br>6.1e-05<br>C14orf132<br>BDKRB2	rs17036511<br>8.0e-05<br>NPNT<br>TBCK	rs4578968<br>4.5e-05<br>KBTBD8<br>MIR4272
18	rs2287598<br>7.1e-05<br>CPS1	rs2153264<br>7.2e-05<br>TMCO1	rs6866080<br>6.3e-05<br>ZBED3-AS1<br>PDE8B	rs6978014<br>4.5e-05<br>ELMO1<br>GPR141	rs4905296<br>6.4e-05<br>CLMN<br>LINC00341	rs4740791<br>8.5e-05<br>SPATA6L	rs3910564<br>4.7e-05<br>IPO8
19	rs2333025<br>7.1e-05<br>MSI2	rs676491<br>7.3e-05<br>FAM184A	rs7316141<br>6.3e-05<br>A2ML1	rs167510<br>5.1e-05<br>CHST11	rs6501473<br>6.4e-05<br>NA<br>SOX9	rs11025282<br>8.6e-05<br>NAV2	rs6487927<br>4.7e-05<br>IPO8
20	rs1464574<br>7.3e-05<br>LAMP3	rs10840300<br>7.5e-05<br>SBF2-AS1	rs11228985<br>6.5e-05<br>APLNR<br>TNKS1BP1	rs10105543<br>5.9e-05<br>LOC157273<br>TNKS	rs6575508<br>6.6e-05<br>CLMN<br>LINC00341	rs4400341<br>9.2e-05<br>WRN<br>NRG1	rs11949031<br>4.9e-05<br>IRX4<br>IRX2
21	rs10500388<br>7.9e-05<br>SNX29<br>CPPED1	rs1695515<br>7.6e-05<br>CREB5<br>TRIL	rs1558526<br>6.5e-05<br>A2ML1	rs7923262<br>6.7e-05<br>NODAL<br>PALD1	rs7930061<br>7.0e-05<br>GUCY1A2<br>CWF19L2	rs11074678<br>9.4e-05<br>LCMT1	rs1373290<br>5.0e-05<br>KCND3
22	rs17013657<br>8.4e-05<br>PGRMC2<br>JADE1	rs4709845<br>7.8e-05<br>QKI<br>C6orf118	rs1464574<br>6.6e-05<br>LAMP3	rs2569693<br>6.8e-05<br>ICAM4<br>ICAM5	rs6667575<br>7.1e-05<br>E2F2<br>ID3	rs291753<br>9.8e-05<br>SYK<br>LOC100129316	rs873153<br>5.5e-05<br>CTNNA2
23	rs2063213<br>8.7e-05<br>LOC100506122<br>GALNTL6	rs726921<br>8.0e-05<br>C8orf4<br>ZMAT4	rs1470280<br>6.8e-05<br>FAM160A1	rs6682365<br>7.0e-05<br>KIAA1804<br>KCNK1	rs4705064<br>8.1e-05<br>SH3TC2	rs6483617<br>1.0e-04<br>NAV2	rs2879278<br>5.9e-05<br>FOXI1<br>C5orf58
24	rs888115<br>8.8e-05<br>MSI2	rs4703358<br>8.1e-05<br>LOC100289230<br>NA	rs10509533<br>7.1e-05<br>PTEN<br>RNLS	rs4931235<br>7.2e-05<br>PPFIBP1	rs6697145<br>8.3e-05<br>PLA2G4A	rs2714545<br>1.0e-04<br>DCTD<br>FAM92A1P2	rs984977<br>6.0e-05<br>HDDC2<br>LOC643623
25	rs2273574<br>1.0e-04<br>TAF5L	rs4330370<br>8.5e-05<br>SHROOM3	rs11096956<br>7.5e-05<br>TLR10	rs17021600<br>7.4e-05<br>CDC42EP3<br>RMDN2	rs3006195<br>8.7e-05<br>WDR27	rs17074380<br>1.0e-04<br>DCTD<br>FAM92A1P2	rs2193280<br>6.7e-05<br>BOD1L2<br>ST8SIA3
26	rs4978827<br>1.0e-04<br>PTPN3<br>PALM2	rs9960795<br>8.7e-05<br>LINC01539<br>TXNL1	rs4678398<br>7.6e-05<br>CLDN18	rs5498<br>7.8e-05<br>ICAM4	rs4346260<br>8.7e-05<br>ALOX15<br>PELP1	rs12414011<br>1.1e-04<br>EMX2<br>RAB11FIP2	rs10515240<br>6.8e-05<br>PCSK1<br>CAST
27	rs3800003<br>1.1e-04<br>SCAF8	rs636252<br>8.8e-05<br>GPRC6A<br>RFX6	rs10489683<br>8.8e-05<br>CD5L	rs1588794<br>8.1e-05<br>BHLHE40-AS1	rs9916135<br>8.7e-05<br>ALOX15<br>PELP1	rs3118318<br>1.1e-04<br>IZUMO3<br>TUSC1	rs9844657<br>6.9e-05<br>KBTBD8<br>MIR4272
28	rs11080562<br>1.1e-04<br>CIDEA	rs17138063<br>8.8e-05<br>MRM1<br>LHX1	rs17275724<br>9.1e-05<br>GATB	rs150311<br>8.2e-05<br>MIR9-3HG<br>RHCG	rs1468450<br>9.0e-05<br>GRB10	rs12052294<br>1.1e-04<br>CCDC74A<br>C2orf27A	rs130158<br>7.0e-05<br>FAM19A5
29	rs11005382<br>1.2e-04<br>ZWINT<br>MIR3924	rs17736550<br>8.9e-05<br>KRT77	rs2709826<br>9.4e-05<br>FAM160A1	rs12610468<br>8.3e-05<br>OLFM2	rs3817865<br>9.1e-05<br>WDR27	rs6429364<br>1.1e-04<br>PLD5<br>LINC01347	rs6921560<br>7.4e-05<br>SLC2A12<br>HMGA1P7
30	rs6489968<br>1.2e-04<br>TBX3<br>NA	rs4466711<br>8.9e-05<br>CTNNA3	rs6030295<br>1.1e-04<br>PTPRT	rs6847807<br>8.5e-05<br>NA<br>LINC00290	rs11864605<br>9.3e-05<br>WWOX	rs10513638<br>1.2e-04<br>ZNF385D	rs4857008<br>7.6e-05<br>KBTBD8<br>MIR4272
31	rs2035364<br>1.2e-04<br>ZFPM2	rs16883186<br>9.0e-05<br>KCNQ5	rs4958272<br>1.1e-04<br>SLC36A1	rs11150491<br>8.8e-05<br>CDH13	rs606699<br>9.6e-05<br>NA<br>TFAP2A	rs1554718<br>1.2e-04<br>EGFR&EGFR-AS1	rs2623749<br>7.6e-05<br>CSMD1
32	rs13248544<br>1.2e-04<br>ZFPM2	rs9394178<br>9.1e-05<br>LINC01016<br>MIR1275	rs17195186<br>1.1e-04<br>FOXB2<br>VPS13A-AS1	rs6663100<br>9.3e-05<br>ZNF124	rs512848<br>9.8e-05<br>CCK<br>LYZL4	rs2341561<br>1.3e-04<br>NA	rs11216781<br>7.6e-05<br>SCN4B
		rs7735907	rs2586163		rs754335	rs1505073	rs10474415

33	5.5e-05<br>NA<br>LOC100287010	3.5e-05<br>SYT6<br>TRIM33	rs7655488<br>8.8e-05<br>KCNIP4	rs1558377<br>4.7e-05<br>FAM184B	8.2e-05<br>UQCRFS1<br>LOC284395	rs188417<br>9.9e-05<br>NMNAT3	9.7e-05<br>LOC257396<br>FST	rs4836272<br>9.5e-05<br>ALDH7A1	8.1e-05<br>METTL21C<br>CCDC168	8.2e-05<br>NA<br>LINC00911	7.9e-05<br>DYSF<br>CYP26B1	1.2e-04<br>MIR4318<br>NA	rs6833998<br>7.7e-05<br>LINC01060	7.0e-05<br>NID2<br>PTGDR	rs12531711<br>1.0e-04<br>TNPO3	rs17012632<br>7.6e-05<br>LTBP1	7.2e-05<br>NA<br>TENM2	6.8e-05<br>LINC00922<br>CDH5	rs7333466<br>8.9e-05<br>FAM155A	6.4e-05<br>C10orf90<br>DOCK1	rs2883059<br>7.2e-05<br>CAMKV	9.0e-05<br>TMEM38B<br>ZNF462	4.9e-05<br>NRG3<br>GHITM	1.0e-04<br>PODXL<br>PLXNA4	rs1159046<br>1.3e-04<br>CSMD3	9.3e-05<br>GALNT10&SAP30L-<br>AS1	1.2e-04<br>ST20-AS1<br>BCL2A1	rs1233556<br>9.3e-05<br>SHH	1.0e-04<br>MIR4278<br>MIR4454	1.3e-04<br>MIR3977<br>TMEM167A	7.8e-05<br>ARHGEF28<br>ENC1
34	rs11742519<br>5.6e-05<br>SH3TC2	rs9295663<br>3.5e-05<br>LRRC16A	rs2427995<br>9.0e-05<br>SARDH	rs6941971<br>5.1e-05<br>RUNX2<br>CLIC5	rs600640<br>8.5e-05<br>FLT1	rs13415447<br>1.0e-04<br>VIT	rs1500367<br>9.7e-05<br>LOC257396<br>FST	rs1319094<br>9.6e-05<br>PROK2<br>LINC00870	rs3749078<br>8.2e-05<br>MYEOV2	rs17820181<br>8.3e-05<br>PI4KA	rs11591787<br>8.5e-05<br>LBX1-AS1<br>BTRC	rs214005<br>1.3e-04<br>NRXN3	rs2596501<br>8.3e-05<br>HLA-B	rs4901208<br>7.0e-05<br>NID2<br>PTGDR	rs9426506<br>1.0e-04<br>AJAP1<br>MIR4417	rs8017682<br>7.7e-05<br>KLHL33	rs10078<br>7.3e-05<br>AHRR	rs7665177<br>6.9e-05<br>NPFFR2<br>ADAMTS3	rs1892979<br>9.1e-05<br>NCAM1	rs11743216<br>6.8e-05<br>EPB41L4A-<br>AS2<br>APC	rs1029569<br>7.3e-05<br>CDHR3	rs12146826<br>9.1e-05<br>SLC16A7<br>NA	rs1414803<br>5.0e-05<br>NA<br>KHDRBS2	rs193968<br>1.1e-04<br>TANGO6	rs2320399<br>1.3e-04<br>NCKAP5	rs9429821<br>9.3e-05<br>CAMK1G	rs2709815<br>1.2e-04<br>FAM160A1	rs12966131<br>9.3e-05<br>LOC643542<br>TMX3	rs16887939<br>1.0e-04<br>THBS2<br>WDR27	rs10899172<br>1.3e-04<br>UVRAG<br>WNT11	rs7670671<br>7.9e-05<br>LINC00499
35	rs6901615<br>5.8e-05<br>NA<br>C6orf118	rs9468692<br>3.8e-05<br>TRIM10	rs6817554<br>9.2e-05<br>PDHA2<br>STPG2-AS1	rs6934210<br>5.1e-05<br>RUNX2<br>CLIC5	rs946028<br>8.6e-05<br>GATA3<br>NA	rs16956600<br>1.0e-04<br>TK2	rs519575<br>9.8e-05<br>MED29	rs6765942<br>9.7e-05<br>PROK2<br>LINC00870	rs683979<br>8.5e-05<br>LOC643542<br>TMX3	rs10817996<br>8.3e-05<br>ASTN2	rs223848<br>8.9e-05<br>CCL17<br>CIAPIN1	rs4940391<br>1.3e-04<br>NEDD4L<br>MIR122	rs10925073<br>8.4e-05<br>GCSAML	rs7959202<br>7.2e-05<br>KCNA5<br>NTF3	rs2888089<br>1.1e-04<br>ERBB4	rs12802848<br>9.2e-05<br>ME3<br>PRSS23	rs294594<br>8.2e-05<br>MAT2B<br>NA	rs279076<br>7.6e-05<br>DLEU1	rs2214018<br>9.3e-05<br>LOC285692	rs4562370<br>7.2e-05<br>NA<br>RIPK2	rs4787938<br>7.4e-05<br>C16orf82<br>KDM8	rs1828034<br>9.7e-05<br>TTC29<br>EDNRA	rs16850758<br>5.4e-05<br>MIR548A3<br>ALCAM	rs2772350<br>1.1e-04<br>FARP1	rs238120<br>1.4e-04<br>TGIF1<br>DLGAP1	rs2570277<br>1.0e-04<br>STAM	rs1443088<br>1.2e-04<br>FAM160A1	rs11112522<br>9.4e-05<br>C12orf75<br>NUAK1	rs1602325<br>1.0e-04<br>LINC00692<br>LRRC3B	rs12639104<br>1.3e-04<br>ZIC1<br>AGTR1	rs12887676<br>8.2e-05<br>LINC01550<br>C14orf177
36	rs13226395<br>6.1e-05<br>POT1<br>NA	rs10876726<br>3.9e-05<br>OR9K2<br>OR10A7	rs7627972<br>9.7e-05<br>ULK4	rs7658240<br>5.2e-05<br>LAP3	rs1866599<br>8.6e-05<br>COPS7B<br>MIR1471	rs2106342<br>1.1e-04<br>PIK3R5	rs1502522<br>1.0e-04<br>NA	rs4936894<br>1.0e-04<br>VWA5A	rs12588037<br>9.0e-05<br>NA<br>LINC00911	rs4373194<br>8.4e-05<br>BEND4<br>SHISA3	rs11974486<br>9.2e-05<br>NRCAM	rs9930984<br>1.3e-04<br>ADAMTS18	rs2197116<br>8.5e-05<br>ADAMTS9-<br>AS2&MIR548A2	rs11879029<br>7.5e-05<br>SIRT2	rs17117479<br>1.1e-04<br>NXPE2<br>CADM1	rs6749771<br>9.3e-05<br>HOXD1<br>MTX2	rs3744246<br>8.4e-05<br>ORMDL3<br>LRRC3C	rs372543<br>7.8e-05<br>MIS18BP1<br>LINC00871	rs10917632<br>9.5e-05<br>CCDC190<br>RGS4	rs1558070<br>7.2e-05<br>NOD1	rs7124654<br>7.4e-05<br>NA<br>MIR4300	rs191558<br>9.8e-05<br>PTPRG	rs16972537<br>5.6e-05<br>LINC01568<br>PSMD7	rs875361<br>1.1e-04<br>MIR4255<br>LINC01137	rs4686710<br>1.4e-04<br>TRA2B	rs4008031<br>1.0e-04<br>NA<br>TYRP1	rs6862189<br>1.2e-04<br>SGCD	rs17715481<br>9.5e-05<br>IRF1&C5orf56	rs4837412<br>1.1e-04<br>PTGES<br>TOR1B	rs10803398<br>1.3e-04<br>FBLIM1	rs7297455<br>8.2e-05<br>CAPRIN2
37	rs2422008<br>6.3e-05<br>WDPCP	rs10506430<br>4.1e-05<br>NA<br>FAM19A2	rs10446425<br>9.7e-05<br>ULK4	rs1860596<br>5.2e-05<br>FAM184B	rs1606084<br>8.6e-05<br>MIR5091<br>LINC01085	rs10166098<br>1.1e-04<br>LOC100506474<br>FAM84A	rs1393467<br>1.0e-04<br>NA	rs9894091<br>1.1e-04<br>KRT12	rs11705170<br>9.1e-05<br>PI4KA	rs11592115<br>8.6e-05<br>PTEN<br>RNLS	rs10846986<br>1.1e-04<br>TMEM132B<br>LINC00939	rs9615057<br>1.4e-04<br>KIAA1644<br>LDOC1L	rs11672738<br>8.8e-05<br>NOVA2	rs7134150<br>7.8e-05<br>PDE3A	rs2395900<br>1.1e-04<br>LINC00977<br>GSDMC	rs9634972<br>1.0e-04<br>ATXN8OS<br>LINC00348	rs1026507<br>8.5e-05<br>CARTPT<br>MAP1B	rs12538990<br>7.9e-05<br>KCND2	rs2188748<br>9.6e-05<br>CNBD1<br>DCAF4L2	rs2964911<br>7.4e-05<br>MAT2B<br>NA	rs11999260<br>7.5e-05<br>PBX3	rs736831<br>1.0e-04<br>LGI2<br>SEPSECS	rs1689240<br>5.7e-05<br>NA<br>KHDRBS2	rs1645893<br>1.2e-04<br>PLLP<br>CCL22	rs1437898<br>1.4e-04<br>NCKAP5	rs7301968<br>1.0e-04<br>TMEM132C	rs4849358<br>1.2e-04<br>DPP10	rs4804164<br>9.6e-05<br>RGL3	rs10414149<br>1.1e-04<br>SIGLEC5	rs4920004<br>1.3e-04<br>WDR4	rs7706303<br>9.3e-05<br>RGS7BP<br>FAM159B
38	rs2959644<br>6.9e-05<br>ERICH5	rs3751490<br>4.1e-05<br>REM2	rs8069699<br>9.8e-05<br>ZNF750&TBCD	rs1513156<br>5.5e-05<br>TUSC7<br>NA	rs10772019<br>8.8e-05<br>PKP2<br>SYT10	rs7730643<br>1.1e-04<br>MTRR	rs7968928<br>1.0e-04<br>KCNA5<br>NTF3	rs7213460<br>1.2e-04<br>ASIC2<br>CCL2	rs6977689<br>9.1e-05<br>RAMP3<br>ADCY1	rs11705170<br>8.8e-05<br>PI4KA	rs4895746<br>1.1e-04<br>SAMD5<br>SASH1	rs9406322<br>1.4e-04<br>THBS2<br>WDR27	rs9465549<br>9.0e-05<br>ID4<br>MBOAT1	rs7096309<br>7.9e-05<br>ADAMTS14	rs4749618<br>1.1e-04<br>LYZL2<br>ZNF438	rs2044602<br>1.0e-04<br>CNTN4	rs1558377<br>8.5e-05<br>FAM184B	rs7108408<br>8.2e-05<br>METTL15<br>NA	rs9543947<br>1.0e-04<br>TBC1D4<br>COMMD6	rs3926173<br>7.4e-05<br>MAT2B<br>NA	rs6844878<br>7.8e-05<br>DOK7	rs4399964<br>1.0e-04<br>TTC29<br>EDNRA	rs4787732<br>5.9e-05<br>ZKSCAN2<br>HS3ST4	rs12799883<br>1.3e-04<br>TPCN2<br>MYEOV	rs7524877<br>1.5e-04<br>GIPC2<br>MGC27382	rs7215470<br>1.1e-04<br>RBFOX3	rs9396970<br>1.2e-04<br>WDR27	rs7163811<br>9.6e-05<br>CT62<br>THSD4	rs1074358<br>1.1e-04<br>NOBOX<br>TPK1	rs1555171<br>1.3e-04<br>SNHG5<br>HTR1E	rs4493370<br>9.6e-05<br>LINC00884<br>TMEM44-AS1
39	rs991481<br>7.2e-05<br>CNTNAP5	rs1483130<br>4.2e-05<br>MIR670<br>MIR129-2	rs7219521<br>9.8e-05<br>TBCD	rs289471<br>5.5e-05<br>SPATA8<br>LINC00923	rs12712685<br>9.1e-05<br>NA<br>KLHL29	rs221417<br>1.1e-04<br>NRXN3	rs12555938<br>1.0e-04<br>KDM4C<br>TMEM261	rs10012953<br>1.2e-04<br>EGF<br>ELOVL6	rs2835539<br>9.2e-05<br>HLCS	rs5754222<br>9.2e-05<br>SYN3	rs371315<br>1.1e-04<br>NUDT12<br>NA	rs8099014<br>1.4e-04<br>NEDD4L<br>MIR122	rs10173681<br>9.1e-05<br>LINC01119	rs17533927<br>8.2e-05<br>LMNB1<br>MARCH3	rs1389944<br>1.2e-04<br>ADGRL4<br>NA	rs12596564<br>1.0e-04<br>APOOP5<br>CDH8	rs238120<br>9.0e-05<br>TGIF1<br>DLGAP1	rs10112364<br>8.4e-05<br>ESRP1	rs13378796<br>1.0e-04<br>SPRY2<br>NA	rs3764006<br>7.7e-05<br>SLCO1B7	rs9855505<br>8.1e-05<br>IP6K1	rs11750299<br>1.1e-04<br>SV2C	rs6664218<br>6.1e-05<br>USP48	rs1719980<br>1.3e-04<br>EPB41L3	rs10945617<br>1.5e-04<br>FNDC1<br>SOD2	rs7656480<br>1.1e-04<br>SHROOM3	rs9567265<br>1.2e-04<br>ENOX1<br>CCDC122	rs17696124<br>1.0e-04<br>SEC11C<br>GRP	rs2205595<br>1.2e-04<br>RPS10P7<br>NAV1	rs1555172<br>1.3e-04<br>SNHG5<br>HTR1E	rs6716352<br>9.7e-05<br>ARHGAP15
40	rs3821023<br>8.8e-05<br>GALM	rs11171278<br>4.5e-05<br>NEUROD4<br>OR9K2	rs2671523<br>9.9e-05<br>REEP3<br>ANXA2P3	rs2855954<br>5.5e-05<br>NA	rs30360<br>9.7e-05<br>ANKRD55<br>MAP3K1	rs1958628<br>1.1e-04<br>MDGA2	rs4972861<br>1.1e-04<br>RAPGEF4	rs2051115<br>1.2e-04<br>LINC01625<br>LOC100132735	rs747825<br>9.2e-05<br>IQCJ-<br>SCHIP1<br>IL12A	rs11790102<br>9.5e-05<br>ASTN2	rs4749618<br>1.1e-04<br>LYZL2<br>ZNF438	rs4854306<br>1.4e-04<br>FAM150B<br>TMEM18	rs1951486<br>9.3e-05<br>EVL	rs9347131<br>8.4e-05<br>GTF2H5<br>TULP4	rs6702679<br>1.2e-04<br>ARHGEF10L	rs12503480<br>1.0e-04<br>NPFFR2	rs1860596<br>9.1e-05<br>FAM184B	rs12602412<br>8.6e-05<br>RBFOX3	rs1954504<br>1.0e-04<br>SPRY2<br>NA	rs2042066<br>8.0e-05<br>CHCHD3	rs11138869<br>8.3e-05<br>NA<br>TLE1	rs10184275<br>1.2e-04<br>SCN2A	rs9814707<br>6.2e-05<br>MIR548A3<br>ALCAM	rs11735307<br>1.3e-04<br>DOK7<br>LRPAP1	rs17831104<br>1.5e-04<br>RBBP6<br>TNRC6A	rs1371264<br>1.1e-04<br>LINC00470<br>METTL4	rs17398598<br>1.3e-04<br>EPS15<br>OSBPL9	rs5004102<br>1.0e-04<br>OLFM2	rs3006198<br>1.2e-04<br>WDR27	rs869002<br>1.4e-04<br>ADGRF2	rs12972060<br>9.8e-05<br>RHPN2
41	rs10826354<br>8.8e-05<br>ARMC4	rs6993344<br>4.5e-05<br>MROH5<br>MIR4472-1	rs2590549<br>1.0e-04<br>GPC6	rs4665612<br>5.8e-05<br>KLHL29	rs12541318<br>9.9e-05<br>GATA4	rs7192880<br>1.2e-04<br>PRSS22<br>FLYWCH2	rs1875107<br>1.1e-04<br>LINC00578	rs407758<br>1.3e-04<br>FSTL4	rs776686<br>9.6e-05<br>PLA2G4E<br>PLA2G4D	rs156109<br>1.0e-04<br>PDLIM4<br>SLC22A4	rs1929063<br>1.1e-04<br>MIR622<br>LINC00410	rs435062<br>1.4e-04<br>LHX9<br>NEK7	rs1124814<br>9.5e-05<br>RYR2	rs8079888<br>8.6e-05<br>NA<br>SOX9	rs10843030<br>1.3e-04<br>KLHL42<br>PTHLH	rs9841294<br>1.1e-04<br>CNTN4	rs12456583<br>9.3e-05<br>TMEM241	rs4790022<br>8.6e-05<br>RBFOX3	rs2222294<br>1.0e-04<br>MMP16	rs9495557<br>8.2e-05<br>LINC01625<br>LOC100132735	rs13268487<br>8.9e-05<br>PPP2CB	rs1079653<br>1.3e-04<br>IL17REL<br>MLC1	rs10810738<br>6.3e-05<br>CNTLN	rs2514961<br>1.4e-04<br>SAMD12	rs17753596<br>1.5e-04<br>FUT8<br>LINC00238	rs7695015<br>1.1e-04<br>NKX6-1<br>CDS1	rs9915388<br>1.3e-04<br>RNMTL1<br>NXN	rs10196223<br>1.1e-04<br>NA	rs11083692<br>1.3e-04<br>PSG6	rs11726946<br>1.4e-04<br>UGT2B17<br>UGT2B7	rs3746634<br>1.0e-04<br>SIGLEC1
42	rs733153<br>9.5e-05<br>WNT2	rs7974526<br>4.5e-05<br>OR9K2<br>OR10A7	rs2122791<br>1.0e-04<br>LOC729950<br>ZNF521	rs7154720<br>6.1e-05<br>MDGA2	rs10508828<br>9.9e-05<br>FZD8<br>NA	rs2290182<br>1.2e-04<br>CMTM2	rs3114896<br>1.1e-04<br>ANKRD11	rs1505618<br>1.3e-04<br>PSAT1<br>NA	rs1970128<br>1.0e-04<br>NA<br>DIAPH3	rs294611<br>1.1e-04<br>C3orf20<br>FGD5	rs4785239<br>1.1e-04<br>SALL1<br>C16orf97	rs4361366<br>1.4e-04<br>HAND2-<br>AS1<br>FBXO8	rs4491324<br>9.6e-05<br>PARP11<br>CCND2	rs2247989<br>8.8e-05<br>RAB40B	rs12864972<br>1.3e-04<br>DLEU7	rs13077992<br>1.1e-04<br>FAM19A1	rs7021104<br>9.4e-05<br>LHX2<br>NEK6	rs4729040<br>8.8e-05<br>ANKIB1<br>GATAD1	rs4721998<br>1.0e-04<br>RPL23P8<br>SP4	rs684804<br>8.2e-05<br>KDM4E<br>ENDOD1	rs6591963<br>8.9e-05<br>NA<br>MIR4300	rs1036332<br>1.3e-04<br>MIR181A1HG<br>NR5A2	rs11600497<br>6.3e-05<br>MYEOV<br>CCND1	rs283458<br>1.5e-04<br>HAND1<br>MIR3141	rs1357377<br>1.5e-04<br>CCDC85A<br>VRK2	rs12279261<br>1.1e-04<br>NCAM1	rs9833432<br>1.3e-04<br>SOX14<br>CLDN18	rs1386259<br>1.1e-04<br>IRX5<br>IRX6	rs7659038<br>1.4e-04<br>GALNTL6	rs7751153<br>1.4e-04<br>IMPG1<br>HTR1B	rs1793185<br>1.0e-04<br>JAML
43	rs1672879<br>1.0e-04<br>SCARB1	rs7949744<br>4.7e-05<br>SHANK2	rs10026134<br>1.1e-04<br>LINC00499	rs9784763<br>6.2e-05<br>CCDC162P	rs17099399<br>1.0e-04<br>FGF1<br>ARHGAP26	rs8041720<br>1.2e-04<br>ONECUT1<br>WDR72	rs4719601<br>1.1e-04<br>IQCE	rs4780082<br>1.4e-04<br>FMN1	rs2295797<br>1.1e-04<br>TLN1	rs12056021<br>1.1e-04<br>ANKRD7<br>NA	rs1873928<br>1.2e-04<br>IRX1<br>NA	rs3887925<br>1.5e-04<br>ST6GAL1	rs10517998<br>1.0e-04<br>PALLD	rs968665<br>8.8e-05<br>LINC00457<br>NBEA	rs1374357<br>1.3e-04<br>SATB2	rs324125<br>1.1e-04<br>ZNF880	rs11237146<br>9.5e-05<br>GDPD4	rs32635<br>8.9e-05<br>TRIO<br>FAM105A	rs7851008<br>1.1e-04<br>TLE4<br>NA	rs2044706<br>8.4e-05<br>NA	rs12515666<br>9.1e-05<br>NDUFS4<br>ARL15	rs3804043<br>1.3e-04<br>TANGO2	rs10027979<br>6.4e-05<br>SPARCL1<br>DSPP	rs7513840<br>1.5e-04<br>MIR101-1<br>AK4	rs2527032<br>1.6e-04<br>SEMA3A	rs17322529<br>1.1e-04<br>SHROOM3	rs10225194<br>1.3e-04<br>PER4<br>NDUFA4	rs3923050<br>1.1e-04<br>TUSC7<br>NA	rs1508805<br>1.4e-04<br>PRR16<br>FTMT	rs1395479<br>1.4e-04<br>NEIL3<br>AGA	rs1061627<br>1.0e-04<br>RECQL&GOLT1B
44	rs1423658<br>1.0e-04<br>IL7R<br>CAPSL	rs11171276<br>5.0e-05<br>NEUROD4<br>OR9K2	rs2180267<br>1.2e-04<br>AKAP6<br>NPAS3	rs1511939<br>6.5e-05<br>MIR4318<br>MIR924HG	rs194072<br>1.0e-04<br>GABRB2	rs6660522<br>1.3e-04<br>PLXNA2<br>MIR205HG	rs9814780<br>1.2e-04<br>TMIE<br>PRSS50	rs1805958<br>1.4e-04<br>SEMA5A	rs7493101<br>1.1e-04<br>BEGAIN<br>LINC00523	rs890862<br>1.1e-04<br>INSR	rs651862<br>1.2e-04<br>SLC24A3	rs7631039<br>1.5e-04<br>LINC01330<br>GOLIM4	rs7544118<br>1.0e-04<br>ADORA3	rs527982<br>9.4e-05<br>LINC01239	rs205621<br>1.3e-04<br>SEMA4F<br>HK2	rs298419<br>1.1e-04<br>ZNF385D<br>UBE2E2	rs340477<br>9.5e-05<br>CENPE<br>TACR3	rs6018689<br>9.0e-05<br>SULF2<br>LINC00494	rs10957756<br>1.1e-04<br>CRISPLD1<br>HNF4G	rs2787919<br>8.5e-05<br>AKIRIN2	rs6824647<br>9.1e-05<br>SMIM20<br>RBPJ	rs489961<br>1.3e-04<br>KIRREL3-<br>AS3<br>ETS1	rs1503229<br>6.8e-05<br>ETAA1<br>C1D	rs16844487<br>1.5e-04<br>DOK7<br>LRPAP1	rs6601604<br>1.6e-04<br>GATA4	rs1574968<br>1.1e-04<br>LOC100289230<br>NA	rs3899618<br>1.3e-04<br>PDZRN3<br>CNTN3	rs7010934<br>1.2e-04<br>FAM110B	rs13005810<br>1.5e-04<br>MYADML<br>NA	rs1539683<br>1.4e-04<br>SLC24A2<br>MLLT3	rs6494054<br>1.1e-04<br>RNF111
45	rs882015<br>1.1e-04<br>SLIT3	rs11171277<br>5.0e-05<br>NEUROD4<br>OR9K2	rs7162289<br>1.3e-04<br>LOC283710<br>KLF13	rs2058337<br>6.8e-05<br>FAM184B	rs10025535<br>1.0e-04<br>NA	rs10464649<br>1.3e-04<br>CAV2&CAV1	rs726000<br>1.2e-04<br>ST6GAL2<br>RGPD4-AS1	rs2444967<br>1.4e-04<br>FMN1	rs4753826<br>1.1e-04<br>RAB39A<br>CUL5	rs17272603<br>1.1e-04<br>CRX	rs8103247<br>1.2e-04<br>OR7C1<br>OR7A5	rs11117789<br>1.5e-04<br>ESRRG<br>GPATCH2	rs6743832<br>1.1e-04<br>B3GNT2<br>TMEM17	rs7548714<br>9.9e-05<br>REN<br>KISS1	rs6770722<br>1.3e-04<br>CNTN6<br>CNTN4	rs13059227<br>1.1e-04<br>CNTN4	rs3922335<br>9.6e-05<br>PER4<br>NDUFA4	rs4937880<br>9.1e-05<br>GLB1L2	rs17502738<br>1.1e-04<br>ZBTB5	rs9490713<br>8.6e-05<br>TRDN	rs1513375<br>9.3e-05<br>CPNE4	rs1993553<br>1.3e-04<br>F13A1	rs6936302<br>6.9e-05<br>KCNQ5	rs2877516<br>1.5e-04<br>POLQ	rs7654585<br>1.6e-04<br>SMIM20<br>RBPJ	rs9532991<br>1.2e-04<br>DGKH	rs6861029<br>1.3e-04<br>NA<br>NR2F1-AS1	rs7842612<br>1.2e-04<br>NA<br>RIPK2	rs3114922<br>1.5e-04<br>ZSWIM2<br>CALCRL	rs1930656<br>1.4e-04<br>LINC00700<br>LINC00701	rs1492574<br>1.1e-04<br>IYD<br>PLEKHG1
46	rs11047919<br>1.1e-04<br>KRAS	rs11171280<br>5.0e-05<br>NEUROD4<br>OR9K2	rs2578020<br>1.3e-04<br>REEP3<br>ANXA2P3	rs3734102<br>6.9e-05<br>FGF18<br>FBXW11	rs13242030<br>1.0e-04<br>DGKB	rs1555349<br>1.3e-04<br>MIR646HG<br>CDH4	rs889133<br>1.2e-04<br>C19orf81	rs10781126<br>1.4e-04<br>ANXA1<br>NA	rs17820181<br>1.2e-04<br>PI4KA	rs10136356<br>1.1e-04<br>NA<br>LINC00911	rs1265098<br>1.2e-04<br>PSORS1C2&PSORS1C1	rs2824799<br>1.5e-04<br>TMPRSS15	rs1439381<br>1.1e-04<br>KCTD8<br>YIPF7	rs3805739<br>1.0e-04<br>LMNB1	rs12520553<br>1.3e-04<br>LOC728613<br>MIR4277	rs12563798<br>1.2e-04<br>NA<br>KCNT2	rs2017532<br>9.6e-05<br>PER4<br>NDUFA4	rs304963<br>9.2e-05<br>LINC00593<br>TLE3	rs8094020<br>1.1e-04<br>HRH4<br>LOC729950	rs1463168<br>8.9e-05<br>NA	rs6802890<br>9.3e-05<br>CDHR4	rs263580<br>1.3e-04<br>BNC2<br>CNTLN	rs3588<br>7.0e-05<br>NA<br>KHDRBS2	rs6478270<br>1.6e-04<br>ASTN2	rs658672<br>1.6e-04<br>LAMP3	rs7104988<br>1.2e-04<br>NA<br>ETS1	rs7840296<br>1.4e-04<br>SGCZ	rs12680336<br>1.2e-04<br>ST3GAL1<br>ZFAT	rs230174<br>1.6e-04<br>VWA5B1<br>LINC01141	rs2900252<br>1.4e-04<br>ZBTB43	rs9524858<br>1.1e-04<br>ABCC4
47	rs7045961<br>1.1e-04<br>UNQ6494<br>MIR4290HG	rs12303066<br>5.0e-05<br>OR9K2	rs10995804<br>1.3e-04<br>REEP3<br>ANXA2P3	rs6800555<br>7.1e-05<br>TUSC7<br>NA	rs2206161<br>1.0e-04<br>SNAP25<br>MKKS	rs7636990<br>1.4e-04<br>CDCP1<br>TMEM158	rs12469093<br>1.2e-04<br>ST6GAL2<br>RGPD4-AS1	rs7769912<br>1.4e-04<br>LRRC16A	rs9480518<br>1.2e-04<br>PLEKHG1	rs3904166<br>1.1e-04<br>CDH22	rs1588873<br>1.2e-04<br>SPATA16	rs914737<br>1.6e-04<br>MIR4674<br>EGFL7	rs2285014<br>1.1e-04<br>CDC42BPB	rs10999497<br>1.0e-04<br>ADAMTS14	rs2824844<br>1.3e-04<br>TMPRSS15<br>NA	rs11782156<br>1.2e-04<br>NRG1	rs1340128<br>9.7e-05<br>KCNH1	rs2837587<br>9.4e-05<br>DSCAM	rs7279400<br>1.1e-04<br>RRP1B<br>PDXK	rs722903<br>9.1e-05<br>AKIRIN2<br>SPACA1	rs915004<br>9.3e-05<br>TLE4<br>NA	rs6078495<br>1.3e-04<br>BTBD3<br>SPTLC3	rs6119272<br>7.4e-05<br>LOC149950	rs407758<br>1.6e-04<br>FSTL4	rs4567919<br>1.6e-04<br>CCDC85A<br>VRK2	rs2580138<br>1.2e-04<br>LINC00470<br>METTL4	rs9803853<br>1.4e-04<br>FAF1	rs722503<br>1.3e-04<br>FLT1	rs3130501<br>1.6e-04<br>POU5F1	rs6586261<br>1.4e-04<br>NDUFV3	rs11102362<br>1.1e-04<br>KCND3
48	rs1333108<br>1.1e-04<br>PTPRD	rs7306491<br>5.0e-05<br>OR9K2	rs12945120<br>1.3e-04<br>CDRT7<br>PMP22	rs2540677<br>7.1e-05<br>EEPD1	rs11010375<br>1.1e-04<br>FZD8<br>NA	rs2473904<br>1.4e-04<br>ABTB2	rs3184121<br>1.2e-04<br>HEG1	rs2271139<br>1.4e-04<br>NCOR2	rs959149<br>1.3e-04<br>ANXA1<br>RORB	rs2269340<br>1.1e-04<br>AMPD2	rs2275163<br>1.2e-04<br>KMO	rs2870809<br>1.6e-04<br>MDM1<br>RAP1B	rs2269323<br>1.1e-04<br>CDC42BPB	rs6977689<br>1.1e-04<br>RAMP3<br>ADCY1	rs1030467<br>1.4e-04<br>NFRKB	rs854363<br>1.2e-04<br>GZMB<br>STXBP6	rs10836156<br>9.8e-05<br>NAT10	rs7800859<br>1.0e-04<br>NPSR1-AS1	rs17187632<br>1.1e-04<br>HRH4<br>LOC729950	rs7281703<br>9.5e-05<br>MIR99AHG<br>LINC01549	rs4636589<br>9.4e-05<br>SORCS1<br>NA	rs2696895<br>1.4e-04<br>NA<br>FAM84A	rs2380699<br>7.5e-05<br>DDX1<br>MYCNOS	rs6094373<br>1.6e-04<br>MANBAL<br>SRC	rs17033164<br>1.7e-04<br>RGPD3<br>ST6GAL2	rs1540614<br>1.2e-04<br>GBA3	rs5744707<br>1.4e-04<br>POLK	rs4717992<br>1.4e-04<br>ZNF727<br>ZNF735	rs11672269<br>1.6e-04<br>ZNRF4<br>TINCR	rs6702266<br>1.5e-04<br>NA<br>KCNT2	rs1353197<br>1.2e-04<br>HDDC2<br>LOC643623
49	rs2702656<br>1.1e-04<br>NAV2	rs10506343<br>5.0e-05<br>OR9K2<br>OR10A7	rs1919764<br>1.3e-04<br>DKK1<br>MBL2	rs13387871<br>7.1e-05<br>FANCL<br>LINC01122	rs10520203<br>1.1e-04<br>CHST8	rs1446548<br>1.4e-04<br>LOC100506474<br>FAM84A	rs2974428<br>1.3e-04<br>SLIT3	rs4299296<br>1.4e-04<br>ACVR1C	rs9300799<br>1.3e-04<br>SLC10A2<br>MIR548AS	rs12431390<br>1.1e-04<br>NA<br>LINC00911	rs4283453<br>1.3e-04<br>MFSD6	rs10744349<br>1.7e-04<br>LINC00507<br>TMEM132C	rs4805532<br>1.1e-04<br>URI1<br>ZNF536	rs11256069<br>1.1e-04<br>NA	rs1016364<br>1.4e-04<br>NFRKB	rs10816028<br>1.2e-04<br>PTPRD	rs10853121<br>9.8e-05<br>CA10	rs7130896<br>1.0e-04<br>MICAL2	rs2309244<br>1.1e-04<br>NA<br>LINC00290	rs7134795<br>9.6e-05<br>TSPAN9<br>PRMT8	rs17112452<br>9.8e-05<br>SLIT1	rs7836704<br>1.4e-04<br>OC90	rs4795942<br>7.7e-05<br>TMEM132E	rs10500546<br>1.6e-04<br>TANGO6	rs1519900<br>1.7e-04<br>GCFC2<br>LRRTM4	rs7961199<br>1.3e-04<br>LINC01559<br>GRIN2B	rs12635524<br>1.4e-04<br>SOX14<br>CLDN18	rs4652271<br>1.4e-04<br>BRINP2<br>SEC16B	rs4757298<br>1.6e-04<br>INSC	rs6914894<br>1.5e-04<br>ADGRF4<br>OPN5	rs7763686<br>1.2e-04<br>CDYL
50	rs9311962<br>1.1e-04<br>NA<br>GRM7	rs7314219<br>5.0e-05<br>OR9K2<br>OR10A7	rs1387092<br>1.3e-04<br>CNTN6<br>CNTN4	rs6548499<br>7.8e-05<br>ROBO2	rs10800783<br>1.2e-04<br>PHLDA3<br>CSRP1	rs10792774<br>1.5e-04<br>KRTAP5-9<br>KRTAP5-10	rs11688320<br>1.3e-04<br>TEX41	rs9683082<br>1.4e-04<br>NA<br>ALCAM	rs9558032<br>1.3e-04<br>MIR548AS<br>NA	rs1208081<br>1.2e-04<br>TMEM237	rs9321245<br>1.3e-04<br>TMEM200A<br>SMLR1	rs1245383<br>1.7e-04<br>MIR4307<br>LINC00645	rs10152713<br>1.2e-04<br>PIAS1	rs10431808<br>1.2e-04<br>CLN6	rs11641114<br>1.4e-04<br>CDH13	rs324121<br>1.3e-04<br>ZNF880<br>ZNF528	rs1466539<br>1.0e-04<br>ANKRD11	rs12514694<br>1.1e-04<br>PDE8B	rs1925851<br>1.2e-04<br>SOX21<br>ABCC4	rs9843695<br>9.6e-05<br>BBX<br>LINC00635	rs3744246<br>9.9e-05<br>ORMDL3<br>LRRC3C	rs1410664<br>1.4e-04<br>LINC00472<br>RIMS1	rs2530545<br>8.3e-05<br>NPSR1	rs6755093<br>1.7e-04<br>HOXD1<br>MTX2	rs991842<br>1.7e-04<br>NUF2<br>PBX1	rs2031515<br>1.3e-04<br>STAM	rs717211<br>1.5e-04<br>LRRC55<br>APLNR	rs238129<br>1.4e-04<br>TGIF1<br>DLGAP1	rs2174403<br>1.7e-04<br>WWOX	rs1573472<br>1.6e-04<br>NDUFV3	rs4133202<br>1.2e-04<br>ICOS<br>PARD3B

Supplementary File 2. Excel workbook containing raw data used in these analyses.

Sheet 1, CALGB 80303 Clinical Data: clinical data updated relative to that available on dbGAP (Study Accession: phs000250.v1.p1) with subject ID (SUBJID), patient age (age), updated Performance Score (ps_new), updated extent of disease (dzext_new), updated prior radiation therapy status (priorrt_new), and treatment (TREAT_ASSIGNED) for patients from CALGB 80303. Subject IDs align with those in the CALGB 80303 dbGaP submission.

Sheet 2, CALGB 80303 Plasma Markers: Dataset with subject ID (SUBJID) and levels for all 31 plasma proteins for patients from CALGB 80303. Subject IDs align with those in the CALGB 80303 dbGaP submission (Study Accession: phs000250.v1.p1).

Sheet 3, CALGB 80203 Data: Dataset with subject ID (SUBJID), demographic data [race (RACE), ethnicity (ETHNICITY), patient age (AGE), gender (SEX)], clinical data [Performance Status (PERFORMANCE_ID), presence of local metastases (LOCAL_META; 1 is, 2 is)], treatment, genotype calls for rs2284284, rs7767396, and rs7504372, plasma protein levels for VEGF-A, VEGF-C, and MCP1, and tumor VEGF-A isoform (121, 145, 165, 189, and total) levels for patients from CALBG 80203. The dataset also includes subsetting flags to identify patients with available genotype (PGelg), plasma (PLelg), and tumor isoform (TUelg) values.

Sheet 4, CALGB 80303 SNP Annotation: Dataset with rsID, chromosome, start and end position, location (e.g., intron, intergenic, etc.), and containing or flanking gene symbols and Ensembl IDs for SNPs in Supplementary File 1.

Appendices

- **Statistical analysis code.** Analysis code and results in the R statistical environment for CALGB 80303 and CALGB 80203.

- **eQTL analysis code.** Analysis code and results in the R statistical environment for the eQTL analysis for CALGB 80303.

- **Supplementary datasets code.** Code composing the four raw data files used in the above analyses into a single Excel workbook.

Statistical Analysis Code

Genetic analysis of circulating angiogenesis proteins in cancer patients

Data Analysis

Innocenti *et al.*

June 14, 2018

C80303 genotype data preprocessing I

```
tpedfile="/data1/GWAS/80303/CALGB/80303/dbGaPsubmit/80303dbGaP.tped"
tools::md5sum(tpedfile)

## /data1/GWAS/80303/CALGB/80303/dbGaPsubmit/80303dbGaP.tped
##                         "3b7209d1a566d3de9aea19c0992681ae"

tfamfile="/data1/GWAS/80303/CALGB/80303/dbGaPsubmit/80303dbGaP.tfam"
tools::md5sum(tfamfile)

## /data1/GWAS/80303/CALGB/80303/dbGaPsubmit/80303dbGaP.tfam
##                         "19c14b804048976eca40aa7a333c580b"

phenofile="/data1/GWAS/80303/CALGB/80303/dbGaPsubmit/80303dbGaP_pheno.txt"
tools::md5sum(phenofile)

## /data1/GWAS/80303/CALGB/80303/dbGaPsubmit/80303dbGaP_pheno.txt
##                              "fd709473bb5b33eb982e005873b7f555"
```

C80303 genotype data preprocessing II

```
convert.snp.tped(tped=tpedfile,
                 tfam=tfamfile,
                 outfile="/data1/workspace/CALGB80303/GWAS/eQTL/Proc/80303dbGaP.raw",
                 bcast = 10000)
pheno <- read.table(phenofile, header=TRUE)
names(pheno)[1] <- "id"
write.table(pheno,file="/data1/workspace/CALGB80303/GWAS/eQTL/Proc/pheno.txt",quote=FALSE, row.names=FALSE)
df <- load.gwaa.data(phe="/data1/workspace/CALGB80303/GWAS/eQTL/Proc/pheno.txt",
                   gen="/data1/workspace/CALGB80303/GWAS/eQTL/Proc/80303dbGaP.raw",
                   force=T)
gwa294 <- df[df@phdata$GeneticEuropean==1,]
mc1 <- check.marker(gwa294,callrate=0.95,extr.call=0.95,p.level=1e-08,het.fdr=0,maf=0)
gwa294reduced <- gwa294[,!is.element(gwa294@gtdata@snpnames,mc1$nocall)]
mc2 <- check.marker(gwa294reduced,callrate=0.95,extr.call=0.95,p.level=1e-08,het.fdr=0,maf=0.01)
gwa294reduced <- gwa294reduced[,mc2$snpok]
gwa294reducedauto <- gwa294reduced[,!is.element(gwa294reduced@gtdata@chromosome,c("23","24","25","26"))]
gwa294DI <- gwa294reducedauto
save(gwa294DI,file="../Data/80303gwas_294_snp484523_DI.RData")
```

```
gwafname <- "../Data/80303gwas_294_snp484523_DI.RData"
tools::md5sum(gwafname)

## ../Data/80303gwas_294_snp484523_DI.RData
##       "2f4f298f84d01f03f24ba1a1db715260"

attach(gwafname)
```

C80303 eQTL data

- **Supplemental Dataset 1.csv had the clinical variables used for the VEGFApaper. It includes age, TREATMENT_ASSIGNED, ps_new, dzext_new, priorrt_new, PC1,PC2 and PC3. In the dbGaP phenotype files, it had stratificaation factors (ps,dzext and priorrt) which were updated upon the new data query.**
- **Supplemental Dataset 2.csv contains 31 angiogenic proteins expression levels measured by ELISA.**

```
C80303eqtlfname <- "../Data/DIdata/Supplemental Dataset 2.csv"
tools::md5sum(C80303eqtlfname)

## ../Data/DIdata/Supplemental Dataset 2.csv
##        "fe806115c878cfd07f423586584b4734"

C80303eqtldatALL <- read.csv(C80303eqtlfname)

C80303eqtlpdatfname <- "../Data/DIdata/Supplemental Dataset 1.csv"
tools::md5sum(C80303eqtlpdatfname)

## ../Data/DIdata/Supplemental Dataset 1.csv
##        "0023f86475bb7b3fdf0d05da3469510d"

C80303pdat <- read.csv(C80303eqtlpdatfname)
C80303dbGaPpdat <- read.table(phenofile,header=TRUE)
C80303pdat <- left_join(C80303pdat,C80303dbGaPpdat[,c("SUBJID","sex","ostime","osevent")])

rslists <- c("rs7504372","rs2284284","rs7767396","rs910612","rs16873418","rs10738760")
markerlists <- c("VEGF.C","MCP.1","VEGF")

C80303eqtldat <- C80303eqtldatALL[,c("X",markerlists)]
C80303snpdat <- as.character(gwa294DI@gtdata[,rslists])

C80303eqtltophitdat <- merge(C80303pdat,C80303eqtldat,by.x="SUBJID",by.y="X",all.x=TRUE)
C80303eqtltophitdat <- merge(C80303eqtltophitdat,C80303snpdat,by.x="SUBJID",by.y=0,all.x=TRUE)
C80303eqtltophitdat <- mutate(C80303eqtltophitdat,rs7504372add=additive(rs7504372),
                          rs2284284add=additive(rs2284284),
                          rs7767396add=additive(rs7767396),
                          rs910612add=additive(rs910612),
                          rs16873418add=additive(rs16873418),
                          rs10738760add=additive(rs10738760))
```

C80203 genotype and eQTL data

```
C80203eQTLvalidatedatfname <- "../Data/DIdata/Supplemental Dataset 3.csv"
tools::md5sum(C80203eQTLvalidatedatfname)

## ../Data/DIdata/Supplemental Dataset 3.csv
##        "b4ef9c440fdfb960b66ce78b1813ac07"

anadat <- read.csv(C80203eQTLvalidatedatfname)
anadat <- mutate(anadat,rs7504372add=additive(rs7504372),
               rs2284284add=additive(rs2284284),
               rs7767396add=additive(rs7767396),
               VEGFAexp=1/2^(VEGFA165))
```

C80203 eQTL Samples Selection

```
i1 <- !is.na(anadat$rs2284284)|!is.na(anadat$rs7767396)|!is.na(anadat$rs7504372)
i2 <- !is.na(anadat$MCP1)|!is.na(anadat$VEGF)|!is.na(anadat$VEGFC)
i3 <- !is.na(anadat$totalVEGF)

C80203anadat <- anadat[i1|i2|i3,]
dim(C80203anadat)
```

```
## [1] 185  26
```

```
save(C80203anadat,file="../Proc/C80203anadatN185DI.RData")

C80203SNPdat <- anadat[i1,]
dim(C80203SNPdat)
```

```
## [1] 117  26
```

```
save(C80203SNPdat,file="../Proc/C80203SNPdatN117DI.RData")

C80203SNPanadat <- anadat[i1&i2,]
dim(C80203SNPanadat)
```

```
## [1] 114  26
```

```
save(C80203SNPanadat,file="../Proc/C80203SNPanadatN114DI.RData")

C80203SNPTumoranadat <- anadat[i1&i3,]
dim(C80203SNPTumoranadat)
```

```
## [1] 51 26
```

```
save(C80203SNPTumoranadat,file="../Proc/C80203SNPTumoranadatN51DI.RData")

C80203PCRanadat <- anadat[i2&i3,]
dim(C80203PCRanadat)
```

```
## [1] 65 26
```

```
save(C80203PCRanadat,file="../Proc/C80203PCRanadatN65DI.RData")
```

C80203 CHIP-qPCR data

```
chiqdatfname <- "../Data/ChIP-qPCR.xlsx"
tools::md5sum(chiqdatfname)

##             ../Data/ChIP-qPCR.xlsx
## "53d39ab2807bcea71a8c0537134e3e5f"

chiqdat <- read.xls(chiqdatfname,as.is = TRUE,na.string=c("","N/A"))[,1:3]
chiqdat <- chiqdat%>%group_by(Replicate)%>%summarize(CtIgG=mean(IgG..Ct.value.,na.rm=TRUE),
                                                     CtIP=mean(NF.AT1..Ct.value.,na.rm=TRUE))%>%data.frame
```

Summary of C80303 eQTL data

```
table(C80303eqtltophitdat$sex)

##
##   0   1
##  98 118

table(C80303eqtltophitdat$ps_new)

##
##   1   2
## 195  21

table(C80303eqtltophitdat$dzext_new)

##
##   1   2
## 183  33

table(C80303eqtltophitdat$TREAT_ASSIGNED)

##
## Bevacizumab     Placebo
##         111         105
```

Summary of C80203 eQTL data I

Summary of study population of anyone with plasma or SNP or tumor results (N=185).

```
summary(C80203anadat$AGE)

##    Min. 1st Qu.  Median    Mean 3rd Qu.    Max.
##   21.98   53.78   63.23   60.56   69.67   83.30

table(C80203anadat$SEX)

##
## Female   Male
##     74    111

table(C80203anadat$TREAT_ASSIGNED,exclude=NULL)

##
##        FOLFIRI FOLFIRI + Cmab         FOLFOX  FOLFOX + Cmab
##             50             50             42             43

table(C80203anadat$LOCAL_META,exclude=NULL)

##
##   -2    1    2 <NA>
##    1    4  179    1

table(C80203anadat$PERFORMANCE_ID,exclude=NULL)

##
##  0  1
## 94 91
```

Summary of C80203 eQTL data II

Summary of study population of anyone with plasma or SNP or tumor results (N=185).

```
table(C80203anadat$RACE,exclude=NULL)

##
##                                  Asian           Black or African American Native Hawaiian or Pacific Islander
##                                      1                                  19                                   1
##                                  White
##                                    163

table(C80203anadat$ETHNICITY,exclude=NULL)

##
## Hispanic or Latino       Non-Hispanic            Unknown
##                  3                168                 14
```

Summary of C80203 eQTL data III

Summary of study population of the patients with SNP data, plasma data and self-reported White/Non-Hispanic (N=114).

```
summary(C80203SNPanadat$AGE)

##    Min. 1st Qu.  Median    Mean 3rd Qu.    Max.
##   22.91   53.79   63.47   60.75   70.56   83.17

table(C80203SNPanadat$SEX)

##
## Female   Male
##     46     68

table(C80203SNPanadat$TREAT_ASSIGNED,exclude=NULL)

##
##        FOLFIRI FOLFIRI + Cmab         FOLFOX  FOLFOX + Cmab
##             30             34             23             27

table(C80203SNPanadat$LOCAL_META,exclude=NULL)

##
##  -2   1   2
##   1   3 110

table(C80203SNPanadat$PERFORMANCE_ID,exclude=NULL)

##
##  0  1
## 61 53
```

MAF in C80303, C80203 and HapMap CEU.

Rs7767396 MAF in C80303 or C80203 (N=117) is 0.470 and 0.522. And MAF is 0.491 reported by HapMap CEU. The MAF and HWE of all three SNPs ("rs2284284","rs7504372","rs7767396") are reported:

```
##C80303
gdat<-gwa294DI[gwa294DI@phdata$id%in%C80303eqtltophitdat$SUBJID,]
summary(gdat@gtdata[,c("rs2284284","rs7504372","rs7767396")])[,c(1:2,4:8,12)]

##           Chromosome  Position A1  A2 NoMeasured CallRate        Q.2    Pexact
## rs2284284          7 107680456  A   G        216        1 0.29166667 0.2489811
## rs7504372         18   5922085  A   G        216        1 0.08564815 1.0000000
## rs7767396          6  44035028  A   G        216        1 0.46990741 0.4972467

##C80203
table(C80203SNPdat$rs2284284,exclude=NULL)

##
## A/A G/A G/G
##  55  57   5

table(C80203SNPdat$rs7504372,exclude=NULL)

##
##  T/C  T/T <NA>
##   22   92    3

table(C80203SNPdat$rs7767396,exclude=NULL)

##
##  A/A  G/A  G/G <NA>
##   30   50   35    2

##Hapmap CEU
##http://www.ncbi.nlm.nih.gov/projects/SNP/snp_ref.cgi?rs=7767396
```

HWE test in C80203 (N=117).

```
HWE.exact(as.genotype(C80203SNPdat$rs7767396))$p.value

## [1] 0.1906751

HWE.exact(as.genotype(C80203SNPdat$rs7504372))$p.value

## [1] 0.5950514

HWE.exact(as.genotype(C80203SNPdat$rs2284284))$p.value

## [1] 0.04424352
```

rs7767396 and VEGF location .

The locatation was based on hg19/build 142.
dbSNP: build 142
Position: chr6:43927050-43927050.
Band: 6p21.1.

VEGF: chr6:43,737,946-43,754,223.

LD of rs7767396 and other reported SNPs.

Four common variants, rs6921438, rs4416670, rs6993770, and rs10738760 was reported to explain up to 48% of the heritability of serum VEGF-A levels by Debette et al.

```
#rs6921438
dCEU <- get_proxies(chrom = "6", pos = 43957870, window_size = 1e5, pop = "CEU")
dCEU %>% filter(R.squared > 0.85)

##   CHROM      POS        ID REF ALT MAF R.squared D.prime CHOSEN
## 1     6 43940754 rs9296425   T   C   0       Inf       0  FALSE

#rs4416670
dCEU <- get_proxies(chrom = "6", pos = 43982716, window_size = 1e5, pop = "CEU")
dCEU %>% filter(R.squared > 0.85)

##    CHROM      POS         ID REF ALT       MAF R.squared  D.prime CHOSEN
## 1      6 43976268  rs9381273   G   A 0.1666667  1.000000 1.000000  FALSE
## 2      6 43977172 rs11966317   A   G 0.1666667  1.000000 1.000000   TRUE
## 3      6 43977535  rs9357427   A   G 0.1717172  0.964706 1.000000  FALSE
## 4      6 43969302 rs57055115   G   C 0.1717172  0.895409 0.963415  FALSE
## 5      6 43972571  rs7764334   T   C 0.1717172  0.895409 0.963415  FALSE
## 6      6 43973082  rs2273310   T   C 0.1717172  0.895409 0.963415  FALSE
## 7      6 43974106  rs6927996   A   G 0.1717172  0.895409 0.963415  FALSE
## 8      6 43974166  rs6928047   A   C 0.1717172  0.895409 0.963415  FALSE
## 9      6 43978323  rs6909859   A   C 0.1868687  0.870270 1.000000  FALSE
## 10     6 43985842  rs7757763   C   A 0.1868687  0.870270 1.000000  FALSE
## 11     6 43986555  rs1321096   T   C 0.1868687  0.870270 1.000000  FALSE
## 12     6 43987576  rs7768018   A   T 0.1868687  0.870270 1.000000  FALSE
## 13     6 43987787 rs11337688  TA   T 0.1868687  0.870270 1.000000  FALSE
## 14     6 43964440  rs9462952   A   G 0.1767677  0.864119 0.963190  FALSE
## 15     6 43965165  rs3734693   T   C 0.1767677  0.864119 0.963190  FALSE
## 16     6 43966844 rs60805454   C   T 0.1767677  0.864119 0.963190  FALSE

#rs6993770
dCEU <- get_proxies(chrom = "8", pos = 105569300, window_size = 1e5, pop = "CEU")
dCEU %>% filter(R.squared > 0.85)

##   CHROM       POS         ID REF ALT MAF R.squared D.prime CHOSEN
## 1     8 105612558 rs12550584   C   T   0       Inf       0  FALSE
```

LD of rs7767396 and other reported SNPs.

Only rs10738760 and rs3734693 , the proxy of rs4416670 are genotyped for CALGB 80303.

```
LD(genotype (as.character(gwa294DI@gtdata[,"rs7767396"])), genotype(as.character(gwa294DI@gtdata[,"rs10738760"])))

##
## Pairwise LD
## -----------
##                       D          D'         Corr
## Estimates: -0.000779227 0.003601971 -0.003132366
##
##                  X^2   P-value   N
## LD Test: 0.00576929 0.9394542 294

LD(genotype (as.character(gwa294DI@gtdata[,"rs7767396"])), genotype(as.character(gwa294DI@gtdata[,"rs3734693"])))

##
## Pairwise LD
## -----------
##                      D       D'        Corr
## Estimates: -0.01204417 0.149383 -0.06398448
##
##               X^2   P-value   N
## LD Test: 2.40728 0.1207721 294
```

Estimate the portion of the variance of VEGFA explained by rs7767396 in C80303.

```
fit <- rfit(log2(VEGF)~rs7767396add,dat=C80303eqtltophitdat)
summary(fit,overall.test="drop")

## Call:
## rfit.default(formula = log2(VEGF) ~ rs7767396add, data = C80303eqtltophitdat)
##
## Coefficients:
##              Estimate Std. Error t.value   p.value
## (Intercept)   7.46452    0.15203 49.1004 < 2.2e-16 ***
## rs7767396add -0.80872    0.13194 -6.1293 4.192e-09 ***
## ---
## Signif. codes:  0 '***' 0.001 '**' 0.01 '*' 0.05 '.' 0.1 ' ' 1
##
## Multiple R-squared (Robust): 0.1448878
## Reduction in Dispersion Test: 36.25956 p-value: 0
```

Estimate the portion of the variance of VEGFA explained jointly by rs7767396 (proxy for rs6921438), rs910612 (proxy for rs4416670), rs16873418 and rs16873418 in C80303.

```
fit <- rfit(log2(VEGF)~rs7767396add+rs910612add+rs16873418add+rs10738760add,dat=C80303eqtltophitdat)
summary(fit,overall.test="drop")

## Call:
## rfit.default(formula = log2(VEGF) ~ rs7767396add + rs910612add +
##     rs16873418add + rs10738760add, data = C80303eqtltophitdat)
##
## Coefficients:
##               Estimate Std. Error t.value   p.value
## (Intercept)    7.60749    0.26255 28.9749 < 2.2e-16 ***
## rs7767396add  -0.77034    0.12822 -6.0078 8.192e-09 ***
## rs910612add    0.24010    0.13106  1.8320 0.0683694 .
## rs16873418add -0.25315    0.14036 -1.8036 0.0727318 .
## rs10738760add -0.44725    0.12421 -3.6008 0.0003959 ***
## ---
## Signif. codes:  0 '***' 0.001 '**' 0.01 '*' 0.05 '.' 0.1 ' ' 1
##
## Multiple R-squared (Robust): 0.2074524
## Reduction in Dispersion Test: 13.74207 p-value: 0
```

C80303: Tophits JT test.

```
jonckheere.test(x=log2(C80303eqtltophitdat$VEGF),g=C80303eqtltophitdat$rs7767396add)

##
##  Jonckheere-Terpstra test
##
## data:
## JT = 4314, p-value = 5.839e-09
## alternative hypothesis: two.sided

jonckheere.test(x=log2(C80303eqtltophitdat$MCP.1),g=C80303eqtltophitdat$rs2284284add)

##
##  Jonckheere-Terpstra test
##
## data:
## JT = 4156.5, p-value = 6.665e-08
## alternative hypothesis: two.sided

jonckheere.test(x=log2(C80303eqtltophitdat$VEGF.C),g=C80303eqtltophitdat$rs7504372add)

##
##  Jonckheere-Terpstra test
##
## data:
## JT = 5221, p-value = 9.85e-09
## alternative hypothesis: two.sided
```

C80303: Tophits linear regression test.

```
eqtldat <- normalize.quantiles(as.matrix(t(C80303eqtldatALL[,-1])))
eqtldat <- data.frame(t(eqtldat))%>%
  setNames (colnames(C80303eqtldatALL)[-1])%>%
  `row.names<-`(as.character(C80303eqtldatALL$X))%>%
  select(VEGF.C,MCP.1,VEGF)
eqtldat <- eqtldat[as.character(C80303eqtltophitdat$SUBJID),]
summary(lm(eqtldat$VEGF~C80303eqtltophitdat$rs7767396add))$coef

##                                   Estimate Std. Error   t value     Pr(>|t|)
## (Intercept)                      233.33498   18.60646 12.540533 2.080393e-27
## C80303eqtltophitdat$rs7767396add -83.40325   15.97559 -5.220668 4.211185e-07

summary(lm(eqtldat$MCP.1~C80303eqtltophitdat$rs2284284add))$coef

##                                   Estimate Std. Error   t value     Pr(>|t|)
## (Intercept)                       701.8236   28.44235 24.675306 1.639521e-64
## C80303eqtltophitdat$rs2284284add -119.0524   32.06032 -3.713388 2.608908e-04

summary(lm(eqtldat$VEGF.C~C80303eqtltophitdat$rs7504372add))$coef

##                                  Estimate Std. Error   t value     Pr(>|t|)
## (Intercept)                      717.7549   47.50607 15.108698 1.391237e-35
## C80303eqtltophitdat$rs7504372add 532.7769  111.80049  4.765425 3.477215e-06
```

C80303: adjusted plasma VEGF association test with rs7767396.

```
fit <- rfit(log2(VEGF)~rs7767396add+log10(age)+sex,dat=C80303eqtltophitdat)
summary(fit,overall.test="drop")

## Call:
## rfit.default(formula = log2(VEGF) ~ rs7767396add + log10(age) +
##     sex, data = C80303eqtltophitdat)
##
## Coefficients:
##              Estimate Std. Error t.value   p.value
## (Intercept)   4.81788    2.17431  2.2158   0.02777 *
## rs7767396add -0.78897    0.13335 -5.9165 1.306e-08 ***
## log10(age)    1.50170    1.20491  1.2463   0.21402
## sex          -0.14252    0.18424 -0.7735   0.44008
## ---
## Signif. codes:  0 '***' 0.001 '**' 0.01 '*' 0.05 '.' 0.1 ' ' 1
##
## Multiple R-squared (Robust): 0.1529966
## Reduction in Dispersion Test: 12.76471 p-value: 0

fit <- rfit(log2(VEGF)~rs7767396add+PC1+PC2+PC3,dat=C80303eqtltophitdat)
summary(fit,overall.test="drop")

## Call:
## rfit.default(formula = log2(VEGF) ~ rs7767396add + PC1 + PC2 +
##     PC3, data = C80303eqtltophitdat)
##
## Coefficients:
##              Estimate Std. Error t.value   p.value
## (Intercept)   7.41208    0.16974 43.6673 < 2.2e-16 ***
## rs7767396add -0.82018    0.12810 -6.4026 9.749e-10 ***
## PC1           1.37902    1.64869  0.8364   0.40386
## PC2           3.00545    1.50135  2.0018   0.04658 *
## PC3          -3.62971    1.55543 -2.3336   0.02056 *
## ---
## Signif. codes:  0 '***' 0.001 '**' 0.01 '*' 0.05 '.' 0.1 ' ' 1
##
## Multiple R-squared (Robust): 0.1853333
## Reduction in Dispersion Test: 12.0004 p-value: 0
```

C80303: the relation of the VEGF to standard prognostic variables.

```
fit<-rfit(log2(VEGF)~log10(age)+sex,dat=C80303eqtltophitdat)
summary(fit,overall.test="drop")

## Call:
## rfit.default(formula = log2(VEGF) ~ log10(age) + sex, data = C80303eqtltophitdat)
##
## Coefficients:
##             Estimate Std. Error t.value p.value
## (Intercept)  4.24370    2.26939  1.8700 0.06286 .
## log10(age)   1.44339    1.25948  1.1460 0.25307
## sex         -0.33486    0.19043 -1.7585 0.08011 .
## ---
## Signif. codes:  0 '***' 0.001 '**' 0.01 '*' 0.05 '.' 0.1 ' ' 1
##
## Multiple R-squared (Robust): 0.01896464
## Reduction in Dispersion Test: 2.05878 p-value: 0.13014
```

The median circulating VEGFA abundance levels by rs7767396 genotypes in C80303.

```
tapply(C80303eqtltophitdat$VEGF, C80303eqtltophitdat$rs7767396, summary)
```

```
## $`A/A`
##     Min.  1st Qu.   Median     Mean  3rd Qu.     Max.
##    19.00    77.97   195.33   504.02   346.17 12678.20
##
## $`A/G`
##    Min. 1st Qu.  Median    Mean 3rd Qu.    Max.
##   10.93   51.53   95.67  129.17  153.40  957.33
##
## $`G/G`
##    Min. 1st Qu.  Median    Mean 3rd Qu.    Max.
##    5.20   34.00   58.07   80.73  107.00  378.40
```

```
tapply(C80303eqtltophitdat$VEGF, dominant(C80303eqtltophitdat$rs7767396), summary)
```

```
## $`A/A`
##     Min.  1st Qu.   Median     Mean  3rd Qu.     Max.
##    19.00    77.97   195.33   504.02   346.17 12678.20
##
## $`A/G-G/G`
##    Min. 1st Qu.  Median    Mean 3rd Qu.    Max.
##    5.20   46.42   85.40  115.38  138.10  957.33
```

Estimate the portion of the variance of VEGFA explained by rs7767396 in C80203.

```
fit<-rfit(log2(VEGF)~rs7767396add,dat=C80203SNPanadat)
summary(fit,overall.test="drop")

## Call:
## rfit.default(formula = log2(VEGF) ~ rs7767396add, data = C80203SNPanadat)
##
## Coefficients:
##              Estimate Std. Error t.value   p.value
## (Intercept)   8.72592    0.22927 38.0597 < 2.2e-16 ***
## rs7767396add -0.61684    0.14542 -4.2419 4.639e-05 ***
## ---
## Signif. codes:  0 '***' 0.001 '**' 0.01 '*' 0.05 '.' 0.1 ' ' 1
##
## Multiple R-squared (Robust): 0.1394862
## Reduction in Dispersion Test: 17.83061 p-value: 5e-05
```

C80203: adjusted plasma VEGF association test with rs7767396 (N=112).

```
dim(C80203SNPanadatsub)

## [1] 112  26

jonckheere.test(x=log2(C80203SNPanadatsub$VEGF),g=C80203SNPanadatsub$rs7767396add)

##
##  Jonckheere-Terpstra test
##
## data:
## JT = 1284, p-value = 5.184e-05
## alternative hypothesis: two.sided

fit<-rfit(log2(VEGF)~rs7767396add+log10(AGE)+SEX,dat=C80203SNPanadatsub)
summary(fit,overall.test="drop")

## Call:
## rfit.default(formula = log2(VEGF) ~ rs7767396add + log10(AGE) +
##     SEX, data = C80203SNPanadatsub)
##
## Coefficients:
##              Estimate Std. Error t.value   p.value
## (Intercept)   6.49920    1.74763  3.7189 0.0003192 ***
## rs7767396add -0.55901    0.15451 -3.6179 0.0004534 ***
## log10(AGE)    1.33137    0.98024  1.3582 0.1772295
## SEXMale      -0.35865    0.23640 -1.5171 0.1321536
## ---
## Signif. codes:  0 '***' 0.001 '**' 0.01 '*' 0.05 '.' 0.1 ' ' 1
##
## Multiple R-squared (Robust): 0.1665106
## Reduction in Dispersion Test: 7.19191 p-value: 0.00019
```

C80203: the relation of the VEGF to standard prognostic variables.

```
fit<-rfit(log2(VEGF)~log10(AGE)+SEX,dat=C80203SNPanadatsub)
summary(fit,overall.test="drop")

## Call:
## rfit.default(formula = log2(VEGF) ~ log10(AGE) + SEX, data = C80203SNPanadatsub)
##
## Coefficients:
##             Estimate Std. Error t.value  p.value
## (Intercept)  5.47911    1.82857  2.9964 0.003383 **
## log10(AGE)   1.65739    1.03577  1.6002 0.112459
## SEXMale     -0.53931    0.24144 -2.2337 0.027544 *
## ---
## Signif. codes:  0 '***' 0.001 '**' 0.01 '*' 0.05 '.' 0.1 ' ' 1
##
## Multiple R-squared (Robust): 0.06337804
## Reduction in Dispersion Test: 3.68783 p-value: 0.0282
```

The median circulating VEGFA abundance levels by rs7767396 genotypes in C80203.

```
tapply(C80203SNPanadat$VEGF, C80203SNPanadat$rs7767396, summary)

## $`A/A`
##    Min. 1st Qu.  Median    Mean 3rd Qu.    Max.
##    84.0   332.5   529.5   604.3   814.2  1864.0
##
## $`G/A`
##    Min. 1st Qu.  Median    Mean 3rd Qu.    Max.
##    18.6   157.2   230.4   302.7   381.7  1717.2
##
## $`G/G`
##    Min. 1st Qu.  Median    Mean 3rd Qu.    Max.
##    28.3   115.5   199.4   564.2   345.6 11291.5

tapply(C80203SNPanadat$VEGF, dominant(C80203SNPanadat$rs7767396), summary)

## $`A/A`
##    Min. 1st Qu.  Median    Mean 3rd Qu.    Max.
##    84.0   332.5   529.5   604.3   814.2  1864.0
##
## $`G/A-G/G`
##    Min. 1st Qu.  Median    Mean 3rd Qu.    Max.
##    18.6   123.5   227.9   411.7   368.2 11291.5
```

The median circulating VEGFC and MCP1 abundance levels by rs7504372 and rs2284284 genotypes respectively in C80203.

```
tapply(C80203SNPanadat$VEGFC, C80203SNPanadat$rs7504372, summary)

## $`T/C`
##    Min. 1st Qu.  Median    Mean 3rd Qu.    Max.
##   134.1  1188.4  1577.0  1711.9  2176.7  4768.1
##
## $`T/T`
##    Min. 1st Qu.  Median    Mean 3rd Qu.    Max.
##    18.2   667.5  1286.7  1542.9  2236.5  5003.4

tapply(C80203SNPanadat$MCP1,C80203SNPanadat$rs2284284, summary)

## $`A/A`
##    Min. 1st Qu.  Median    Mean 3rd Qu.    Max.
##   240.0   615.6   863.8   885.6  1103.1  1752.5
##
## $`G/A`
##    Min. 1st Qu.  Median    Mean 3rd Qu.    Max.
##   393.8   623.8   936.2  1098.1  1215.0  7486.2
##
## $`G/G`
##    Min. 1st Qu.  Median    Mean 3rd Qu.    Max.
##   253.8   635.0   917.5   776.0   993.8  1080.0
```

The median circulating isoform VEGFA.145 abundance levels by rs7767396 genotypes in C80203.

```
tapply(C80203SNPTumoranadat$VEGF.145, C80203SNPTumoranadat$rs7767396, summary)

## $`A/A`
##    Min. 1st Qu.  Median    Mean 3rd Qu.    Max.    NA's
##    8.67   10.76   11.34   11.22   11.65   13.69       7
##
## $`G/A`
##    Min. 1st Qu.  Median    Mean 3rd Qu.    Max.    NA's
##   8.704   9.824  10.334  10.632  11.439  13.270       6
##
## $`G/G`
##    Min. 1st Qu.  Median    Mean 3rd Qu.    Max.    NA's
##   9.330   9.655   9.966  10.021  10.316  10.880      10
```

The median circulating total isoform VEGFA abundance levels by rs7767396 genotypes in C80203.

```
tapply(C80203SNPTumoranadat$totalVEGF, C80203SNPTumoranadat$rs7767396, summary)
```

```
## $`A/A`
##    Min. 1st Qu.  Median    Mean 3rd Qu.    Max.
##   2.776   4.036   4.960   4.776   5.220   6.752
##
## $`G/A`
##    Min. 1st Qu.  Median    Mean 3rd Qu.    Max.
##   3.844   4.340   5.127   5.167   5.832   7.231
##
## $`G/G`
##    Min. 1st Qu.  Median    Mean 3rd Qu.    Max.
##   3.339   3.837   5.007   4.906   5.405   7.387
```

C80303:effect of VEGF on ostime.

```
summary(coxph(Surv(ostime,osevent)~log2(VEGF),data=C80303eqtltophitdat))

## Call:
## coxph(formula = Surv(ostime, osevent) ~ log2(VEGF), data = C80303eqtltophitdat)
##
##   n= 216, number of events= 214
##
##               coef exp(coef) se(coef)     z Pr(>|z|)
## log2(VEGF) 0.11372   1.12044  0.04594 2.475   0.0133 *
## ---
## Signif. codes:  0 '***' 0.001 '**' 0.01 '*' 0.05 '.' 0.1 ' ' 1
##
##            exp(coef) exp(-coef) lower .95 upper .95
## log2(VEGF)      1.12     0.8925     1.024     1.226
##
## Concordance= 0.573  (se = 0.023 )
## Rsquare= 0.027   (max possible= 1 )
## Likelihood ratio test= 6.01  on 1 df,   p=0.01426
## Wald test            = 6.13  on 1 df,   p=0.01331
## Score (logrank) test = 6.1  on 1 df,   p=0.01354
```

C80303:effect of VEGFC on ostime.

```
summary(coxph(Surv(ostime,osevent)~log2(VEGF.C),data=C80303eqtltophitdat))

## Call:
## coxph(formula = Surv(ostime, osevent) ~ log2(VEGF.C), data = C80303eqtltophitdat)
##
##   n= 216, number of events= 214
##
##                  coef exp(coef) se(coef)      z Pr(>|z|)
## log2(VEGF.C) -0.06698   0.93522  0.05059 -1.324    0.186
##
##              exp(coef) exp(-coef) lower .95 upper .95
## log2(VEGF.C)    0.9352      1.069    0.8469     1.033
##
## Concordance= 0.531  (se = 0.023 )
## Rsquare= 0.008   (max possible= 1 )
## Likelihood ratio test= 1.74  on 1 df,   p=0.1877
## Wald test            = 1.75  on 1 df,   p=0.1855
## Score (logrank) test = 1.75  on 1 df,   p=0.1857
```

C80303:effect of MCP1 on ostime.

```
summary(coxph(Surv(ostime,osevent)~log2(MCP.1),data=C80303eqtltophitdat))

## Call:
## coxph(formula = Surv(ostime, osevent) ~ log2(MCP.1), data = C80303eqtltophitdat)
##
##   n= 216, number of events= 214
##
##               coef exp(coef) se(coef)     z Pr(>|z|)
## log2(MCP.1) 0.2001    1.2215   0.1039 1.925   0.0542 .
## ---
## Signif. codes:  0 '***' 0.001 '**' 0.01 '*' 0.05 '.' 0.1 ' ' 1
##
##             exp(coef) exp(-coef) lower .95 upper .95
## log2(MCP.1)     1.221     0.8187    0.9964     1.497
##
## Concordance= 0.567  (se = 0.023 )
## Rsquare= 0.017   (max possible= 1 )
## Likelihood ratio test= 3.6  on 1 df,   p=0.05769
## Wald test            = 3.71  on 1 df,   p=0.05418
## Score (logrank) test = 3.7  on 1 df,   p=0.05448
```

C80203:effect of VEGF on ostime.

```
C80203osdat <- read.csv("../Data/DIdata/C80203_ostime_data.csv")
C80203anadat <- left_join(C80203anadat,C80203osdat)

## Joining, by = "SUBJID"

summary(coxph(Surv(os_time,os_status)~log2(VEGF),data=C80203anadat))

## Call:
## coxph(formula = Surv(os_time, os_status) ~ log2(VEGF), data = C80203anadat)
##
##   n= 154, number of events= 144
##    (31 observations deleted due to missingness)
##
##               coef exp(coef) se(coef)     z Pr(>|z|)
## log2(VEGF) 0.11535   1.12227  0.06154 1.874   0.0609 .
## ---
## Signif. codes:  0 '***' 0.001 '**' 0.01 '*' 0.05 '.' 0.1 ' ' 1
##
##            exp(coef) exp(-coef) lower .95 upper .95
## log2(VEGF)     1.122     0.8911    0.9947     1.266
##
## Concordance= 0.552  (se = 0.027 )
## Rsquare= 0.022   (max possible= 1 )
## Likelihood ratio test= 3.41  on 1 df,   p=0.06491
## Wald test            = 3.51  on 1 df,   p=0.06088
## Score (logrank) test = 3.49  on 1 df,   p=0.06162
```

C80203:effect of VEGFC on ostime.

```
summary(coxph(Surv(os_time,os_status)~log2(VEGFC),data=C80203anadat))

## Call:
## coxph(formula = Surv(os_time, os_status) ~ log2(VEGFC), data = C80203anadat)
##
##   n= 154, number of events= 144
##    (31 observations deleted due to missingness)
##
##                 coef exp(coef) se(coef)      z Pr(>|z|)
## log2(VEGFC) -0.02830   0.97209  0.05825 -0.486    0.627
##
##             exp(coef) exp(-coef) lower .95 upper .95
## log2(VEGFC)    0.9721      1.029    0.8672      1.09
##
## Concordance= 0.489  (se = 0.027 )
## Rsquare= 0.002   (max possible= 1 )
## Likelihood ratio test= 0.23  on 1 df,   p=0.6288
## Wald test            = 0.24  on 1 df,   p=0.6271
## Score (logrank) test = 0.24  on 1 df,   p=0.6271
```

C80203:effect of MCP1 on ostime.

```
summary(coxph(Surv(os_time,os_status)~log2(MCP1),data=C80203anadat))

## Call:
## coxph(formula = Surv(os_time, os_status) ~ log2(MCP1), data = C80203anadat)
##
##   n= 154, number of events= 144
##    (31 observations deleted due to missingness)
##
##                coef exp(coef) se(coef)     z Pr(>|z|)
## log2(MCP1) 0.03458   1.03518  0.11983 0.289    0.773
##
##            exp(coef) exp(-coef) lower .95 upper .95
## log2(MCP1)     1.035      0.966    0.8185     1.309
##
## Concordance= 0.514  (se = 0.027 )
## Rsquare= 0.001   (max possible= 1 )
## Likelihood ratio test= 0.08  on 1 df,   p=0.7726
## Wald test            = 0.08  on 1 df,   p=0.7729
## Score (logrank) test = 0.08  on 1 df,   p=0.7729
```

ChIP-qPCR Analysis.

Quantitative real time PCR (qRT-PCR) was performed with oligonucleotides spanning rs7767396 (forward: 5'-ACCTGTGAAGATGCCGTTCT-3', reverse: 5'-TGATTTCCATGTTTGGCTCA-3'). Results were normalized to background signal (IgG) and expressed as fold enrichment of signal to background.

```
2^-(chiqdat$CtIP-chiqdat$CtIgG)

## [1] 4.267329 2.351096 2.907945
```

rs7767369 boxplot

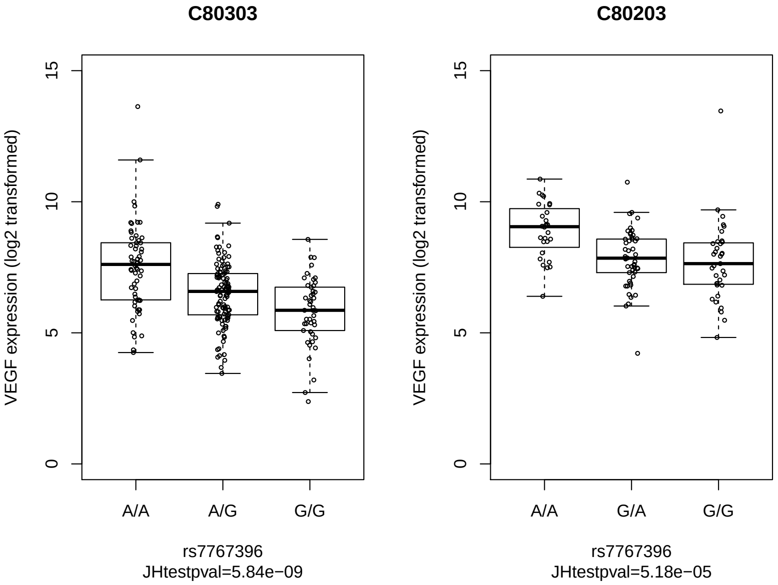

rs7504372 boxplot

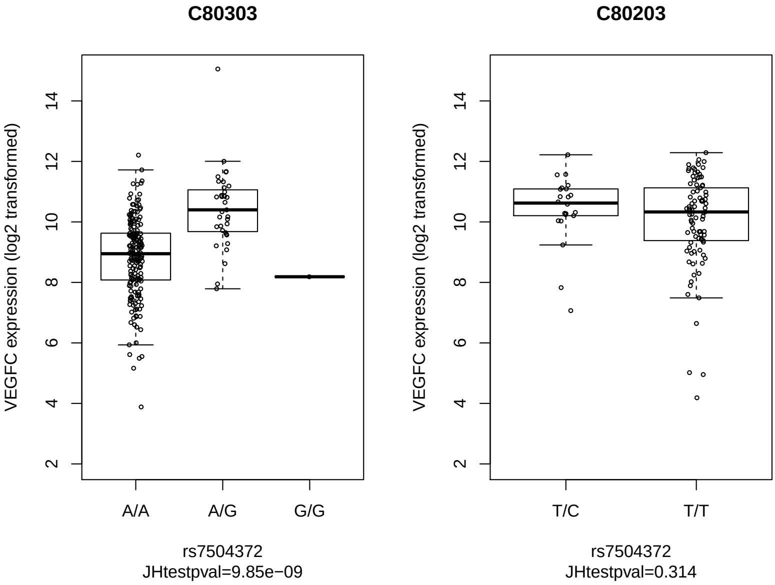

rs2284284 boxplot

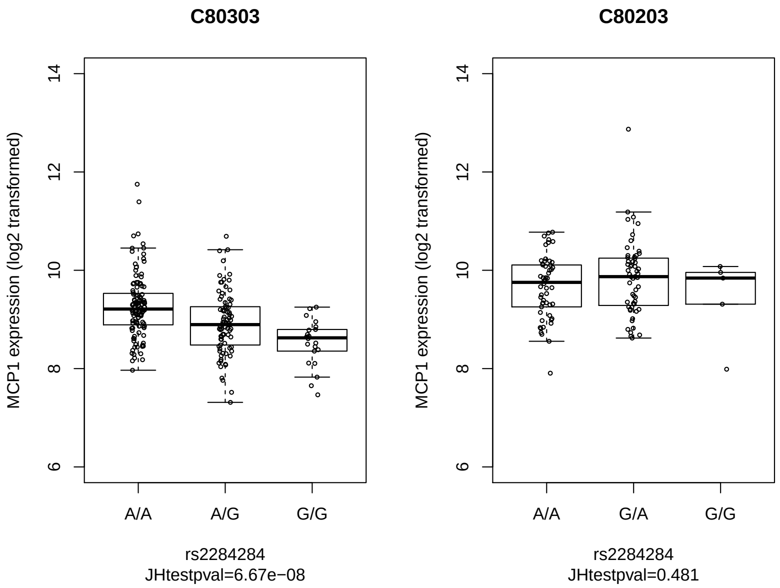

VEGFA isoforms Summary

```
isoformdat=C80203isoformanadat
summary(-isoformdat$VEGF.121)

##    Min. 1st Qu.  Median    Mean 3rd Qu.    Max.    NA's
## -10.445  -7.405  -6.605  -6.535  -5.547  -3.770       3

summary(-isoformdat$VEGF.145)

##    Min. 1st Qu.  Median    Mean 3rd Qu.    Max.    NA's
## -13.687 -11.383 -10.626 -10.649  -9.758  -8.670      49

summary(-isoformdat$VEGF.189)

##    Min. 1st Qu.  Median    Mean 3rd Qu.    Max.    NA's
##  -9.902  -8.251  -7.768  -7.622  -6.986  -4.580       2

summary(-isoformdat$totalVEGF)

##    Min. 1st Qu.  Median    Mean 3rd Qu.    Max.
##  -7.402  -5.637  -5.016  -5.006  -4.277  -2.776

summary(-isoformdat$VEGFA165)

##    Min. 1st Qu.  Median    Mean 3rd Qu.    Max.    NA's
## -12.479  -9.525  -7.520  -8.144  -6.761  -3.916       1
```

Boxplot of VEGFA isoforms in 80203

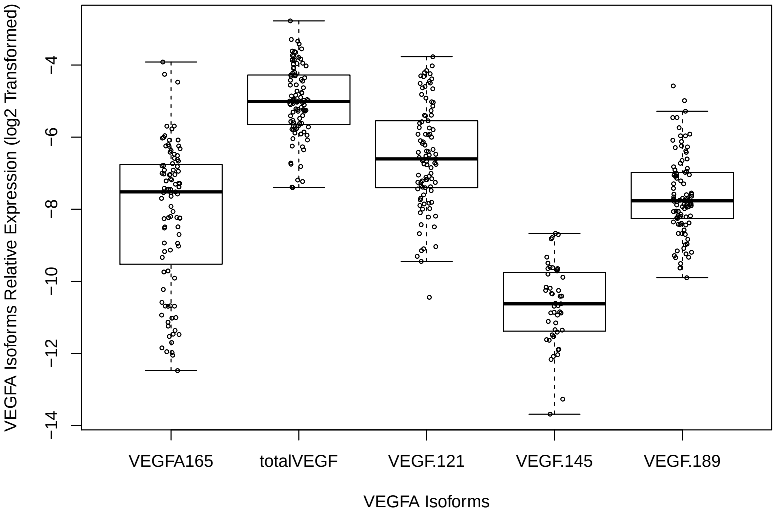

VEGFA isoforms : rs7767369 boxplot

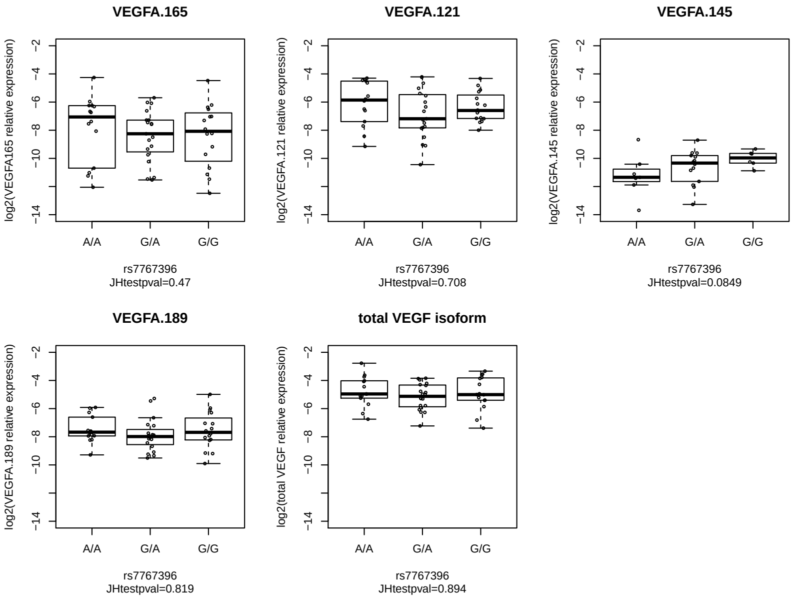

VEGFA isoforms 165 vs rs7767396 and plasma VEGFA

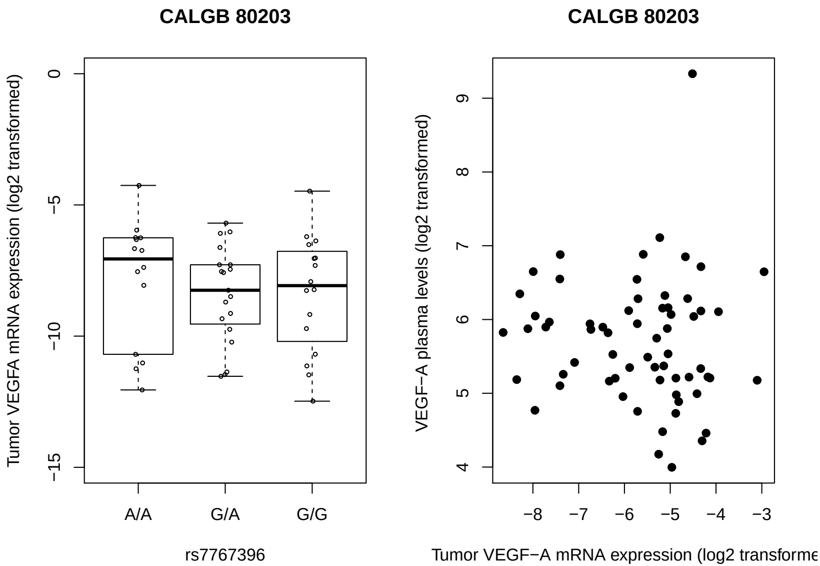

VEGFA isoforms : pairwise plot

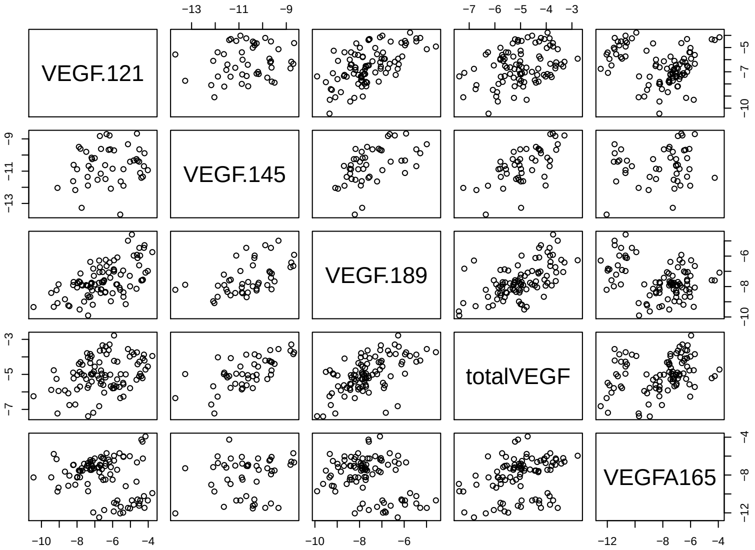

VEGFA scatter plot :log transformed blood VEGFA vs isoform VEGFA in 80203

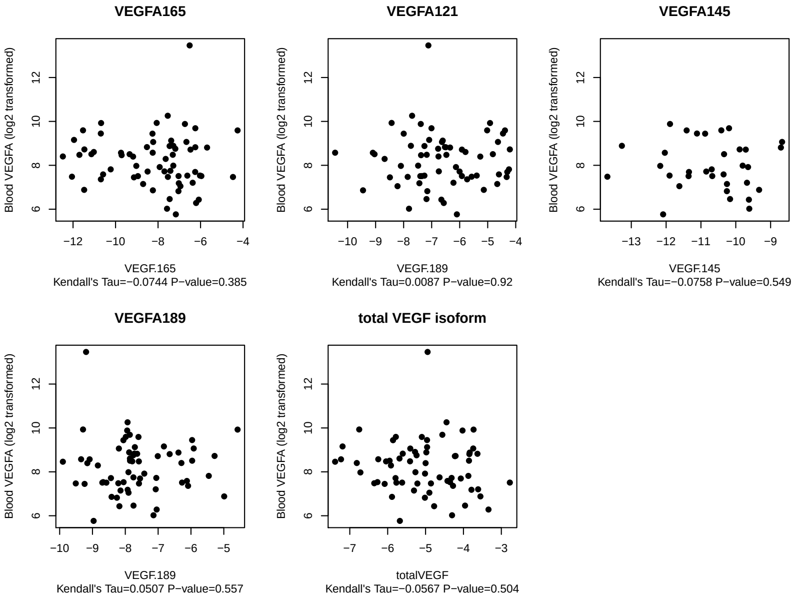

Session Information

- **R version 3.4.4 (2018-03-15)**, `x86_64-pc-linux-gnu`
- **Running under**: `Ubuntu 18.04 LTS`
- **Matrix products: default**
- **BLAS**: `/usr/lib/x86_64-linux-gnu/openblas/libblas.so.3`
- **LAPACK**: `/usr/lib/x86_64-linux-gnu/libopenblasp-r0.2.20.so`
- **Base packages: base, datasets, graphics, grDevices, methods, parallel, stats, utils**
- **Other packages: bindrcpp 0.2.2, clinfun 1.0.15, combinat 0.0-8, dplyr 0.7.5, gdata 2.18.0, GenABEL 1.8-0, GenABEL.data 1.0.0, genetics 1.3.8.1, gtools 3.5.0, haplo.stats 1.7.9, knitr 1.20, lattice 0.20-35, MASS 7.3-49, mvtnorm 1.0-8, preprocessCore 1.40.0, proxysnps 0.0.1, Rfit 0.23.0, SNPassoc 1.9-2, survival 2.41-3**
- **Loaded via a namespace (and not attached): acepack 1.4.1, assertthat 0.2.0, backports 1.1.2, base64enc 0.1-3, bindr 0.1.1, bitops 1.0-6, checkmate 1.8.5, cluster 2.0.6, codetools 0.2-15, colorspace 1.3-2, compiler 3.4.4, data.table 1.11.4, digest 0.6.15, evaluate 0.10.1, foreign 0.8-69, Formula 1.2-3, ggplot2 2.2.1, glue 1.2.0, grid 3.4.4, gridExtra 2.3, gtable 0.2.0, highr 0.7, Hmisc 4.1-1, htmlTable 1.12, htmltools 0.3.6, htmlwidgets 1.2, latticeExtra 0.6-28, lazyeval 0.2.1, magrittr 1.5, Matrix 1.2-12, MatrixModels 0.4-1, multcomp 1.4-8, munsell 0.5.0, nlme 3.1-131, nnet 7.3-12, pillar 1.2.3, pkgconfig 2.0.1, plyr 1.8.4, polspline 1.1.12, purrr 0.2.5, quantreg 5.36, R6 2.2.2, RColorBrewer 1.1-2, Rcpp 0.12.17, RCurl 1.95-4.10, rlang 0.2.1, rms 5.1-2, rpart 4.1-13, rstudioapi 0.7, sandwich 2.4-0, scales 0.5.0, SparseM 1.77, splines 3.4.4, stringi 1.2.2, stringr 1.3.1, TH.data 1.0-8, tibble 1.4.2, tidyselect 0.2.4, tools 3.4.4, zoo 1.8-2**

eQTL Analysis Code

CALGB80303 fastJT Analysis for Top Hits

April 5, 2017

Load Processed Plasma Data

```
plasfile <- "/data1/workspace/CALGB80303/GWAS/eQTL/Proc/S2_Dataset.csv"
tools::md5sum(plasfile)
```

```
## /data1/workspace/CALGB80303/GWAS/eQTL/Proc/S2_Dataset.csv
##                         "fe806115c878cfd07f423586584b4734"
```

```
C80303eQTLdata <- read.csv(plasfile)
rownames(C80303eQTLdata) <- C80303eQTLdata[,1]
C80303eQTLdata <- C80303eQTLdata[,-1]
plasdat <- C80303eQTLdata
dim(plasdat)
```

```
## [1] 216  31
```

```
markers <- colnames(plasdat)[1:31]
markers
```

```
##  [1] "FGFb"      "VEGF.C"    "PlGF"      "GROa"      "PDGF.AA"
##  [6] "SDF.1b"    "VEGF.D"    "IL.6"      "HGF"       "P.Selectin"
## [11] "MCP.1"     "OPN"       "TSP.2"     "Ang2"      "IGFBP.1"
## [16] "IGFBBP.3"  "PAI1.tot"  "ICAM.1"    "PEDF"      "VEGF.R2"
## [21] "PAI1.act"  "VEGF.R1"   "VCAM.1"    "CRP"       "TGFb1"
## [26] "TGFb2"     "VEGF"      "PDGFbb"    "IL.8"      "IGF.1"
## [31] "TGFb.R3"
```

```
plasdat$id <- rownames(plasdat)
```

Load SNP Data and Subset Genetype Data for Markers in Plasma Data

```
snpfile <- "/data1/workspace/CALGB80303/GWAS/eQTL/Data/80303gwas_294_snp484523_DI.RData"
tools::md5sum(snpfile)

## /data1/workspace/CALGB80303/GWAS/eQTL/Data/80303gwas_294_snp484523_DI.RData
##                                           "57f4c94d3176416f52cd0f3c0adada78"

load(snpfile)
dim(gwa294DI@phdata)

## [1] 294  58

dim(gwa294DI@gtdata)

## [1]    294 484523

gwa216 <- gwa294DI[gwa294DI@phdata$id %in% plasdat$id,]
dim(gwa216@phdata)

## [1] 216  58

dim(gwa216@gtdata)

## [1]    216 484523
```

Order Marker Data to Match Genotype Data

```
plasdatm <- plasdat[as.character(gwa216@phdata$id),1:31]
dim(plasdatm)
```

```
## [1] 216  31
```

```
all(plasdatm$id == gwa216@phdata$id)
```

```
## [1] TRUE
```

Run fastJT on 31 Markers for 484523 SNPs

Run fastJT analysis on 31 Markers for 484523 SNPs.

```
bigY <- as.matrix(as.numeric(gwa216@gtdata))
bigX <- as.matrix(plasdatm[,-32])
jtAll <- fastJT(bigX, bigY, outTopN=100, numThreads = 32)
```

Print out marker names that with p-value smaller than 10^{-7}

```
pval <- pvalues(jtAll)
pval[1,pval[1,]<1e-7]


##       VEGF.C        MCP.1         VEGF
## 9.849625e-09 6.664854e-08 5.838963e-09
```

Save Results

Save results to .RData.

```
fname <- paste0(outdir,"fastJT_484523x31_",Sys.Date(),"DI_.RData")
save(jtAll, file=fname)
tools::md5sum(fname)
```

```
## ./fastJT_484523x31_2017-04-05DI_.RData
##     "2e5aa2651f806e23ff59d7eca1b8e538"
```

Write full results to csv.

```
csvname <- paste0(outdir,"C80303_fastJT_484523x31_DI.csv")
write.csv(pvalues(jtAll), file=csvname, quote=F)
tools::md5sum(csvname)
```

```
##   ./C80303_fastJT_484523x31_DI.csv
## "7b46259aa623d57f83ec580d02eef36f"
```

Save the Top 50 Hits SNPs per Protein to File for Annotation

Save the top 50 hits SNP IDs to file.

```
topX <- 50
TopSNPs <- jtAll$XIDs[1:topX,]
TopPs <- pvalues(jtAll)[1:topX,]
rsids <- unique(as.vector(TopSNPs))
rsname <- paste0(outdir,"rsIDs.txt")
write.table(rsids, file=rsname, quote=F, row.names=F, col.names=F)
tools::md5sum(rsname)


##                        ./rsIDs.txt
## "5da3d45cebbffc51974a9622a19d07a1"
```

Annotation

- The list of 1550 top SNPs (1478 unique) is annotated with chromosome and position info from `SNPlocs.Hsapiens.dbSNP144.GRCh37`. (One SNP cannot be mapped).
- Gene locations are taken from `TxDb.Hsapiens.UCSC.hg19.knownGene`.
- The `locateVariants()` function from the `VariantAnnotation` extension package is used to identify genes containing or immediately flanking each SNP.
  - Note: For 17 intergenic SNPs, no genes are within the specified search ranges.
  - `IntergenicVariants(upstream = 1e+06L, downstream = 1e+06L)`
  - `PromoterVariants(upstream = 2000L, downstream = 200L)`
- The `org.Hs.eg.db` data base is used to convert the resulting Entrez Gene IDs into gene symbols.
- Multiple symbols are returned for 66 SNPs, so matches are prioritized by location, i.e., coding >intron >fiveUTR >threeUTR >promoter >intergenic.
- 16 of these SNPs have overlapping symbols (i.e., two symbols with the top-most priority of location), which are combined with "&".

Save the Top 50 Hits per Protein to HTML File

Load annotation data.

```
snpfname <- "/data1/workspace/CALGB80303/GWAS/eQTL/val/jtTest/S4_Dataset.csv"
tools::md5sum(snpfname)
```

```
## /data1/workspace/CALGB80303/GWAS/eQTL/val/jtTest/S4_Dataset.csv
##                               "0dd8499d2f2ce496d3067806953b3dd1"
```

```
c80303top50 <- read.csv(snpfname)
rownames(c80303top50) = c80303top50$rsid
rsInfo = c80303top50
```

Export annotation for writing to HTML file.

```
TopGenes <- matrix(rsInfo[TopSNPs,"GENESYMBOL"], nrow=topX)
TopGenesR <- matrix(rsInfo[TopSNPs, "PRECEDESYMBOL"], nrow=topX)
TopGenesL <- matrix(rsInfo[TopSNPs, "FOLLOWSYMBOL"], nrow=topX)
```

Change blank space to NA

```
TopGenesL[which(is.na(TopGenesL))]=""
TopGenesL[which(TopGenesL=="")]=NA
TopGenesR[which(is.na(TopGenesR))]=""
TopGenesR[which(TopGenesR=="")]=NA
```

Save the Top 50 Hits per Protein to HTML File (Continued...)

Generate output strings for HTML file.

```
htmlout <- paste0(TopSNPs,"<br/>",sprintf("%0.1e", TopPs),"<br/>", TopGenes)
GnaLR <- which(is.na(TopGenes)&(!is.na(TopGenesL)&!is.na(TopGenesR)))
htmlout[GnaLR] <- paste0(TopSNPs,"<br/>",sprintf("%0.1e", TopPs),"<br/>", TopGenesL,"<br/>",TopGenesR)[GnaLR]
GnaLnaR <- which(is.na(TopGenes)&is.na(TopGenesL)&!is.na(TopGenesR))
htmlout[GnaLnaR] <- paste0(TopSNPs,"<br/>",sprintf("%0.1e", TopPs),"<br/>", "NA","<br/>",TopGenesR)[GnaLnaR]
GnaLRna <- which(is.na(TopGenes)&!is.na(TopGenesL)&is.na(TopGenesR))
htmlout[GnaLRna] <- paste0(TopSNPs,"<br/>",sprintf("%0.1e", TopPs),"<br/>", TopGenesL,"<br/>","NA")[GnaLRna]
htmlout <- matrix(htmlout, nrow=topX)
colnames(htmlout) <- colnames(jtAll$J)
rownames(htmlout) <- 1:topX
```

Write to HTML file.

```
library(xtable)
hfile <- paste0(outdir,"C80303_fastJT_Top",topX,"SNPs.html")
print(xtable(htmlout), type="html", file=hfile,
  include.rownames=T,
  html.table.attributes='border=1 style="border-collapse:collapse" cellpadding="5"')
```

Save the Top 50 Hits per Protein to HTML File (Continued...)

Edit formatting on the HTML file

```
tx  <- readLines(hfile)
tx1  <- gsub(pattern = "&lt;", replace = "<", x = tx)
tx2  <- gsub(pattern = "&gt;", replace = ">", x = tx1)
writeLines(tx2, con=hfile)
tools::md5sum(hfile)


##     ./C80303_fastJT_Top50SNPs.html
## "2017ac7b971cabe9577807cd83db92d6"
```

Appendix

- R version 3.3.3 (2017-03-06), `x86_64-pc-linux-gnu`
- Base packages: base, datasets, graphics, grDevices, methods, stats, utils
- Other packages: clinfun 1.0.11, fastJT 1.0.2, gdata 2.17.0, GenABEL 1.8-0, GenABEL.data 1.0.0, knitr 1.13, MASS 7.3-44, microbenchmark 1.4-2.1, Rcpp 0.12.5, xtable 1.8-2
- Loaded via a namespace (and not attached): colorspace 1.3-2, evaluate 0.10, formatR 1.4, ggplot2 2.2.1, grid 3.3.3, gtable 0.2.0, gtools 3.5.0, highr 0.6, lazyeval 0.2.0, magrittr 1.5, munsell 0.4.3, mvtnorm 1.0-6, plyr 1.8.4, scales 0.4.1, stringi 1.1.3, stringr 1.2.0, tibble 1.3.0, tools 3.3.3

```
## [1] "Start Time Wed Apr  5 14:57:10 2017"
## [1] "End Time   Wed Apr  5 15:01:02 2017"
```

Supplementary Datasets Code

Genetic analysis of circulating angiogenesis proteins in cancer patients

Supplementary Datasets

Innocenti *et al.*

September 20, 2017

Introduction

These slides compile the individual .csv files containing the deidentified data used in the analysis slides into a single Microsoft Excel workbook.

```
library(openxlsx)
datdir <- "/data1/workspace/CALGB80303/GWAS/eQTL/Data/DIdata/"
```

CALGB 80303 Clinical Data

```
csv1 <- paste0(datdir, "Supplemental Dataset 1.csv")
tools::md5sum(csv1)

## /data1/workspace/CALGB80303/GWAS/eQTL/Data/DIdata/Supplemental Dataset 1.csv
##                                             "750f6f8c0a6be9a384ad79004aa6ee23"

ds1 <- read.csv(file=csv1, header=T,
                stringsAsFactors=FALSE)
dim(ds1)

## [1] 216   6

colnames(ds1)

## [1] "SUBJID"          "age"             "ps_new"          "dzext_new"
## [5] "priorrt_new"     "TREAT_ASSIGNED"
```

CALGB 80303 Plasma Markers

```
csv2 <- paste0(datdir, "Supplemental Dataset 2.csv")
tools::md5sum(csv2)

## /data1/workspace/CALGB80303/GWAS/eQTL/Data/DIdata/Supplemental Dataset 2.csv
##                                            "fe806115c878cfd07f423586584b4734"

ds2 <- read.csv(file=csv2, header=T,
                stringsAsFactors=FALSE)
dim(ds2)

## [1] 216  32

colnames(ds2)

##  [1] "X"          "FGFb"       "VEGF.C"     "PlGF"       "GROa"
##  [6] "PDGF.AA"    "SDF.1b"     "VEGF.D"     "IL.6"       "HGF"
## [11] "P.Selectin" "MCP.1"      "OPN"        "TSP.2"      "Ang2"
## [16] "IGFBP.1"    "IGFBBP.3"   "PAI1.tot"   "ICAM.1"     "PEDF"
## [21] "VEGF.R2"    "PAI1.act"   "VEGF.R1"    "VCAM.1"     "CRP"
## [26] "TGFb1"      "TGFb2"      "VEGF"       "PDGFbb"     "IL.8"
## [31] "IGF.1"      "TGFb.R3"

colnames(ds2)[1] <- "SUBJID"
```

CALGB 80203 Data

```
csv3 <- paste0(datdir, "Supplemental Dataset 3.csv")
tools::md5sum(csv3)

## /data1/workspace/CALGB80303/GWAS/eQTL/Data/DIdata/Supplemental Dataset 3.csv
##                                            "b4ef9c440fdfb960b66ce78b1813ac07"

ds3 <- read.csv(file=csv3, header=T,
                stringsAsFactors=FALSE)
dim(ds3)

## [1] 238  22

colnames(ds3)

##  [1] "SUBJID"          "RACE"            "ETHNICITY"       "AGE"
##  [5] "SEX"             "PERFORMANCE_ID" "LOCAL_META"      "TREAT_ASSIGNED"
##  [9] "rs2284284"       "rs7767396"       "rs7504372"       "MCP1"
## [13] "VEGF"            "VEGFC"           "VEGF.121"        "VEGF.145"
## [17] "VEGF.189"        "totalVEGF"       "VEGFA165"        "PGelg"
## [21] "PLelg"           "TUelg"
```

CALGB 80303 SNP Annotation

```
csv4 <- paste0(datdir, "Supplemental Dataset 4.csv")
tools::md5sum(csv4)

## /data1/workspace/CALGB80303/GWAS/eQTL/Data/DIdata/Supplemental Dataset 4.csv
##                                             "0dd8499d2f2ce496d3067806953b3dd1"

ds4 <- read.csv(file=csv4, header=T,
                stringsAsFactors=FALSE)
dim(ds4)

## [1] 1460   11

colnames(ds4)

##  [1] "rsid"          "chr"           "start"         "end"
##  [5] "LOCATION"      "GENEID"        "PRECEDEID"     "FOLLOWID"
##  [9] "GENESYMBOL"    "PRECEDESYMBOL" "FOLLOWSYMBOL"
```

Write out workbook

```
outfile <- paste0(datdir,"Supplemental Datasets.xlsx")
write.xlsx(list("CALGB 80303 Clinical Data"=ds1,
                "CALGB 80303 Plasma Markers"=ds2,
                "CALGB 80203 Data"=ds3,
                "CALGB 80303 SNP Annotation"=ds4 ),
                file=outfile)
tools::md5sum(outfile)

## /data1/workspace/CALGB80303/GWAS/eQTL/Data/DIdata/Supplemental Datasets.xlsx
##                                              "c39563f8b3bf809624fd3e8ebff74044"
```

Session Information

- R version 3.4.1 (2017-06-30), `x86_64-pc-linux-gnu`
- Running under: `Ubuntu 16.04.3 LTS`
- Matrix products: default
- BLAS: `/usr/lib/atlas-base/atlas/libblas.so.3.0`
- LAPACK: `/usr/lib/openblas-base/liblapack.so.3`
- Base packages: base, datasets, graphics, grDevices, stats, utils
- Other packages: knitr 1.16, openxlsx 4.0.17
- Loaded via a namespace (and not attached): compiler 3.4.1, evaluate 0.10.1, highr 0.6, magrittr 1.5, methods 3.4.1, Rcpp 0.12.12, stringi 1.1.5, stringr 1.2.0, tools 3.4.1

```
## [1] "Start Time Wed Sep 20 10:38:32 2017"
## [1] "End Time   Wed Sep 20 10:38:34 2017"
```
